# A Bayesian network and heuristic approach for systematic characterization of radiotherapy receipt after breast-conservation surgery

**DOI:** 10.1186/s12911-017-0479-4

**Published:** 2017-06-28

**Authors:** Milton Soto-Ferrari, Diana Prieto, Gitonga Munene

**Affiliations:** 1 Health Systems Decision Support Laboratory (HSDS), Industrial and Entrepreneurial Engineering & Engineering Management Department, Western Michigan University, 4601 Campus Drive, Kalamazoo, 49008 MI USA; 2 Western Michigan University School of Medicine, 1000 Oakland Drive, Kalamazoo, 49008 MI USA

**Keywords:** Bayesian network, Systematic, Heuristic

## Abstract

**Background:**

Breast-conservation surgery with radiotherapy is a treatment highly recommended by the guidelines from the National Comprehensive Cancer Network. However, several variables influence the final receipt of radiotherapy and it might not be administered to breast cancer patients. Our objective is to propose a systematic framework to identify the clinical and non-clinical variables that influence the receipt of unexpected radiotherapy treatment by means of Bayesian networks and a proposed heuristic approach.

**Methods:**

We used cancer registry data of Detroit, San Francisco-Oakland, and Atlanta from years 2007–2012 downloaded from the Surveillance, Epidemiology, and End Results Program. The samples had patients diagnosed with in situ and early invasive cancer with 14 clinical and non-clinical variables. Bayesian networks were fitted to the data of each region and systematically analyzed through the proposed Zoom-in heuristic. A comparative analysis with logistic regressions is also presented.

**Results:**

For Detroit, patients under stage 0, grade undetermined, histology lobular carcinoma in situ, and age between 26−50 were found more likely to receive breast-conservation surgery without radiotherapy. For stages I, IIA, and IIB patients with age between 51−75, and grade II were found to be more likely to receive breast-conservation surgery with radiotherapy. For San Francisco-Oakland, patients under stage 0, grade undetermined, and age >75 are more likely to receive BCS. For stages I, IIA, and IIB patients with age >75 are more likely to receive breast-conservation surgery without radiotherapy. For Atlanta, patients under stage 0, grade undetermined, year 2011, and primary site C509 are more likely to receive breast-conservation surgery without radiotherapy. For stages I, IIA, and IIB patients in year 2011, and grade III are more likely to receive breast-conservation surgery without radiotherapy.

**Conclusion:**

For in situ breast cancer and early invasive breast cancer, the results are in accordance with the guidelines and very well demonstrates the usefulness of the Zoom-in heuristic in systematically characterizing a group receiving a treatment. We found a subset of the population from Detroit with ductal carcinoma in situ for which breast-conservation surgery without radiotherapy was received, but potential reasons for this treatment are still unknown.

**Electronic supplementary material:**

The online version of this article (doi:10.1186/s12911-017-0479-4) contains supplementary material, which is available to authorized users.

## Background

Each year, approximately 40,450 women in the US are expected to die from breast cancer (BC) [1]. Medical treatment for BC are mainly driven by clinical factors including cancer staging, tumor size, histology, age, and primary site. The cancer staging is a critical variable since it provides information regarding the level of invasiveness of the cancer. Stage 0 (or in situ) is a non-invasive BC and is pathologically dissimilar to the stages of I, IIA, and IIB, which indicate an early degree of invasiveness.

Two of the primary treatments for in situ and early stage invasive cancer are breast-conservation surgery without radiotherapy (BCS), and breast-conservation surgery with radiotherapy (BCSR) [2]. For in situ, BCSR is a highly recommended treatment as it leads to significant clinical benefits, with an overall 16% absolute decrease in the risk of breast cancer recurrence and a 4% absolute decrease in the risk of dying from breast cancer [3, 4]. Although clinical factors drive the initial treatment selection, other considerations define the final treatment receipt. BCS might be selected over BCSR when there are contraindications to radiotherapy, or when the patient declined its implementation [1, 5–8].

Perceived risks can also influence the treatment, as concerns of recurrence after BCS might persuade a patient to choose BCSR. On the other hand, concerns of side effects after radiotherapy may result in the use of BCS. Perceived treatment risks can be additionally driven by a set of non-clinical factors that include insurance status, poor access to care, socioeconomic status, race, education, age, and marital status [5, 9]. Concerns of Medicare coverage for radiotherapy may drive patients towards BCS, due to the inability to afford the out of pocket costs for transportation to the radiotherapy center, or by the long distance of a patient’s residence from the radiotherapy facility [10]. Older patients (e.g., >75) are prone to side effects that might be life threatening, which leads them to decline radiotherapy and accept the use of palliative treatments [11, 12].

As the previous perceived risks may lead to the inappropriate receipt of a treatment, identification of patients with unexpected radiotherapy treatment recommendation is a subject of interest for healthcare providers. Unexpected radiotherapy treatment may occur when a patient’s clinical status might indicate the use of BCSR but the treatment received was BCS, or when the patient is expected to be treated with BCS but receives BCSR. Identification of the variables or features of the patients receiving unexpected radiotherapy treatment can help physicians to identify those patients in need of more targeted education about their treatment, or of additional support for the procurement of human, logistic, or financial resources for treatment.

Models including statistical methods such as logistic and multivariate regressions had been used to identify the variables affecting the receipt of a treatment [4, 5, 13–18]. In these models, the significance of a variable effect is often evaluated with odds ratios where a relation is only established in categories within variables, which limit a more advanced exploration of possible relations between other categories and variables. These model formulations are non-hierarchical, meaning that the cause and effect relationships among the variables are not explicitly represented, creating misrepresentation that may produce bias in the variable effects. Most of these studies assume that the expected treatment is always BCSR and therefore a high receipt of BCS can be considered as an indicator of an unexpected radiotherapy treatment. However, as described before, treatment decisions are influenced by a variety of clinical and non-clinical reasons that might invalidate the general interpretation that is usually provided. There also exists hierarchical methods to determine the significance of variables in the treatment receipt but they have not been used to analyze influential variables in differential treatment [19–22].

Our objective with this research is to propose a systematic framework to identify the clinical and non-clinical variables that influence the receipt of unexpected radiotherapy treatment for in situ and early stage invasive cancer. The identification of the variables is conducted by means of a Bayesian network (BN) to identify associations among clinical and non-clinical variables, and a proposed Zoom-in heuristic to reduce the exploration of variables influencing unexpected radiotherapy treatment. We demonstrate how the BN-Zoom-in approach can be used to determine the clinical features that drive a patient to receive BCS or BCSR. We also demonstrate how branching the heuristic can help to identify unexpected treatments. Information from the Surveillance, Epidemiology, and End Results Program (SEER) is used to populate the models.

Our approach provides the following contributions: 1) The implementation of BN models mapping the receipt of radiotherapy treatment using clinical and non-clinical variables. To our knowledge, previous BN models have been built to diagnose the type of cancer or to quantify tumor progression occurring in the breast [23–26], but we are not aware of analyses that attempt to explain the receipt of a treatment not only from the clinical standpoint but also from the non-clinical variables available in the SEER dataset. 2) The development of a Zoom-in heuristic to facilitate model exploration. With the BN model only, it might be complex to explore meaningful variable associations as the space of these associations increases with the number of variables. Existing model exploration takes place by observation of the entire universe of associations, a process that is sometimes guided by intuition and expert knowledge. With the proposed framework, time and effort is reduced by implementing the Zoom-in heuristic after the BN model is fitted.

## Methods

### Bayesian network structure

A BN is a class of graphical model that allows a concise representation of the probabilistic dependencies between a given set of random variables [27]. Consider *G*=(*V,A*) a directed acyclic graph (DAG) where each node *v*∈*V* corresponds to a random variable and A are the arcs connecting the nodes. In a BN, the probabilistic dependencies can be mapped with the following joint distribution formulation:

1 $$\begin{array}{@{}rcl@{}} P(V) &=& \prod_{v \in V} p (v|pa(v)) \end{array} $$

Where *pa*(*v*) is the set of the parents of *v*.

In our analysis, the BN will map the relations between clinical and non-clinical factors to determine the type of treatment that a patient is likely to receive (i.e., BCS, BCSR). Design of a BN requires a structure of conditional dependence and conditional probability tables (CPTs) for each node in the network. For the creation of our BN we considered three sequential steps:

i) Data-driven learning: A Bayesian score-based hill-climbing learning procedure is conducted to search and orient the edges, and infer causal structures for the construction of the BN skeleton. We used the score based hill-climbing learning algorithm to explore the search space (i.e., arc-node combinations) starting from an empty graph. At each iteration, all the possible additions, deletions or arc reversals are evaluated to obtain a better structured network that is measured by an optimization score, the final BN skeleton is obtained once the optimization score is no longer improved. The Rpackage bnlearn was used to create the BN using 1000 iterations and an average threshold of 0.25. More information regarding the hill-climbing learning procedure and the Rpackage bnlearn can be found in [27].
ii) Validation of dependency relationships: In this step, the data-driven BN is validated by our collaborators in surgery and oncology (refer to “Acknowledgments” section). To discuss the BN with the physicians, a semi-structured interview was conducted to evaluate the dependency relationships generated by the hill-climbing algorithm. We presented the resulting network to our collaborating physicians, and we asked them to interpret the arc-node combinations as possible cause-effect relationships. If the physicians felt that a pair of variables or factors were displaying an unreasonable association, the arc-node combination was discussed. All additions, deletions or arc reversals suggested by the physicians were documented to further update the BN model, if necessary.
iii) Model fitting: Conditional probabilities are computed in the BN to create conditional probability tables (CPTs). Probabilities are computed using propagation methods and the message passing procedure, with the Rsoftware package “grain” [28].

### Data collection

We obtained the data from the SEER program. We included all the available female residents from the metropolitan Detroit area that were diagnosed with breast cancer and treated with BCS or BCSR between 2007 and 2012. The sample had a total of 11736 patients classified with the American Joint Committee on Cancer (AJCC) staging (levels 0, I, IIA, IIB). We extracted and categorized non-clinical information including race/ethnicity (**R**), marital status (**M**), insurance type (**IN**), willingness to receive radiotherapy (**W**), and year of diagnosis (**Y**). These non-clinical variables were included since they were perceived as significant by the clinicians.

Similarly, we processed clinical information including age at diagnosis (**A**), histology (**H**), grade (**G**), primary site (**P**), AJCC stage (**S**), degree of tumor invasiveness (**I**), tumor behavior (**B**), tumor size (**TS**), and type of treatment (**T**) received by the patients (i.e., BCS, BCSR). These variables were selected from the SEER dataset as indicated by our collaborating clinicians. Table 1 shows in detail the information and categories for each of the variables related to the type of treatment received by the patient.

**Table 1:** Patient and tumor characteristics according to treatment type BCS and BCSR stages 0-II breast cancer 2007–2012 in metropolitan Detroit area (SEER)

	Breast-conservation surgery			Breast-conservation surgery		
	without radiotherapy (BCS)			with radiotherapy (BCSR)		
Variable	No	Row%		No	Row%	Total
All Patients	2517	(21.4)		9219	(78.6)	11736
Race/Ethnicity (R)						
White	1860	(21.0)		7007	(79.0)	8867
Black	603	(23.5)		1964	(76.5)	2567
Other	37	(16.6)		186	(83.4)	223
Unknown	17	(21.5)		62	(78.5)	79
Age at diagnosis (A)						
≤25	2	(40.0)		3	(60.0)	5
26−50	480	(20.7)		1843	(79.3)	2323
51−75	1279	(17.3)		6096	(82.7)	7375
>75	756	(37.2)		1276	(62.8)	2032
Unknown	0	(0.0)		1	(100.0)	1
Marital status (M)						
Single	347	(22.9)		1168	(77.1)	1515
Married	1145	(18.6)		5022	(81.4)	6167
Separated	19	(24.4)		59	(75.6)	78
Divorced	228	(18.8)		982	(81.2)	1210
Widowed	507	(30.4)		1161	(69.6)	1668
Unmarried	0	(0.0)		2	(100.0)	2
Unknown	271	(24.7)		825	(75.3)	1096
Insurance status (IN)						
Uninsured	24	(21.6)		87	(78.4)	111
Medicaid	156	(25.5)		456	(74.5)	612
Insured	2277	(21.0)		8573	(79.0)	10850
Unknown	60	(36.8)		103	(63.2)	163
Year (Y)						
2007	491	(25.5)		1433	(74.5)	1924
2008	440	(23.5)		1433	(76.5)	1873
2009	434	(22.5)		1494	(77.5)	1928
2010	340	(18.5)		1500	(81.5)	1840
2011	417	(20.6)		1607	(79.4)	2024
2012	395	(18.4)		1752	(81.6)	2147
AJCC Stage (S)						
0	1123	(33.5)		2230	(66.5)	3353
I	826	(15.2)		4613	(84.8)	5439
IIA	413	(19.1)		1752	(80.9)	2165
IIB	155	(19.9)		624	(80.1)	779
Grade						
I	519	(18.5)		2290	(81.5)	2809
II	787	(17.0)		3848	(83.0)	4635
III	534	(16.9)		2633	(83.1)	3167
IV	10	(21.7)		36	(78.3)	46
Undetermined	667	(61.8)		412	(38.2)	1079
Tumor size (TS)^a^						
≤5	434	(24.7)		1326	(75.3)	1760
6−11	481	(16.3)		2478	(83.7)	2959
12−17	422	(16.3)		2161	(83.7)	2583
18−23	272	(18.2)		1223	(81.8)	1495
24−29	173	(20.4)		674	(79.6)	847
30−35	119	(20.3)		466	(79.7)	585
36−41	62	(24.1)		195	(75.9)	257
42−47	27	(20.0)		108	(80.0)	135
48−53	36	(32.1)		76	(67.9)	112
54−59	16	(38.1)		26	(61.9)	42
60−65	10	(35.7)		18	(64.3)	28
66−71	5	(29.4)		12	(70.6)	17
72−77	4	(36.4)		7	(63.6)	11
78−83	3	(33.3)		6	(66.7)	9
84−89	9	(81.8)		2	(18.2)	11
90−95	0	(0.0)		4	(100.0)	4
96−101	4	(57.1)		3	(42.9)	7
>101	5	(55.6)		4	(44.4)	9
Unknown	435	(50.3)		430	(49.7)	865
Histology (H)^b^						
8201	97	(24.0)		307	(76.0)	404
8211	24	(26.7)		66	(73.3)	90
8230	41	(15.5)		223	(84.5)	264
8480	39	(20.7)		149	(79.3)	188
8500	1212	(16.7)		6026	(83.3)	7238
8501	38	(13.7)		240	(86.3)	278
8503	25	(25.5)		73	(74.5)	98
8507	22	(25.9)		63	(74.1)	85
8520	639	(53.7)		551	(46.3)	1190
8522	78	(16.2)		403	(83.8)	481
8523	215	(19.1)		909	(80.9)	1124
Other	87	(29.4)		209	(70.6)	296
Behavior (B)						
In situ	1120	(33.5)		2224	(66.5)	3344
Invasive	1397	(16.6)		6995	(83.4)	8392
Willingness/radiotherapy (W)^c^						
Not declined	2423	(20.8)		9219	(79.2)	11642
Declined	94	(100.0)		0	(0.0)	94
Primary Site (P)						
C500	21	(32.3)		44	(67.7)	65
C501	147	(25.5)		429	(74.5)	576
C502	269	(19.6)		1101	(80.4)	1370
C503	151	(20.6)		581	(79.4)	732
C504	849	(18.8)		3668	(81.2)	4517
C505	162	(19.0)		689	(81.0)	851
C506	16	(25.0)		48	(75.0)	64
C508	539	(20.9)		2038	(79.1)	2577
C509	363	(36.9)		621	(63.1)	984
Degree/invasiveness (I)^d^						
F0	541	(18.8)		2342	(81.2)	2883
F10	1119	(33.5)		2223	(66.5)	3342
F20	779	(15.5)		4255	(84.5)	5034
F30	26	(15.8)		139	(84.2)	165
F40	8	(13.6)		51	(86.4)	59
F50	15	(19.5)		62	(80.5)	77
F60	1	(100.0)		0	(0.0)	1
F987	28	(16.0)		147	(84.0)	175

^a^Tumor size is measure in ml

^b^ICD-O-3 Nomenclature: **8201** Cribriform carcinoma in situ, **8211** Tubular adenocarcinoma, **8230** Duct carcinoma in situ, solid type, **8480** Mucinous adenocarcinoma, **8500** Intraductal carcinoma, noninfiltrating, **8501** Comedocarcinoma, non-infiltrating, **8503** Noninfiltrating intraductal papillary adenocarcinoma, **8507** Intraductal micropapillary carcinoma, **8520** Lobular carcinoma in situ, **8522** Intraductal and lobular in situ carcinoma, **8523** Infiltr. duct mixed with other types of carcinoma, in situ

^c^Willingness radiotherapy, is based upon the field denominated “RX SUMM–RADIATION” on the SEER data, from here the option of Patient refused radiation therapy were considered as “Declined"

^d^Degree of tumor invasiveness was categorized with the field CS Site-Specific Factor 6 from SEER. The field records the code that indicates how the pathological tumor size was coded in tumor size. codification includes the levels: **F0** Where the entire tumor reported as invasive, **F10** Entire tumor reported as in situ, **F20** Invasive and in situ components present (size of invasive component stated and coded in tumor size), **F30** Invasive and in situ components present (size of entire tumor coded in tumor size because size of invasive component not stated and in situ described as minimal), **F40** Invasive and in situ components present (size of entire tumor coded in tumor size because size of invasive component not stated and in situ described as extensive), **F50** Invasive and in situ components present (size of entire tumor coded in tumor size because size of invasive component not stated and proportions of in situ and invasive not known), **F60** Invasive and in situ components present (unknown tumor size coded), **F987** Unknown if invasive and in situ components present

### Bayesian network design

For learning and testing of the network we implemented a 70/30 setting where a sample of 8217 cases was used to perform the learning of the structure (i.e., data-driven learning), and the remaining 3519 cases were used for testing of the network. Each case included the 14 non-clinical and clinical variables collected. After the data-driven learning the physicians validated the BN without any suggested modifications. The joint distribution of the variables over the BN its expressed by the following function:

2 $${} \begin{array}{c} P(V) = P(G|TS,S,B,R)\,P(T|A,G,B) \,P(H|T,G,B) \,P(S|I,B) \\[\jot] \qquad \qquad \ \; P(W|A,T)\ \; P(B|I,A) \ \; P(M|A) \ \; P(Y|T) \ \; P(P|B)\\[\jot] \qquad \qquad \ \; P(I|A) \ \; P(R|M) \ \; P(IN|M) \ \; P(TS|S) \ \; P(A) \end{array}  $$

**Fig. 1:**
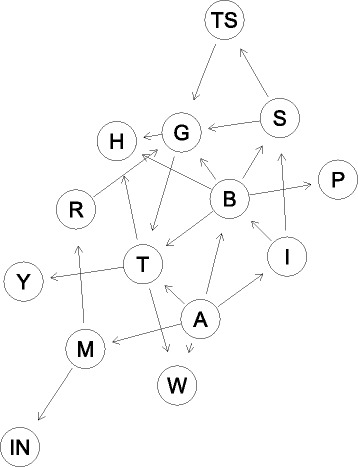
Bayesian network for Detroit

Figure 1 describes the mapped BN over the DAG. For the testing of the BN we calculated the prediction performance of the network by means of Accuracy, Sensitivity, and Specificity. “Discussion” section further compares these performance values with those of a logistic regression fitted with the same dataset.

### Exploration of influential variables

To explore the arc-node combinations in the BN, and conclude over influential variables in the receipt of a differential treatment, we developed an iterative heuristic to explore the conditional probabilities over the variables in the BN. We denominated this procedure the Zoom-in heuristic since we seek to iteratively narrow down the subset of patients that are more likely to receive a specific treatment.

#### Zoom-in Heuristic

Let *V*_*i*_ be a random variable where *v*_*i*_∈*V*_*i*_ and *v*_*i*_ denotes the value of the category *i*(*i*=1…,*n*) for a variable *V*. Consider *C* as the set of clinical variables such that *C*∈*V*, and consider the random variable *C*_*i*_ where an outcome *c*_*i*_ denotes the value of the category *i* for a clinical variable *C*.

$$\begin{array}{@{}rcl@{}} &&\text{1) Set~} v_{i}^{*} = \{0\} \\ &&\text{2) If} ~P\left(C_{i}=c_{i} \, | \, S=s,T=BCS\right) \neq P(C_{i}=c_{i} \, | \,S=s, \\ &&~~~~~~~~~~~~~T=BCSR) \\ &&\qquad \text{Then~} c_{i} \rightarrow {v_{i}^{*}} \\ &&\qquad \text{Repeat for all combinations of~} C, C_{i} \text{, and }S.\\ &&\text{3) If} ~P\left(\left(v_{i} \in V_{i}\right) \, \cup \, (v_{i} \notin v_{i}^{*}) \, | \, S=s,T=BCS,v_{i}^{*}\right) \neq\\ &&\qquad P\left((v_{i} \in V_{i}) \, \cup \, (v_{i} \notin v_{i}^{*}) \, | \, S=s,T=BCSR,v_{i}^{*}\right)\\ &&\qquad \text{Then~} v_{i} \rightarrow {v_{i}^{*}} \\ &&\qquad \text{Repeat for all combinations of~} V, V_{i} \text{, and} S.\\ &&\text{4) Repeat step 3 until~} v_{i}^{*} \text{is no longer updated.} \\ \end{array} $$

In the first step, we defined an empty set of categories denominated $v_{i}^{*}$.

In the second step, we compare the probabilities of clinical variables categories given a combination of stage and treatment. For this step, only clinical variables are included as they should be the most relevant predictors for treatment. If a category of a variable was judged to be different, this category is included in the set $v_{i}^{*}$. This process is repeated for all combinations of clinical variables, categories, and stages.

In the third step, we compare the probability of an outcome not previously selected for $ v_{i}^{*}$ given a combination of stage, treatment, and a specific instance of the set $ v_{i}^{*}$. All variables (i.e., clinical and non-clinical) are now included in the analysis. If a category of a variable was judged to be different, this category is also included in the set $v_{i}^{*}$.

In the second and third steps, differences between probabilities are judged using a tolerance of 0.04 (i.e., if the difference between two categories of a variable are greater than the established tolerance the category is judge to be different).

In the fourth step, step 3 is performed until we cannot find any other differences between probabilities. The final $v_{i}^{*}$ set corresponds to the most common features of patients that are receiving a target treatment.

## Results

### Implementation of the Zoom-in Heuristic

We describe the results of implementing the BN-Zoom-in for the patients’ population in the Detroit area using the SEER data. We also present implementation results for populations of San Francisco-Oakland and Metropolitan Atlanta as examples of generalization of the BN-Zoom-in approach. The reader is referred to the “Discussion” section for an interpretation of these results, and a comparison of the BN-Zoom-in with Logistic regression models, which is the most traditional method for determining potential influential variables in treatment receipt.

#### In Situ BC

The following is the implementation of the Zoom-in heuristic when the target treatment is BCS for stage 0 patients in the Detroit area. For step 2 of the Zoom-in heuristic, the evaluation results for stage 0 are showed in Table 2. Table 2 shows *P*(*G*=*g*|*S*=0,*T*={*BCS,BCSR*}), for all the outcomes of the clinical variable grade (i.e., I, II, III, IV, and undetermined). Only, *P*(*G*=*undetermined*|*S*=0,*T*=*BCS*) and *P*(*G*=*undetermined*|*S*=0,*T*=*BCSR*) were judged to be different.

**Table 2:** Conditional probabilities for Grade (G) given Stage = 0

Grade (G)	I	II	III	IV	Undetermined
*S*=0,*T*=*BCS*	0.144	0.226	0.114	0.003	0.512
*S*=0,*T*=*BCSR*	0.169	0.405	0.329	0.014	0.083

For this step, category Undetermined is judged as different

For step 3 of the heuristic, Tables 3 and 4 present the results for stage 0 and grade undetermined. Tables 3 and 4, show *P*(*H*=*h*|*S*=0,*G*=*undetermined*, *T*={*BCS,BCSR*}), for all the outcomes of the clinical variable histology (e.g., lobular carcinoma in situ is denoted as “8520”. Refer to the comments in Table 1 for additional details). Only, *P*(*H*=8520|*S*=0,*G*=*undetermined,T*=*BCS*) and *P*(*H*=8520|*S*=0,*G*=*undetermined,T*=*BCSR*) were judged to be different.

**Table 3:** Conditional probabilities for Histology (H) given Stage = 0 and Grade = Undetermined

Histology (H)	8201	8211	8230	8480	8500	8501
*S*=0,*G*=*Undetermined,T*=*BCS*	0.005	<0.001	0.005	<0.001	0.069	0.013
*S*=0,*G*=*Undetermined,T*=*BCSR*	0.079	<0.001	0.024	0.001	0.399	0.079

**Table 4:** Conditional probabilities for Histology (H) given Stage = 0 and Grade = Undetermined

Histology (H) (continued)	8503	8507	8520	8522	8523	Other
*S*=0,*G*=*Undetermined,T*=*BCS*	0.003	0.003	0.858	0.011	0.021	0.011
*S*=0,*G*=*Undetermined,T*=*BCSR*	0.056	0.008	0.059	0.048	0.221	0.026

For this step, category 8520 (LCIS) is judged as different

For step 4 of the heuristic, Table 5 shows *P*(*A*=*a*|*S*=0,*G*=*undetermined,H*=8520,*T*={*BCS,BCSR*}), for all the outcomes of the clinical variable age (i.e., ≤25, 26−50, 51−75, and >75). Only, *P*(*A*=26−50|*S*=0,*G*=*undetermined,H*=8520,*T*=*BCS*) and *P*(*A*=26−50|*S*=0,*G*=*undetermined,H*=8520,*T*=*BCSR*) were judged to be different.

**Table 5:** Conditional probabilities for Age (A) given Stage = 0, Grade = Undetermined, and Histology = 8520

Age (A)	≤25	26−50	51−75	>75	Unknown
*S*=0,*G*=*Undetermined, H*=8520,*T*=*BCS*	<0.001	0.266	0.609	0.125	<0.001
*S*=0,*G*=*Undetermined, H*=8520,*T*=*BCSR*	0.001	0.153	0.715	0.13	0.001

**Fig. 2:**
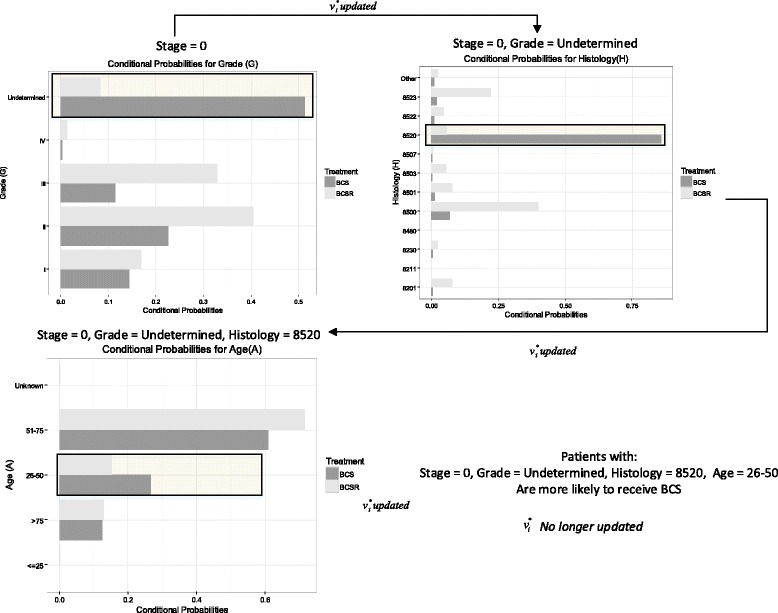
BN-Zoom-in approach for Detroit patients in Stage 0. Categories in the $v_{i}^{*}$ set are presented. For each iteration variables were evaluated to determine differences

Figure 2, graphically shows the step by step process of the heuristic for in situ BC. Results for Table 2 are depicted in the upper left chart titled “Stage 0”. Tables 3 and 4 are depicted in the upper right chart titled “Stage=0, Grade= Undetermined”. Results for Table 5 are in chart “Stage=0, Grade= Undetermined, Histology=8520”. Under these conditions, the age group “ 26−50” is judged as different and no more differences are found. Hence the patients under stage 0, grade undetermined, with histology of lobular carcinoma in situ (LCIS) (i.e., 8520), and with ages between 26 and 50 are declared as the group more likely to receive BCS (target treatment) for in situ BC. (Probabilities values for all the variables in each step of the BN-Zoom-in approach can be found in the Additional file 1: Section 1).

#### Early invasive BC

We now considered stages I, IIA, IIB for the target treatment BCSR. For step 2 of the Zoom-in heuristic, the evaluation results for stages I, IIA, IIB are showed in Table 6 (Only the variable for which a difference was found is presented). Table 6, shows *P*(*A*=*a*|*S*={*I,IIA,IIB*},*T*={*BCS,BCSR*}), for all the outcomes of the clinical variable age. Only, *P*(*A*=51−75|*S*={*I,IIA,IIB*},*T*=*BCS*) and *P*(*A*=51−75|*S*={*I,IIA,IIB*},*T*=*BCSR*) were judged to be different.

**Table 6:** Conditional probabilities for Age (A) given Stage = I, IIA, IIB

Age (A)	≤25	26−50	51−75	>75	Unknown
*S*=*I,T*=*BCS*	0.001	0.115	0.478	0.405	0.001
*S*=*I,T*=*BCSR*	0.001	0.192	0.646	0.161	<0.001
*S*=*IIA,T*=*BCS*	0.001	0.124	0.482	0.392	0.001
*S*=*IIA,T*=*BCSR*	0.001	0.194	0.645	0.160	<0.001
*S*=*IIB,T*=*BCS*	0.002	0.134	0.486	0.377	0.002
*S*=*IIB,T*=*BCSR*	0.001	0.203	0.641	0.155	<0.001

For this step, category **51−75** is judged as different for all stages

For step 3 of the heuristic, Table 7 presents the results for stages I, IIA, IIB and age 51−75. Table 7, shows *P*(*G*=*g*|*S*={*I,IIA,IIB*},*A*=51−75,*T*={*BCS,BCSR*}), for all the outcomes of the clinical variable grade. Only, *P*(*G*=*II*|*S*={*I,IIA,IIB*},*A*=51−75,*T*=*BCS*) and *P*(*G*=*II*|*S*={*I,IIA,IIB*},*A*=51−75,*T*=*BCSR*) were judged to be different. After this evaluation we found no other differences.

**Table 7:** Conditional probabilities for Grade (G) given, Stage = I, IIA, IIB, and Age = **51−75**

Grade (G)	I	II	III	IV	Undetermined
*S*=*I,A*=51−75,*T*=*BCS*	0.302	0.387	0.232	0.003	0.076
*S*=*I,A*=51−75,*T*=*BCSR*	0.329	0.428	0.202	<0.001	0.041
*S*=*IIA,A*=51−75,*T*=*BCS*	0.155	0.362	0.415	0.015	0.052
*S*=*IIA,A*=51−75,*T*=*BCSR*	0.176	0.417	0.376	0.002	0.029
*S*=*IIB,A*=51−75,*T*=*BCS*	0.105	0.342	0.482	0.028	0.043
*S*=*IIB,A*=51−75,*T*=*BCSR*	0.122	0.402	0.448	0.003	0.024

For this step, category II judged as different for all stages

**Fig. 3:**
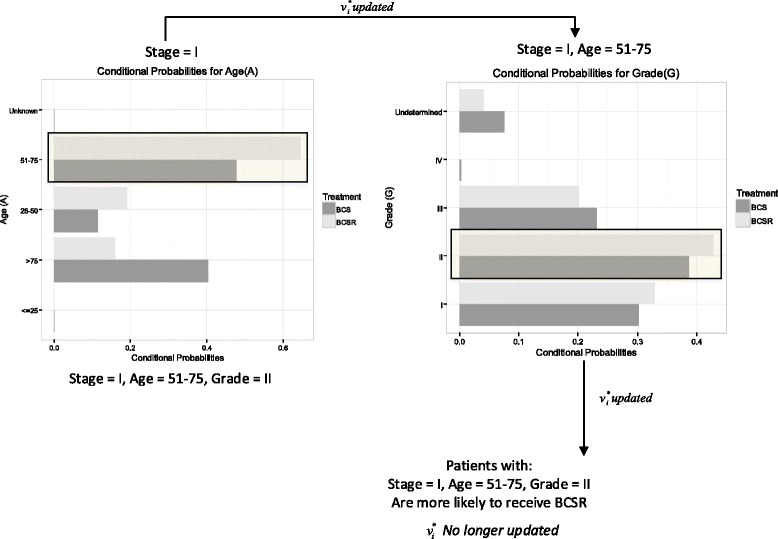
BN-Zoom-in approach for Detroit patients in Stage I. Categories in the $v_{i}^{*}$ set are presented. For each iteration variables were evaluated to determine differences

Figure 3, graphically shows the step by step process of the heuristic for stage I (These results are similar in values for the stages IIA and IIB). Results for Table 6 are depicted in the upper left chart titled “Stage I”. Table 7 are depicted in the upper right chart titled “Stage=I, Age= 51–75”. Under these conditions, grade “II" is judged as different and no more differences are found. Hence the patients under stages I, IIA, IIB, with ages between 51 and 75, and with grade II are declared as the group more likely to receive BCSR for early invasive BC. (Probabilities values for all the variables in each step of the BN-Zoom-in approach can be found in the Additional file 1: Section 1).

### Branching in the BN-Zoom-in to analyze unexpected treatment receipt

The Zoom-in heuristic identifies the most common conditions and features for which a patient might receive a target treatment. Patients at risk of a differential treatment might be detected by means of a branching in the analysis while performing the heuristic.

**Fig. 4:**
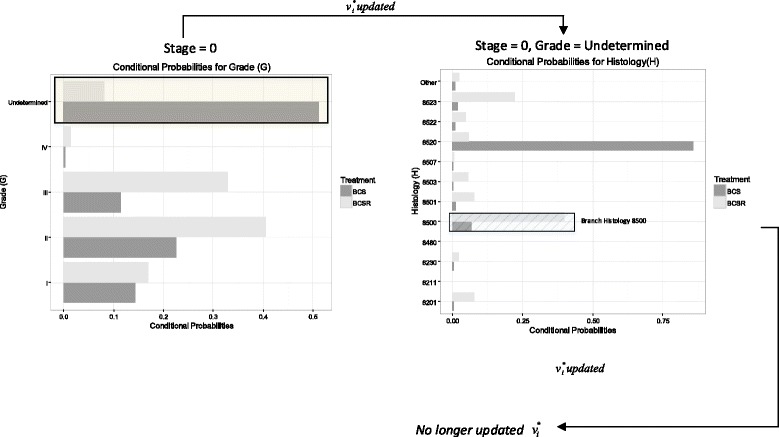
BN-Zoom-in approach for Detroit patients in Stage 0 (Branching). The branch is highlighted as a stripped box

For example, in the situ BC evaluation in Table 3, we identified that 6.9% (or 41 patients) receive BCS as primary treatment when histology is 8500 (i.e., ductalcarcinoma in situ (DCIS)) and grade is undetermined. Since BCS is an unexpected treatment when patients have a DCIS histology, we want to explore if other clinical or non-clinical variables influenced the receipt of this treatment. For this purpose we considered the evaluation of the branch *P*(*H*=8500|*S*=0,*G*=*undetermined,T*=*BCS*) in the Zoom-in heuristic. The branch is highlighted as a stripped box in Fig. 4. For this branch, neither non-clinical nor clinical variables were identified as different.

### Application of the BN-Zoom-in when controlling for stage

We performed controlling experiments to better understand the change in the influence of factors in the treatment when the variability due to stage is removed. When controlling per stage (i.e., building the BN only for patients in a specific stage status as opposed to all the patients without regards to their stage), we found the results in the first row of Table 8. For stage 0, the influential factors in the receipt of BCS are LCIS histology, unknown tumor size, and entire breast (C509) as the primary site. LCIS was found as influential for both the controlled and the entire dataset (see Fig. 2). In contrast to the model with the entire dataset, for stage 0, undetermined grade, and age 26−50 were not found as influential but unknown tumor size and entire breast as primary site were additional influential factors in this stage. When patients in stages I, IIA, and IIB are included in the BN independently, only age 51–75 was found as influential for the receipt of BCSR. This factor is also identified for the entire dataset (see Fig. 3). In contrast to the model with the entire dataset, grade II (i.e., moderately differentiated) does not appear as influential for any of the three stages.

**Table 8:** Significant variables controlling stage for BN-Zoom-in and logistic regression (LR)

Model	Stage 0	Stage I	Stage IIA	Stage IIB
BN-Zoom-in	Histology (8520:LCIS)	Age (51−75)	Age (51−75)	Age (51−75)
	Tumor size (Unknown)			
	Primary site (C509)			
LR	Histology (8520:LCIS)	Not influential variables	Not influential variables	Not influential variables
	Age (26−50)			
	Age (51−75)			

### BN-Zoom-in generalization

We implemented the BN-Zoom-in approach to the SEER information of San Francisco-Oakland and Atlanta. For each region, we developed an independent analysis since treatment administration rates can be geographically different [29]. In the U.S., it is believed that geography influences treatment due to the interaction of several factors, including patient’s socioeconomic and education level, as well as limited access and availability of healthcare resources. For example, the number of people below the federal poverty level, and therefore eligible to receive Medicaid covered treatment, is different per state. This might influence the rates of treatment since patients are unable to afford the out-of-pocket costs and they have limited ability to navigate through the agencies providing funding for cancer treatment.

For learning and testing of both networks we implemented a 70/30 setting and in each case we included the 14 non-clinical and clinical previously described.

#### San Francisco-Oakland

**Fig. 5:**
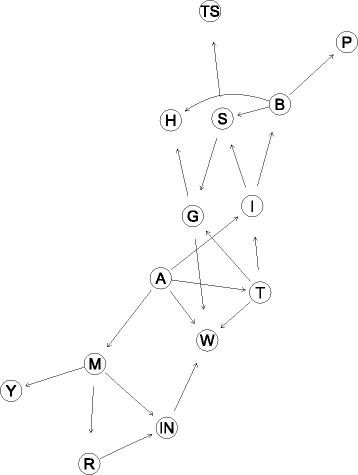
Bayesian network for San Francisco-Oakland

A sample of 13896 female patients in stages 0, I, IIA, IIB that received BCS and BCSR from the SEER dataset in the San Francisco-Oakland area is considered for this development. Where a set of 9730 cases was used to perform the learning of the structure (i.e., data-driven learning), and the remaining 4166 were used for testing of the network. After the data-driven learning the physicians validated the BN without any modifications. Figure 5 describes the mapped BN over the DAG. To validate and assess the prediction power of the BN, we built a logistic regression model (LR) with the same information provided by the SEER for San Francisco-Oakland, we calculated the Accuracy, Sensitivity, and Specificity parameters using a 70/30 setting, as with the BN. “Discussion” section further compares these performance values with those of a logistic regression fitted with the same dataset.

We performed the BN-Zoom-in approach to identify influence of factors in treatment decision. For the BN of San Francisco-Oakland we found that patients in stage 0, grade undetermined, and age >75 are more likely to receive BCS. For stages I, IIA, IIB patients with age >75 are more likely to receive BCS.

#### Atlanta

**Fig. 6:**
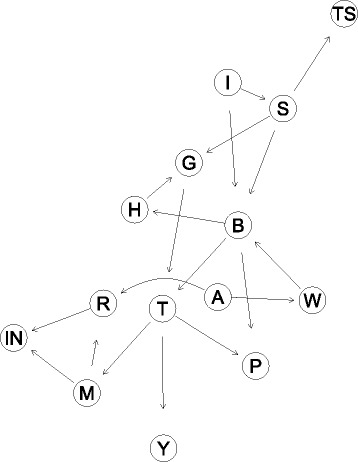
Bayesian network for Atlanta

A similar evaluation was performed for the Atlanta region, for this case a sample of 7893 female patients in stages 0, I, IIA, IIB that received BCS and BCSR is extracted from the SEER program. A set of 5528 cases was used to perform the learning of the structure (i.e., data-driven learning), and the remaining 2365 cases were used for testing of the network. After the data-driven learning the physicians validated the BN without any modifications. Figure 6 describes the mapped BN over the DAG. A LR is also build and the parameters Accuracy, Sensitivity and Specificity are calculated for both models. “Discussion” section further compares these performance values with those of a logistic regression fitted with the same dataset.

Regarding the influence of factors in the treatment after the implementation of the BN-Zoom-in approach, we found for the BN of Atlanta that patients in stage 0, grade undetermined, year 2011, and primary site C509 (i.e., entire breast) are more likely to receive BCS. For stages I, IIA, IIB patients in year 2011, and grade III are more likely to receive BCS.

## Discussion

We presented a systematic assessment of BC treatment receipt using a BN and the Zoom-in heuristic. We developed networks with 14 variables for demonstration purposes, and we believe our approach is highly advantageous for the systematic analysis of treatment receipt when many more variables (and categories per variable) are present. As mentioned before, previous BN models have been built to diagnose the type of cancer or to quantify tumor progression occurring in the breast [23–26]. However, the complexity associated with the analysis of BNs with several variables and categories per variable increases the difficulty to find meaningful relationships between variables that can concur in reasonable conclusions. The Zoom-in heuristic reduces this complexity by systematically identifying the common features of a specific condition of interest (treatment receipt in our case) and the subsequent branches.

To validate and assess the prediction power of the BN-Zoom-in approach, we built a logistic regression model (LR) with the same information provided by the SEER of Detroit (see Table 1). We ran an initial model including all the explanatory variables that were used for the BN-Zoom-in approach, and we calculated the Accuracy, Sensitivity, and Specificity parameters using a 70/30 setting, as with the BN (See the R output in Additional file 1: Section 2). Table 9 presents the results of these parameters for both the BN and the LR model. The global prediction power of the BN is statistically similar to the LR. The BN presents slightly higher Accuracy and Specificity and the LR model presents slightly higher Sensitivity (see Additional file 1: Section 3).

**Table 9:** Logistic regression and Bayesian network validation results (Detroit)

	Accuracy	Sensitivity	Specificity
BN	0.838	0.885	0.833
LR	0.819	0.943	0.813

The LR for Detroit shows that grade undetermined and histology 8520 (LCIS) are the most significant variables in the recommendation of BCS vs BCSR (see Additional file 1: Section 2). These results are in agreement with those obtained for the BN-Zoom-in in stage 0 (See Tables 2 and 4). With a single LR and the whole data set, we cannot provide a comprehensive analysis differentiating among stages. With the BN, this analysis is possible. Also, the analysis of LR is performed using odds ratios between two categories of one variable at a time. In contrast, the BN model can provide analysis of hierarchical relationships between more than two categories within and across variables, which leads to a more comprehensive analysis.

In our results for Detroit, patients under stage 0, grade undetermined, histology LCIS, and age between 26 and 50 were found more likely to receive BCS for in situ BC. For patients in stage 0 and grade undetermined, BCS can be recommended as the uncertainty in the aggressiveness of the cancer may lead physicians to be more cautious about radiotherapy. If the cancer is also categorized as LCIS, medical professionals tend to not recommend radiotherapy since it is generally not thought to be a precursor of invasive cancer [30]. LCIS patients are usually pre-menopausal [31], which explains why the age group “ 26−50” was also identified as influential for treatment decision. For early invasive BC, patients under stages I, IIA, or IIB, ages between 51 and 75, and grade II were found to be more likely to receive BCSR. This result is in accordance to the National Comprehensive Cancer Network (NCCN) guidelines and very well demonstrates the usefulness of the Zoom-in heuristic in systematically identifying a group receiving a treatment.

From the branching in Detroit, we observed that a group of patients with DCIS and grade undetermined received BCS when the expected treatment is BCSR. We further iterated in the heuristic in search of variables that could explain this result. We were not able to find any further or meaningful explanations. We believe our conclusions were limited as the SEER data does not provide more specific categories of non-clinical variables such as “Insurance status” and “Willingness radiotherapy”. In “Insurance status”, SEER only presents the categories “Uninsured”, “Medicaid”, “Insured” or “Unknown”, and exclude categories like “Private” or “Medicare”. In the “Willingness radiotherapy” field, two options were considered: “Declined” and “Not declined”, but for this case the “Not declined” option was highly correlated to the proportion of patients receiving radiotherapy. We were not able to detect patients that did not receive radiotherapy but did not decline the treatment.

As mentioned before, patients’ risks of recurrence or death might be influenced by non-clinical and clinical variables. However, we believe that the identification of a more complete set of variables was limited by the information available in the SEER dataset. Non-clinical variables different from insurance status are not part of the dataset. Also, other important clinical variables such as treatments courses different from surgery and radiotherapy are not available.

The BN-Zoom-in approach can be easily implemented to identify and characterize the features of BC patients receiving other expected and unexpected treatments (e.g., chemotherapy, hormone therapy, immunotherapy, etc.). Medical facilities can also use this approach to test whether a specific treatment of a patient follows recommendations of the NCCN guidelines.

### Controlling Stage for BN-Zoom-in and logistic regression

Controlling is a very common technique for blocking the effect of influential variables that are already known. In addition, it allows a less noisy comparison between the BN-Zoom-in and the LR. In our results for Detroit, we documented the effect of BN-Zoom-in with the entire and controlled datasets. Both the entire and controlled datasets found common variables as influential. For example, LCIS histology is found in both cases but grade, age, tumor size, and primary site were not identified (See Table 8). These differences can be attributed to the variability from additional stages in the entire dataset. For Detroit, the unknown category of Tumor Size is significant when we controlled for Stage 0 but not in the entire dataset. To provide a rationale for this finding, we would need to go to the source of information (e.g., hospitals and patients’ charts) and check if there were mistakes in the way the information was collected or if there were healthcare deficiencies.

Also, to observe the effect of controlling in the BN and the LR, we ran different LR and BN-Zoom-in models using the datasets in each stage (i.e., 0, I, IIA, IIB) for Detroit. Table 8 shows the obtained results for each model. For stage 0, only LCIS histology was meaningful in both models. For stage I, IIA, and IIB, the BN-Zoom-in shows age 51−75 as an influential factor, while the LR did not find any observed significance. Structural differences in both models provide complementary conclusions that should be considered for decision making.

### BN-Zoom-in Generalization San Francisco-Oakland and Atlanta cases

#### San Francisco-Oakland

For the LR of San Francisco-Oakland, the variables grade undetermined, histology 8520 (LCIS), histology other, tumor size 6−11, 12−17, 18−23, 24−29, 30−35, 42−47, and degree/invasiveness F20 (i.e., invasive and situ components present) are significant in the model (see Additional file 1: Section 4). Table 10 presents the results of Accuracy, Sensitivity, and Specificity for both the BN and the LR model for the San Francisco-Oakland area. The prediction performance of the BN is lower for the Sensitivity parameter (see Additional file 1: Section 5). We believe this occurs because the LR identifies more variables as significant for the decision of treatment in San Francisco-Oakland. However, these factors do not improve the performance in the global Accuracy of the LR model when compared to the BN.

**Table 10:** Logistic regression and Bayesian network validation results (San Francisco-Oakland)

	Accuracy	Sensitivity	Specificity
BN	0.760	0.789	0.756
LR	0.756	0.938	0.744

#### Atlanta

For the LR of Atlanta, the variables year 2011, year 2010, grade undetermined, histology 8520, and histology 8501 are significant in the model (see Additional file 1: Section 6). For Atlanta, the year was identified for both models as an influential factor in treatment decision. This finding is related to a reduction of radiotherapy receipt in these years for the Atlanta area. Table 11 presents the results of Accuracy, Sensitivity, and Specificity for both the BN and the LR model for the Atlanta area. The prediction performance of the BN for Atlanta is also lower for the Sensitivity parameter as in San Francisco-Oakland (see Additional file 1: Section 7). But, similarly these factors do not improve the performance in the global Accuracy of the LR model when compared to the BN.

**Table 11:** Logistic regression and Bayesian network validation results (Atlanta)

	Accuracy	Sensitivity	Specificity
BN	0.773	0.630	0.785
LR	0.790	0.912	0.785

## Conclusions

The Zoom-in heuristic identifies the most common conditions and variables for which a patient might receive a target treatment. The BN-Zoom-in approach was useful in analyzing general treatment recommendations for BC patients in the metropolitan Detroit, San Francisco-Oakland, and metropolitan Atlanta with the use of the SEER dataset.

For Detroit, patients under stage 0, grade undetermined, with histology of lobular carcinoma in situ (LCIS) (i.e., 8520), and with age between 26 and 50 are declared as the group more likely to receive BCS for in situ BC. Similarly, patients under stages I, IIA, IIB, with age between 51 and 75, and with grade II are declared as the group more likely to receive BCSR for early invasive BC. For San Francisco-Oakland, we found that patients in stage 0, grade undetermined, and age >75 are more likely to receive BCS. For stages I, IIA, IIB patients with age >75 are more likely to receive BCS. For Atlanta, patients in stage 0, grade undetermined, year 2011, and primary site C509 (i.e., entire breast) are more likely to receive BCS. For stages I, IIA, IIB patients in year 2011, and grade III are more likely to receive BCS.

Our results show that most of the patients are receiving treatment in agreement with the NCCN guidelines and medical consensus. Through a branching analysis in the heuristic, we were able to identify a subset of the population in Detroit with DCIS for which BCS was recommended, but potential reasons for this treatment are still unknown. To validate and assess the prediction power of the BN, logistic regression models (LR) were built with the same information provided by the SEER. The global prediction power of the BN is statistically similar to the LR. Structural differences in both models provide complementary conclusions that should be considered for decision-making.

Our approach shows a promising avenue for exploring treatment recommendations in a systematic and comprehensive approach. Further research should explore the methodology with datasets in which more information about demographic and socioeconomic features are present. Also, information about genetic features, comorbidities, and other treatments administered to the patient can be incorporated to explore further interactions and improve the significance of the results.

## Additional file

Additional file 1 Section 1. Further explanation of the categories for the variables in Table 1 (please refer to the article); Details of the Bayesian Network learning; Bayesian Network analysis - Graphs Probabilities values for all the variables in each step of the BN-Zoom-in approach. Section 2. Output results from the logistic regression model using SEER data from Detroit. Section 3. Validation results from Bayesian Network and Logistic regression models using SEER data from Detroit. Section 4. Output results from the logistic regression model using SEER data from San Francisco - Oakland. Section 5. Validation results from Bayesian Network and Logistic regression models using SEER data from San Francisco - Oakland. Section 6. Output results from the logistic regression model using SEER data from Atlanta. Section 7. Validation results from Bayesian Network and Logistic regression models using SEER data from Atlanta. (PDF 1.45 MB)

## Abbreviations

AJCC: American Joint Committee on Cancer
BC: Breast cancer
BCS: Breast-conservation surgery without radiotherapy
BCSR: Breast-conservation surgery with radiotherapy
BN: Bayesian network
BN-Zoom-in: Bayesian network and Zoom-in heuristic
CPTs: Conditional probability tables
DAG: Direct acyclic graph
DCIS: Ductal carcinoma in situ
LCIS: Lobular carcinoma in situ
LR: Logistic regression
NCCN: National Comprehensive Cancer Network
SEER: Surveillance, Epidemiology, and End Results
