# Supplementary material for: A Bayesian network and heuristic approach for systematic characterization of radiotherapy receipt after breast-conservation surgery
Source: BMC Med Inform Decis Mak. 2017 Jun 28;17:93. doi: 10.1186/s12911-017-0479-4 (PMC5490206; doi:10.1186/s12911-017-0479-4)
Supplement: Additional file 1 — Section 1. Further explanation of the categories for the variables in Table 1 (please refer to the article); Details of the Bayesian Network learning; Bayesian Network analysis - Graphs Probabilities values for all the variables in each step of the BN-Zoom-in approach. Section 2. Output results from the logistic regression model using SEER data from Detroit. Section 3. Validation results from Bayesian Network and Logistic regression models using SEER data from Detroit. Section 4. Output results from the logistic regression model using SEER data from San Francisco - Oakland. Section 5. Validation results from Bayesian Network and Logistic regression models using SEER data from San Francisco - Oakland. Section 6. Output results from the logistic regression model using SEER data from Atlanta. Section 7. Validation results from Bayesian Network and Logistic regression models using SEER data from Atlanta. (PDF 1.45 MB) [file 12911_2017_479_MOESM1_ESM.pdf]

# Supplementary

## Section 1

### Categories per Variable

### Primary Site

| ID | |
|---|---|
| C500 | Nipple (areolar) |
| C501 | Central portion of breast (subareolar) area extending 1 cm around areolar complex |
| C502 | Upper inner quadrant (UIQ) of breast |
| C503 | Lower inner quadrant (LIQ) of breast |
| C504 | Upper outer quadrant (UOQ) of breast |
| C505 | Lower outer quadrant (LOQ) of breast |
| C506 | Axillary tail of breast |
| C508 | Overlapping lesion of breast |
| C509 | Breast, NOS |

### Tumor Size

| Range | | ID |
|---|---|---|
| 0 | 5 | **S0** |
| 6 | 11 | **S1** |
| 12 | 17 | **S2** |
| 18 | 23 | **S3** |
| 24 | 29 | **S4** |
| 30 | 35 | **S5** |
| 36 | 41 | **S6** |
| 42 | 47 | **S7** |
| 48 | 53 | **S8** |
| 54 | 59 | **S9** |
| 60 | 65 | **S10** |
| 66 | 71 | **S11** |
| 72 | 77 | **S12** |
| 78 | 83 | **S13** |
| 84 | 89 | **S14** |
| 90 | 95 | **S15** |
| 96 | 101 | **S16** |
| >=102 | | **S17** |
| Unknown | Not Applicable | **S21** |

## Histology

| ID | HISTO3V | Name Category | Name Specific |
|---|---|---|---|
| H1 | **8201** | ADENOID CYSTIC & CRIBRIFORM CA. | Cribriform carcinoma in situ |
| H2 | **8211** | ADENOCA. IN ADENOMA. POLYP | Tubular adenocarcinoma |
| H3 | **8230** | SOLID CARCINOMA, NOS | Duct carcinoma in situ, solid type |
| H4 | **8480** | MUCINOUS ADENOCARCINOMA | Mucinous adenocarcinoma |
| H5 | **8500** | DUCT CARCINOMA | Intraductal carcinoma, noninfiltrating, NOS |
| H6 | **8501** | DUCT CARCINOMA | Comedocarcinoma, non-infiltrating |
| H7 | **8503** | DUCT CARCINOMA | Noninfiltrating intraductal papillary adenocarcinoma |
| H8 | **8507** | DUCT CARCINOMA | Intraductal micropapillary carcinoma |
| H9 | **8520** | LOBULAR AND OTHER DUCTAL CA. | Lobular carcinoma in situ |
| H10 | **8522** | LOBULAR AND OTHER DUCTAL CA. | Intraductal and lobular in situ carcinoma |
| H11 | **8523** | LOBULAR AND OTHER DUCTAL CA. | Infiltr. duct mixed with other types of carcinoma, in situ |

## Grade

| ID | Description |
|---|---|
| G1 | Grade I; grade i; grade 1; well differentiated; differentiated, NOS |
| G2 | Grade II; grade ii; grade 2; moderately differentiated; moderately differentiated; intermediate differentiation |
| G3 | Grade III; grade iii; grade 3; poorly differentiated; differentiated |
| G4 | Grade IV; grade iv; grade 4; undifferentiated; anaplastic |
| G9 | cell type not determined, not stated or not applicable |

## Behavior

| ID | Description |
|---|---|
| B2 | In situ |
| B3 | Invasive |

## Age

| ID | |
|---|---|
| R1 | <=25 |
| R2 | 25> and <=50 |
| R3 | 50> and <=75 |
| R4 | >75 |

**Marital Status**

| ID | Description |
|---|---|
| M1 | Single (never married) |
| M2 | Married (including common law) |
| M3 | Separated |
| M4 | Divorced |
| M5 | Widowed |
| M6 | Unmarried or domestic partner (same sex or opposite sex or unregistered) |
| M9 | Unknown |

**Insurance**

| ID | Description |
|---|---|
| I1 | Uninsured |
| I2 | Any Medicaid |
| I3 | Insured |
| I9 | Not available |

**Race**

| ID | Description |
|---|---|
| W | White |
| B | Black |
| O | Other |
| U | Unknown |

**Willingness Radiotherapy**

| ID | Description |
|---|---|
| NR1 | Not Declined |
| NR2 | Declined |

**Bayesian Network Learning**

**Outcome Hill-Climbing**

| from | to | strength | direction |
|---|---|---|---|
| M | R | 1 | 0.536 |
| M | IN | 0.983 | 0.504578 |
| M | A | 1 | 0.499 |
| P | B | 0.996 | 0.305723 |
| G | W | 0.242 | 0.997934 |
| G | T | 0.955 | 0.973822 |
| G | H | 1 | 0.952 |
| I | S | 1 | 0.51 |
| I | B | 1 | 0.523 |
| I | A | 0.96 | 0.495313 |
| S | G | 0.436 | 0.90367 |
| S | I | 1 | 0.49 |
| S | B | 0.544 | 0.492647 |
| S | TS | 1 | 0.556 |
| W | T | 1 | 0.109 |
| W | H | 0.117 | 0.324786 |
| B | P | 0.996 | 0.694277 |
| B | G | 0.588 | 0.97534 |
| B | I | 1 | 0.477 |
| B | S | 0.544 | 0.507353 |
| B | A | 0.311 | 0.482315 |
| B | T | 0.949 | 0.988936 |
| B | H | 0.976 | 0.993852 |
| R | M | 1 | 0.464 |
| R | G | 0.259 | 1 |
| IN | M | 0.983 | 0.495422 |
| A | M | 1 | 0.501 |
| A | I | 0.96 | 0.504688 |
| A | W | 0.603 | 0.995025 |
| A | B | 0.311 | 0.517685 |
| A | T | 1 | 0.9955 |
| T | Y | 0.348 | 1 |
| T | P | 0.25 | 0.992 |
| T | W | 1 | 0.891 |
| T | H | 0.572 | 0.563811 |
| T | TS | 0.122 | 0.729508 |
| H | W | 0.117 | 0.675214 |

| | | | |
|---|---|---|---|
| H | T | 0.572 | 0.436189 |
| TS | G | 0.564 | 0.988475 |
| TS | S | 1 | 0.444 |
| TS | T | 0.122 | 0.270492 |

**BN learning conditions**

```
$whitelist
NULL

$blacklist
NULL

$test
[1] "none"

$ntests
[1] 0

$algo
[1] "averaged"

$args
$args$threshold
[1] 0.25
```

**BN Nodes**

```
$M
$M$mb
[1] "R"  "IN" "A"

$M$nbr
[1] "R"  "IN" "A"

$M$parents
[1] "A"

$M$children
[1] "R"  "IN"


$Y
$Y$mb
[1] "T"

$Y$nbr
[1] "T"

$Y$parents
[1] "T"
```

```
$Y$children
character(0)


$P
$P$mb
[1] "B"

$P$nbr
[1] "B"

$P$parents
[1] "B"

$P$children
character(0)


$G
$G$mb
[1] "S"  "B"  "R"  "A"  "T"  "H"  "TS"

$G$nbr
[1] "S"  "B"  "R"  "T"  "H"  "TS"

$G$parents
[1] "S"  "B"  "R"  "TS"

$G$children
[1] "T" "H"


$I
$I$mb
[1] "S" "B" "A"

$I$nbr
[1] "S" "B" "A"

$I$parents
[1] "A"

$I$children
[1] "S" "B"


$S
$S$mb
[1] "G"  "I"  "B"  "R"  "TS"

$S$nbr
[1] "G"  "I"  "B"  "TS"

$S$parents
[1] "I" "B"

$S$children
```

```
[1] "G"  "TS"


$W
$W$mb
[1] "A" "T"

$W$nbr
[1] "A" "T"

$W$parents
[1] "A" "T"

$W$children
character(0)


$B
$B$mb
[1] "P"  "G"  "I"  "S"  "R"  "A"  "T"  "H"  "TS"

$B$nbr
[1] "P" "G" "I" "S" "A" "T" "H"

$B$parents
[1] "I" "A"

$B$children
[1] "P" "G" "S" "T" "H"


$R
$R$mb
[1] "M"  "G"  "S"  "B"  "TS"

$R$nbr
[1] "M" "G"

$R$parents
[1] "M"

$R$children
[1] "G"


$IN
$IN$mb
[1] "M"

$IN$nbr
[1] "M"

$IN$parents
[1] "M"

$IN$children
character(0)
```

```
$A
$A$mb
[1] "M" "G" "I" "W" "B" "T"

$A$nbr
[1] "M" "I" "W" "B" "T"

$A$parents
character(0)

$A$children
[1] "M" "I" "W" "B" "T"


$T
$T$mb
[1] "Y" "G" "W" "B" "A" "H"

$T$nbr
[1] "Y" "G" "W" "B" "A" "H"

$T$parents
[1] "G" "B" "A"

$T$children
[1] "Y" "W" "H"


$H
$H$mb
[1] "G" "B" "T"

$H$nbr
[1] "G" "B" "T"

$H$parents
[1] "G" "B" "T"

$H$children
character(0)


$TS
$TS$mb
[1] "G" "S" "B" "R"

$TS$nbr
[1] "G" "S"

$TS$parents
[1] "S"

$TS$children
[1] "G"
```

### BN Arcs

```
      from to  
 [1,] "M"  "R" 
 [2,] "M"  "IN"
 [3,] "G"  "T" 
 [4,] "G"  "H" 
 [5,] "I"  "S" 
 [6,] "I"  "B" 
 [7,] "S"  "G" 
 [8,] "S"  "TS"
 [9,] "B"  "P" 
[10,] "B"  "G" 
[11,] "B"  "S" 
[12,] "B"  "T" 
[13,] "B"  "H" 
[14,] "R"  "G" 
[15,] "A"  "M" 
[16,] "A"  "I" 
[17,] "A"  "W" 
[18,] "A"  "B" 
[19,] "A"  "T" 
[20,] "T"  "Y" 
[21,] "T"  "W" 
[22,] "T"  "H" 
[23,] "TS" "G" 
```

## BN Analysis

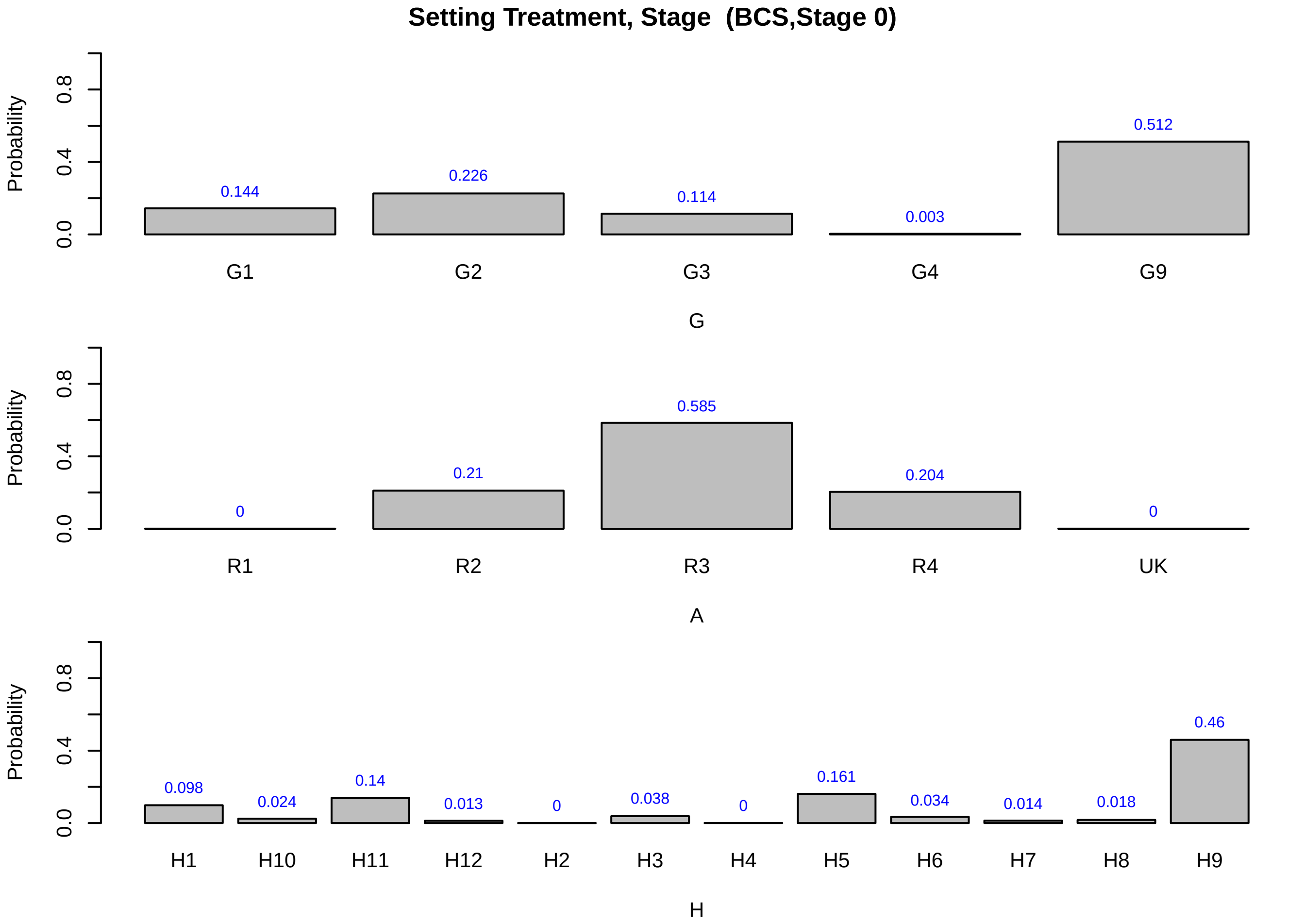
Setting Treatment, Stage (BCS,Stage 0)
Probability
0.0
0.4
0.8
0.144
0.226
0.114
0.003
0.512
G1
G2
G3
G4
G9
G
0
0.21
0.585
0.204
0
R1
R2
R3
R4
UK
A
0.098
0.024
0.14
0.013
0
0.038
0
0.161
0.034
0.014
0.018
0.46
H1
H10
H11
H12
H2
H3
H4
H5
H6
H7
H8
H9
H

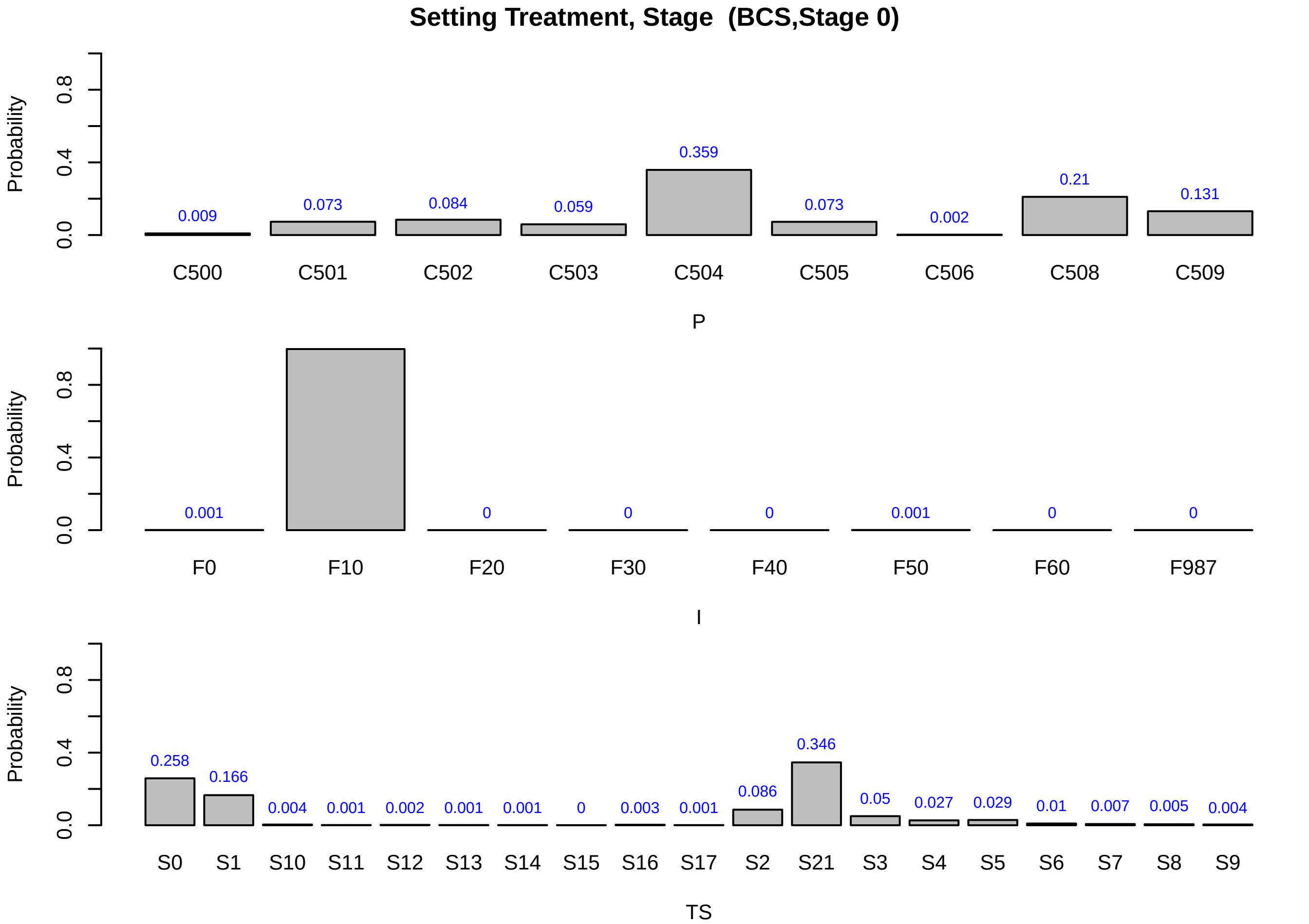

Setting Treatment, Stage (BCS,Stage 0)
Probability
0.0
0.4
0.8
0.009
0.073
0.084
0.059
0.359
0.073
0.002
0.21
0.131
C500
C501
C502
C503
C504
C505
C506
C508
C509
P
Probability
0.0
0.4
0.8
0.001
0
0
0
0.001
0
0
F0
F10
F20
F30
F40
F50
F60
F987
I
Probability
0.0
0.4
0.8
0.258
0.166
0.004
0.001
0.002
0.001
0.001
0
0.003
0.001
0.086
0.346
0.05
0.027
0.029
0.01
0.007
0.005
0.004
S0
S1
S10
S11
S12
S13
S14
S15
S16
S17
S2
S21
S3
S4
S5
S6
S7
S8
S9
TS

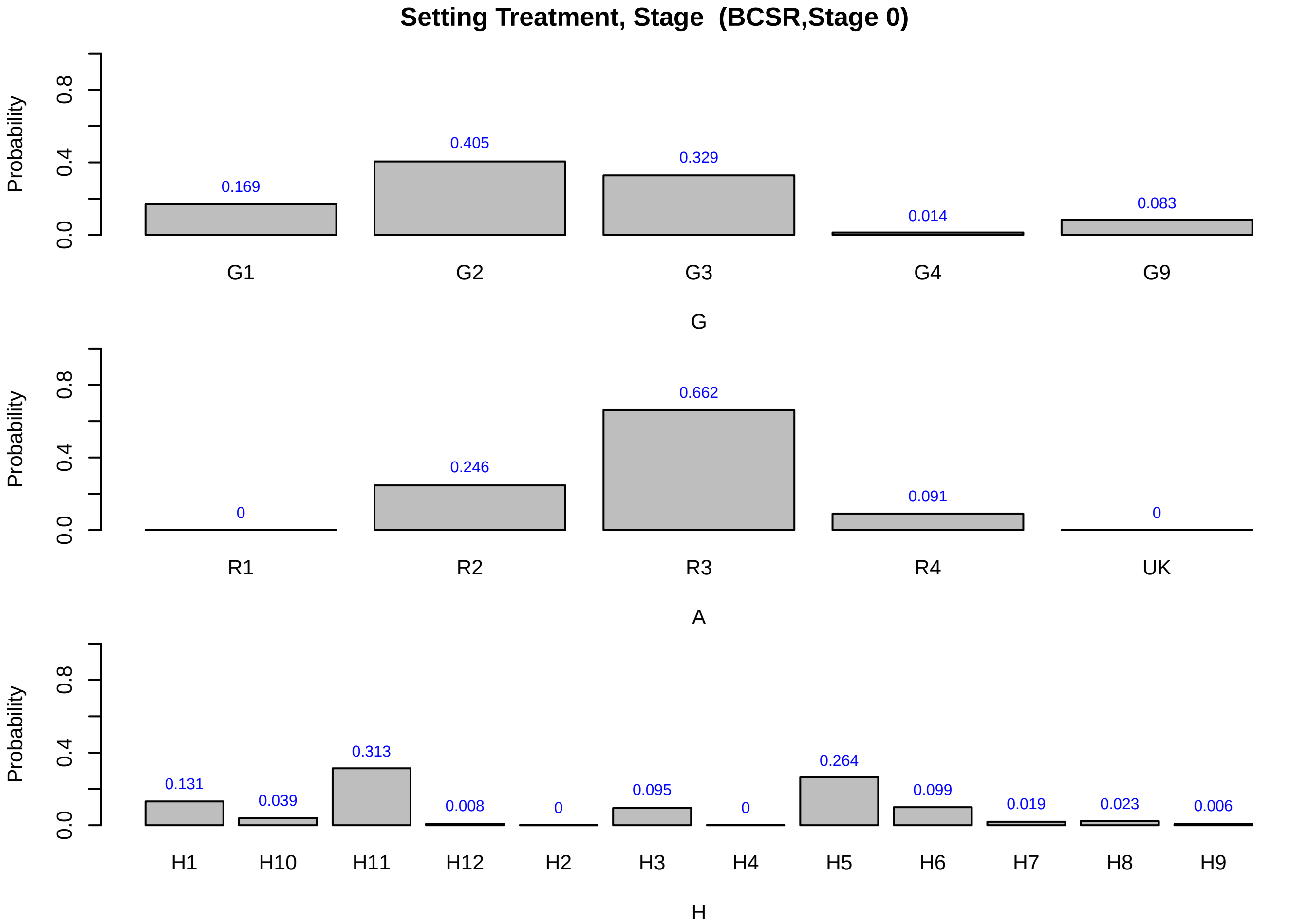
Setting Treatment, Stage (BCSR,Stage 0)
Probability
0.0
0.4
0.8
0.169
0.405
0.329
0.014
0.083
G1
G2
G3
G4
G9
G
Probability
0.0
0.4
0.8
0
0.246
0.662
0.091
0
R1
R2
R3
R4
UK
A
Probability
0.0
0.4
0.8
0.131
0.039
0.313
0.008
0
0.095
0
0.264
0.099
0.019
0.023
0.006
H1
H10
H11
H12
H2
H3
H4
H5
H6
H7
H8
H9
H

**Setting Treatment, Stage (BCSR,Stage 0)**

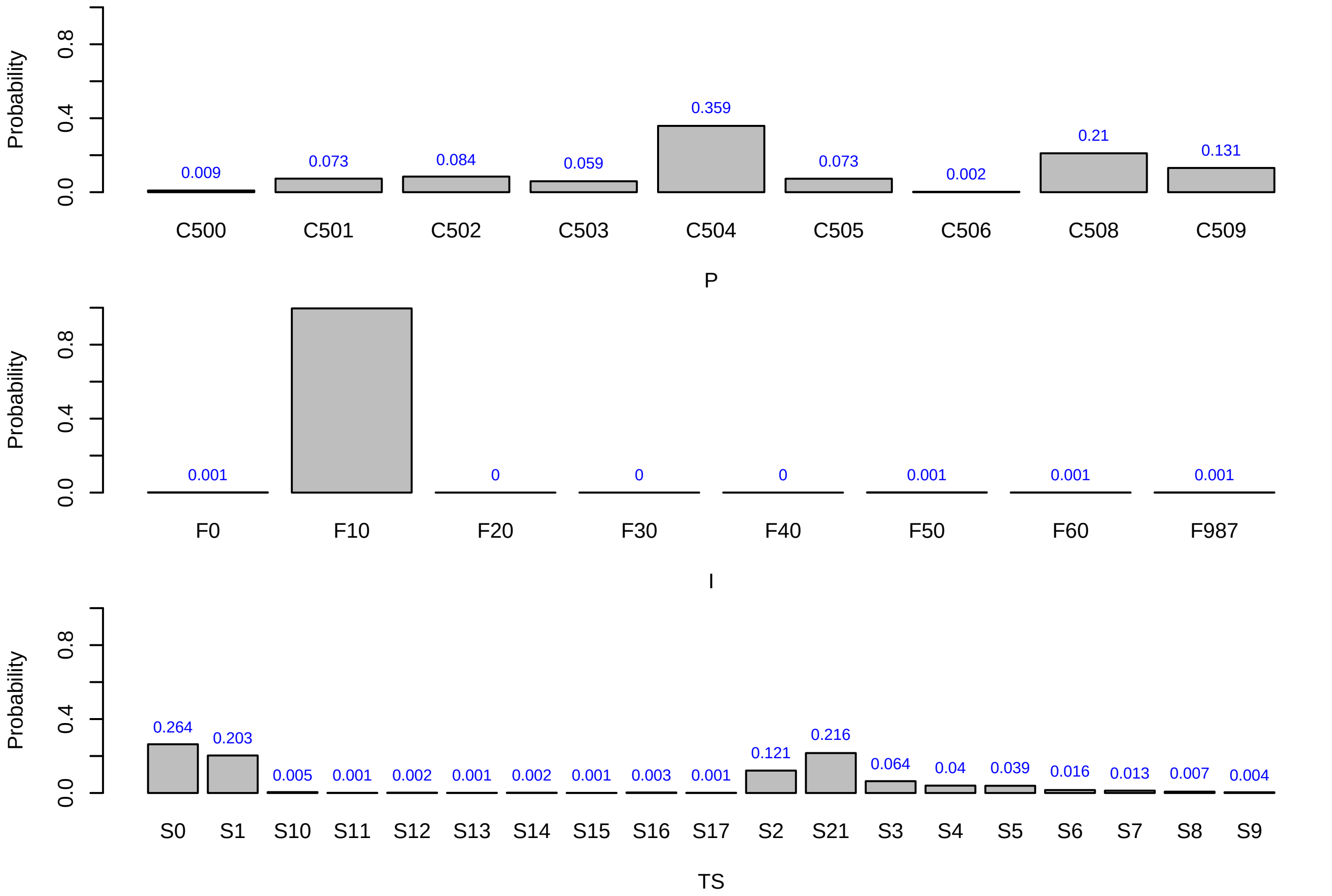

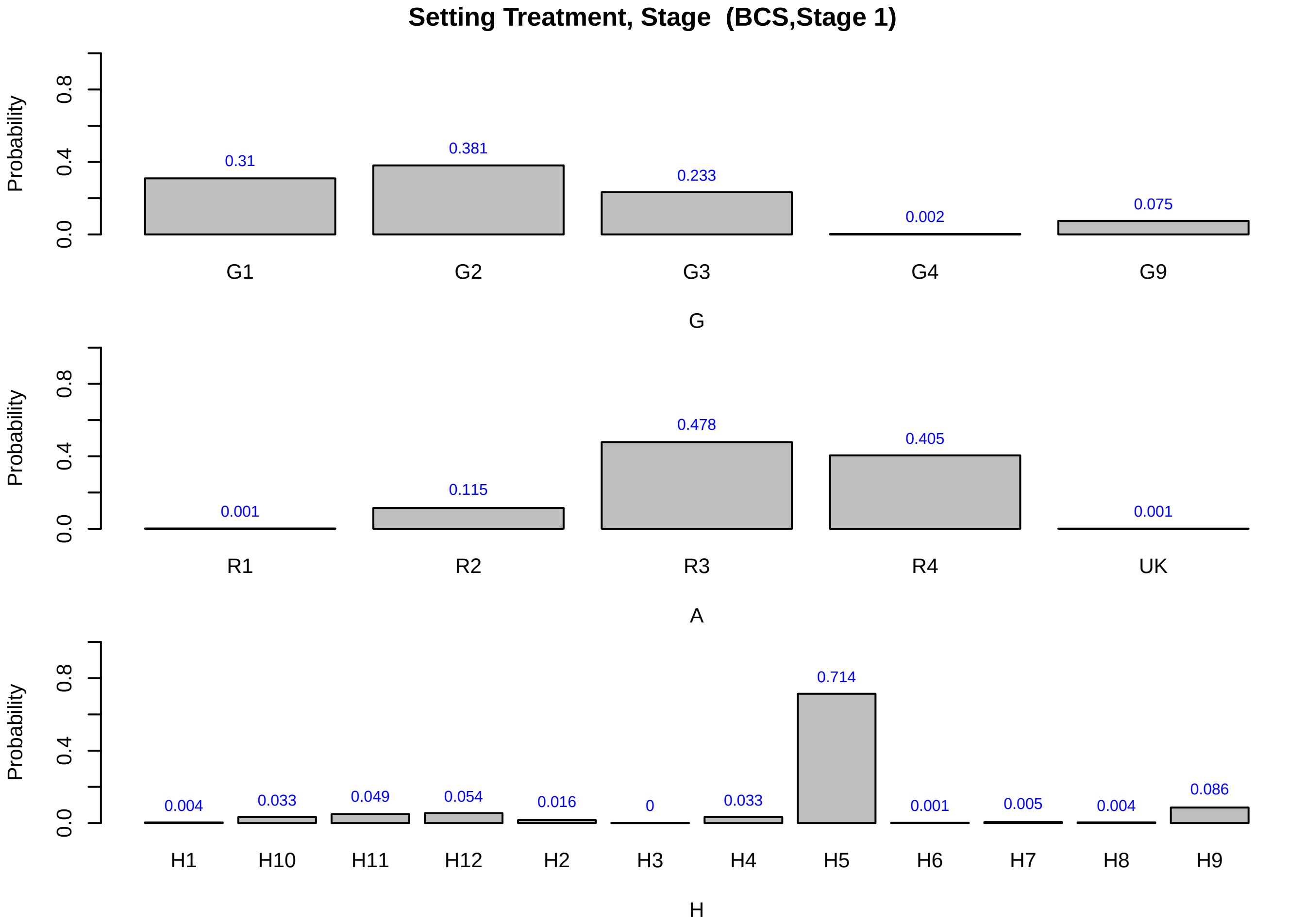
Setting Treatment, Stage (BCS,Stage 1)
Probability
0.0
0.4
0.8
0.31
0.381
0.233
0.002
0.075
G1
G2
G3
G4
G9
G
0.001
0.115
0.478
0.405
0.001
R1
R2
R3
R4
UK
A
0.004
0.033
0.049
0.054
0.016
0
0.033
0.714
0.001
0.005
0.004
0.086
H1
H10
H11
H12
H2
H3
H4
H5
H6
H7
H8
H9
H

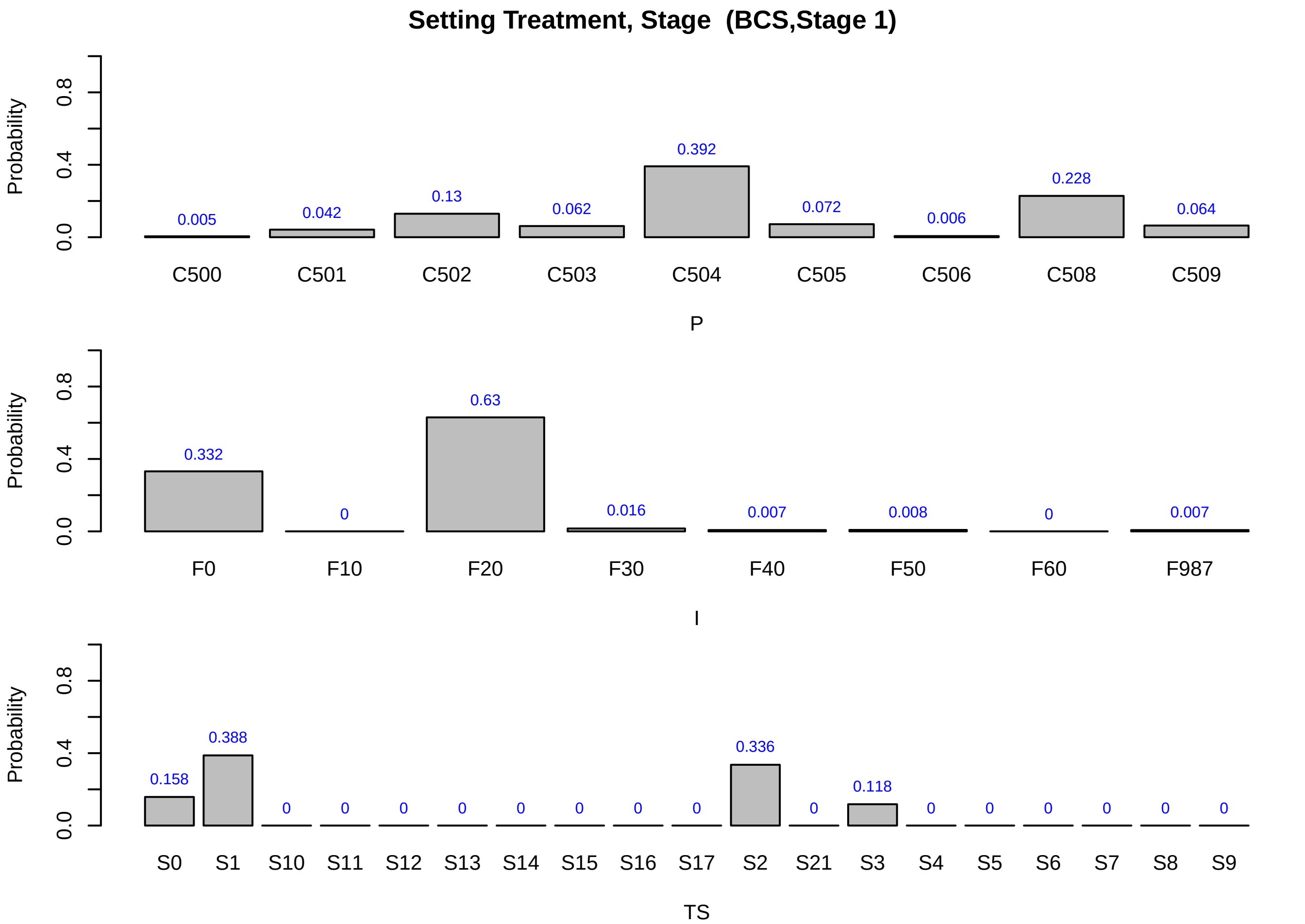
Setting Treatment, Stage (BCS,Stage 1)
Probability
0.0
0.4
0.8
0.005
0.042
0.13
0.062
0.392
0.072
0.006
0.228
0.064
C500
C501
C502
C503
C504
C505
C506
C508
C509
P
0.332
0
0.63
0.016
0.007
0.008
0
0.007
F0
F10
F20
F30
F40
F50
F60
F987
I
0.158
0.388
0
0
0
0
0
0
0
0
0.336
0
0.118
0
0
0
0
0
0
S0
S1
S10
S11
S12
S13
S14
S15
S16
S17
S2
S21
S3
S4
S5
S6
S7
S8
S9
TS

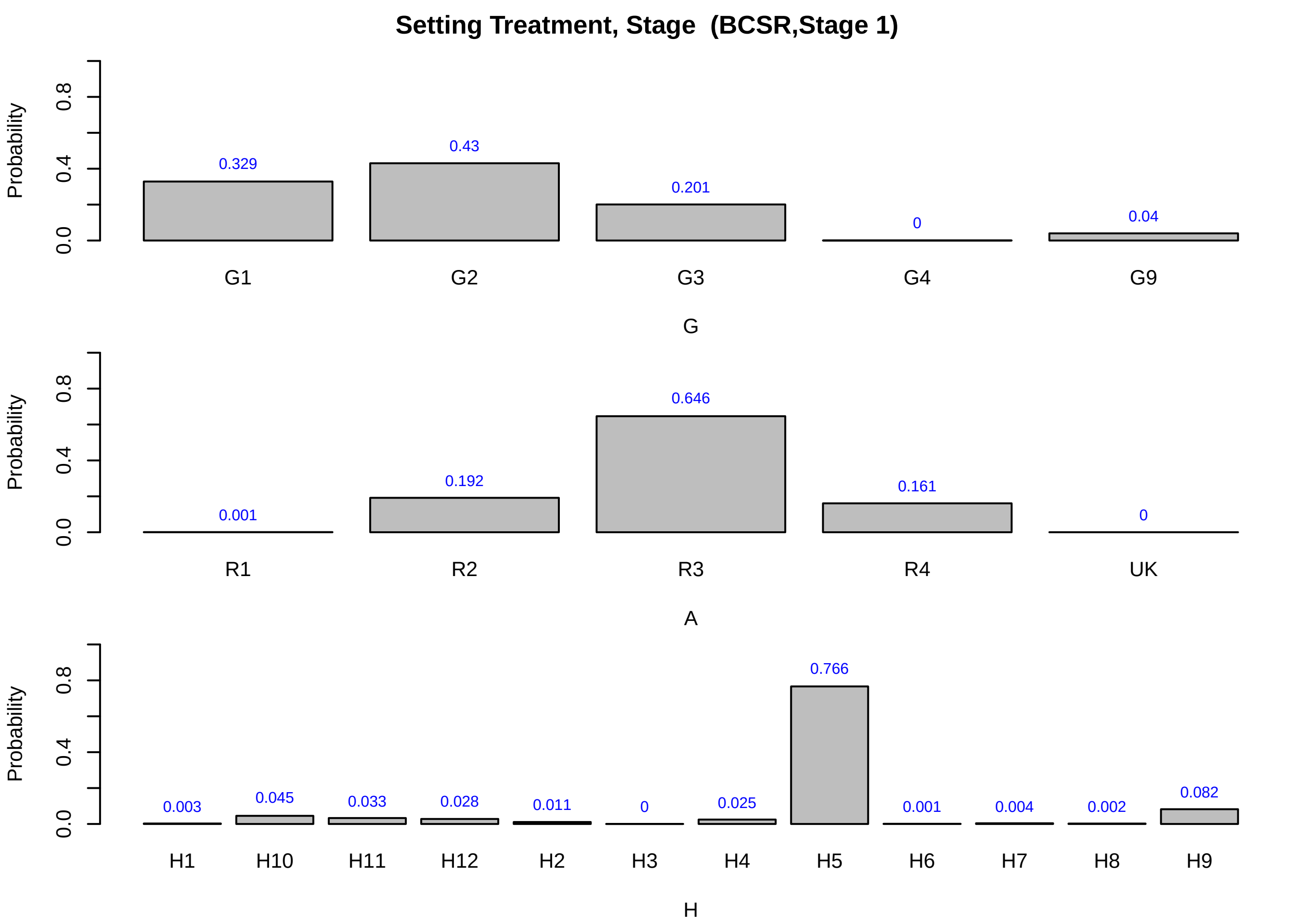
Setting Treatment, Stage (BCSR,Stage 1)
Probability
0.0
0.4
0.8
0.329
0.43
0.201
0
0.04
G1
G2
G3
G4
G9
G
Probability
0.0
0.4
0.8
0.001
0.192
0.646
0.161
0
R1
R2
R3
R4
UK
A
Probability
0.0
0.4
0.8
0.003
0.045
0.033
0.028
0.011
0
0.025
0.766
0.001
0.004
0.002
0.082
H1
H10
H11
H12
H2
H3
H4
H5
H6
H7
H8
H9
H

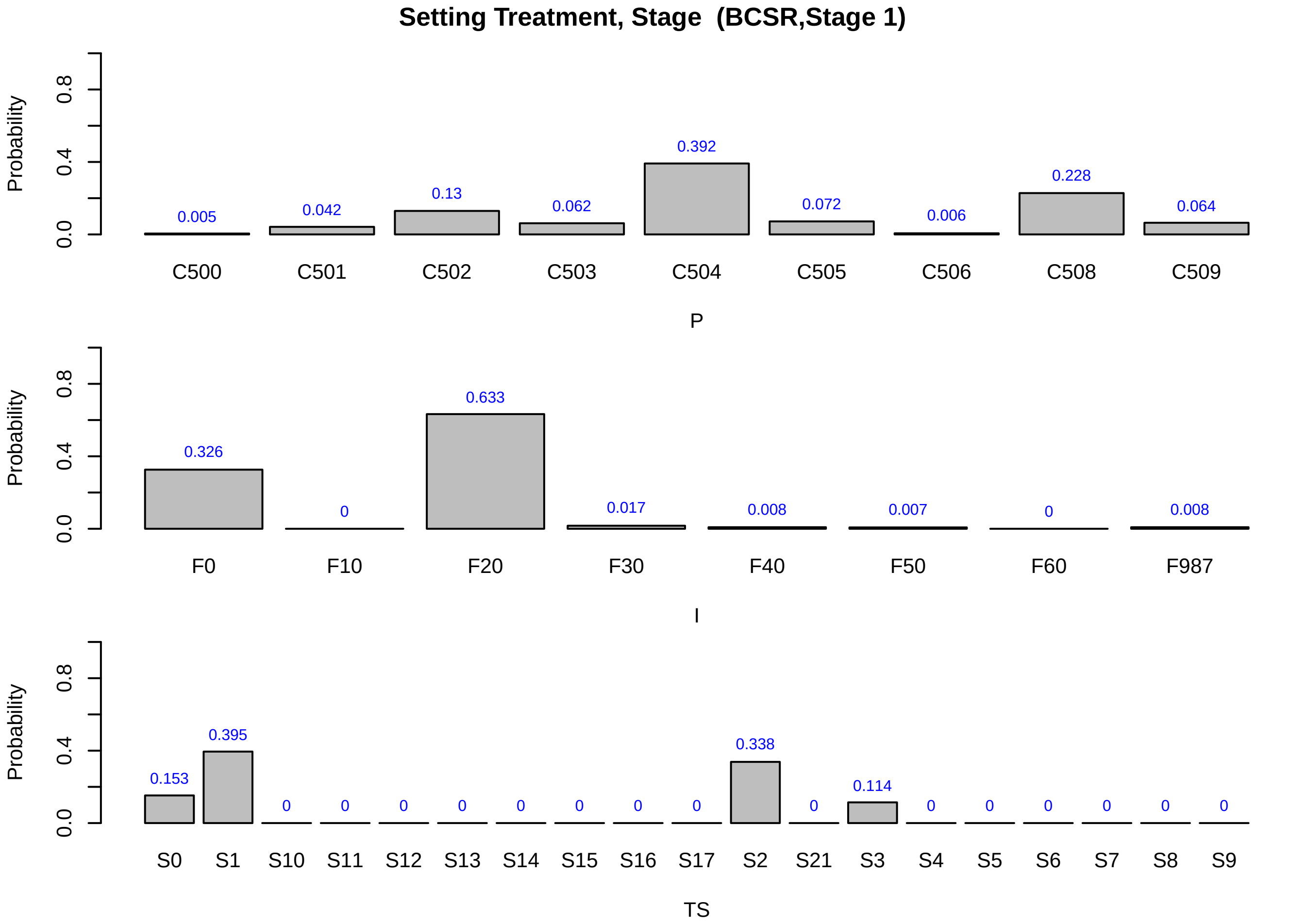

Setting Treatment, Stage (BCSR,Stage 1)
Probability
0.0
0.4
0.8
0.005
0.042
0.13
0.062
0.392
0.072
0.006
0.228
0.064
C500
C501
C502
C503
C504
C505
C506
C508
C509
P
Probability
0.0
0.4
0.8
0.326
0
0.633
0.017
0.008
0.007
0
0.008
F0
F10
F20
F30
F40
F50
F60
F987
I
Probability
0.0
0.4
0.8
0.153
0.395
0
0
0
0
0
0
0
0
0.338
0
0.114
0
0
0
0
0
0
S0
S1
S10
S11
S12
S13
S14
S15
S16
S17
S2
S21
S3
S4
S5
S6
S7
S8
S9
TS

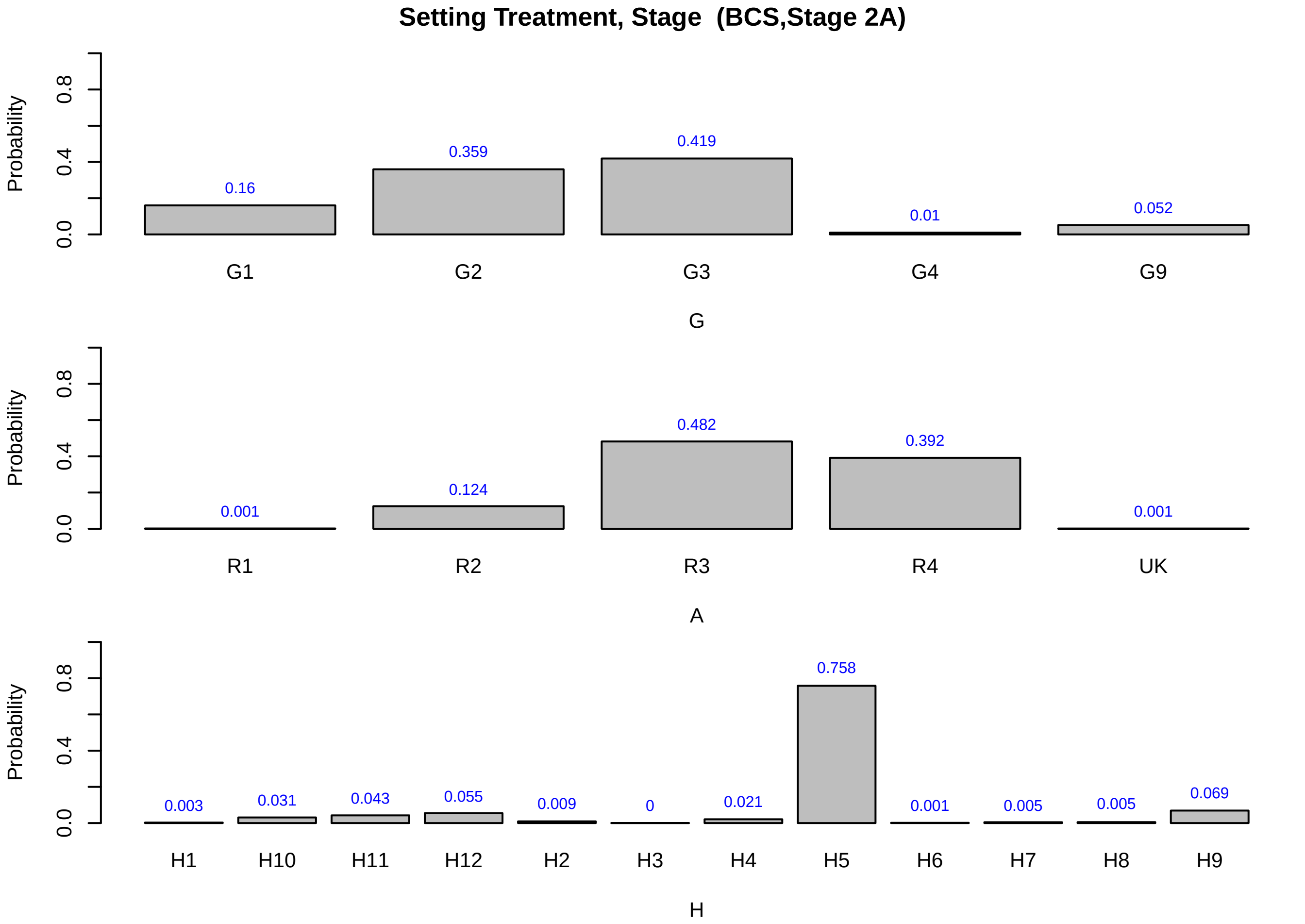
Setting Treatment, Stage (BCS,Stage 2A)
Probability
0.0
0.4
0.8
0.16
0.359
0.419
0.01
0.052
G1
G2
G3
G4
G9
G
Probability
0.0
0.4
0.8
0.001
0.124
0.482
0.392
0.001
R1
R2
R3
R4
UK
A
Probability
0.0
0.4
0.8
0.003
0.031
0.043
0.055
0.009
0
0.021
0.758
0.001
0.005
0.005
0.069
H1
H10
H11
H12
H2
H3
H4
H5
H6
H7
H8
H9
H

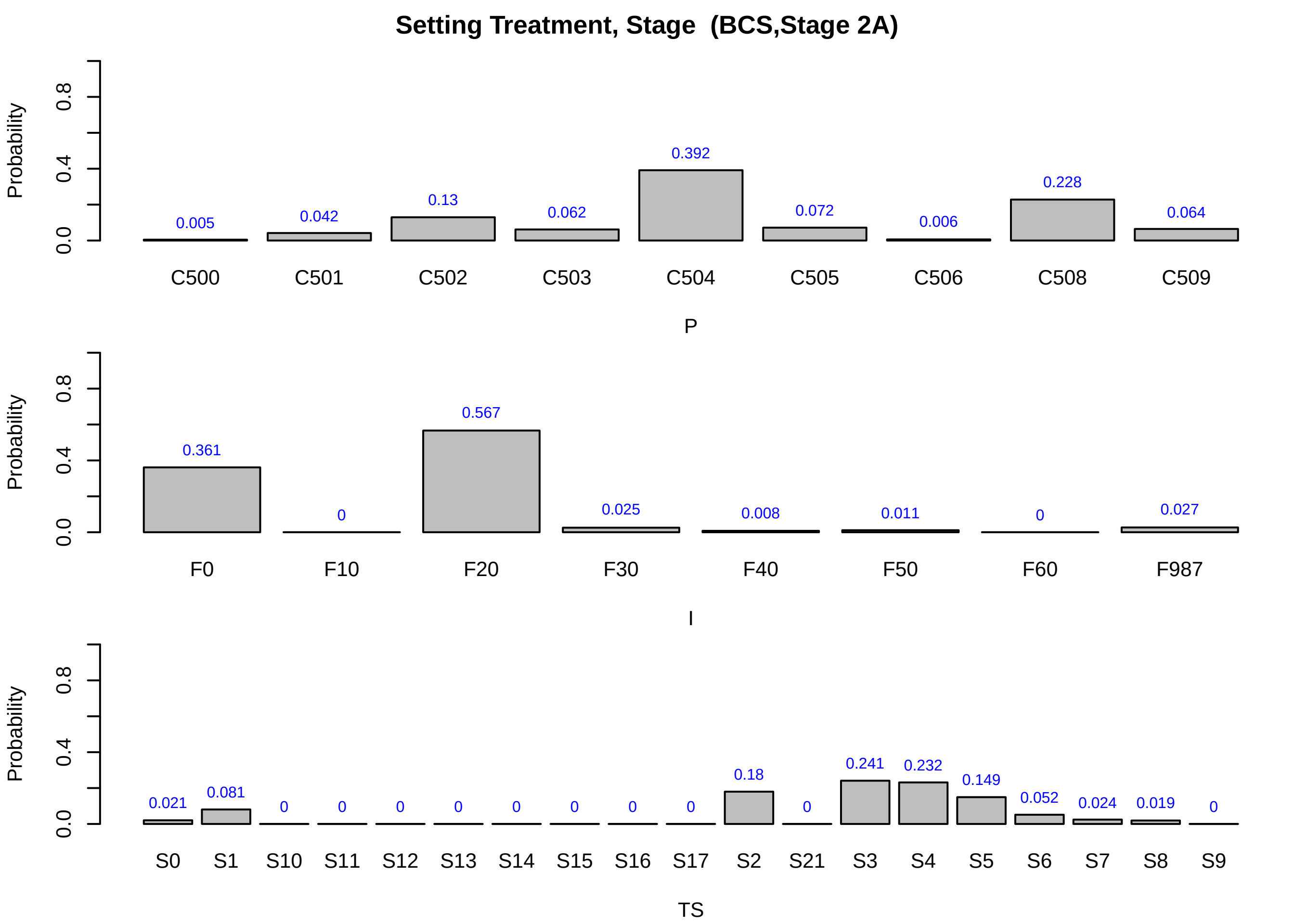

Setting Treatment, Stage (BCS,Stage 2A)
Probability
0.0
0.4
0.8
0.005
0.042
0.13
0.062
0.392
0.072
0.006
0.228
0.064
C500
C501
C502
C503
C504
C505
C506
C508
C509
P
Probability
0.0
0.4
0.8
0.361
0
0.567
0.025
0.008
0.011
0
0.027
F0
F10
F20
F30
F40
F50
F60
F987
I
Probability
0.0
0.4
0.8
0.021
0.081
0
0
0
0
0
0
0
0
0.18
0
0.241
0.232
0.149
0.052
0.024
0.019
0
S0
S1
S10
S11
S12
S13
S14
S15
S16
S17
S2
S21
S3
S4
S5
S6
S7
S8
S9
TS

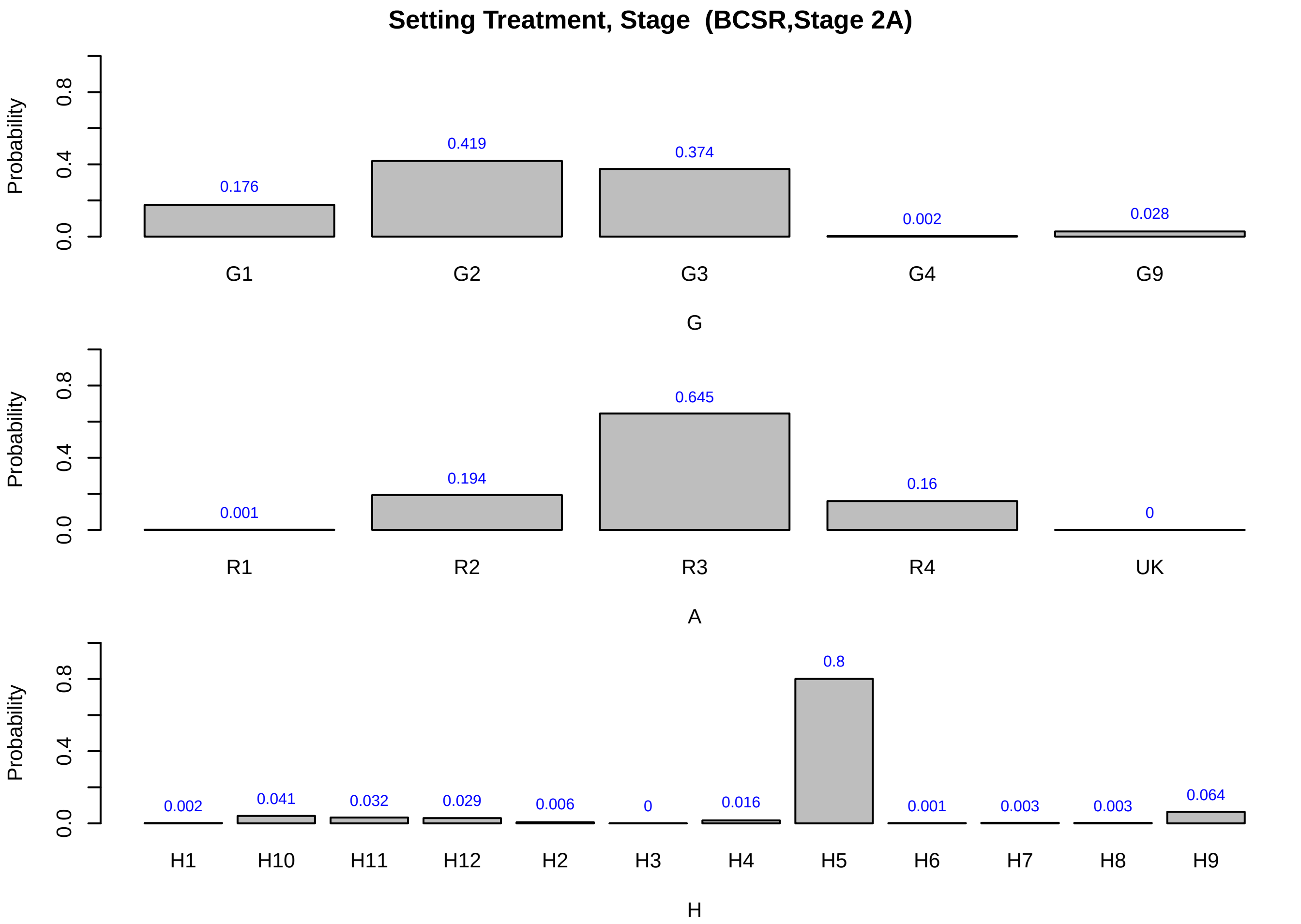

Setting Treatment, Stage (BCSR,Stage 2A)
Probability
0.0
0.4
0.8
0.176
0.419
0.374
0.002
0.028
G1
G2
G3
G4
G9
G
Probability
0.0
0.4
0.8
0.001
0.194
0.645
0.16
0
R1
R2
R3
R4
UK
A
Probability
0.0
0.4
0.8
0.002
0.041
0.032
0.029
0.006
0
0.016
0.8
0.001
0.003
0.003
0.064
H1
H10
H11
H12
H2
H3
H4
H5
H6
H7
H8
H9
H

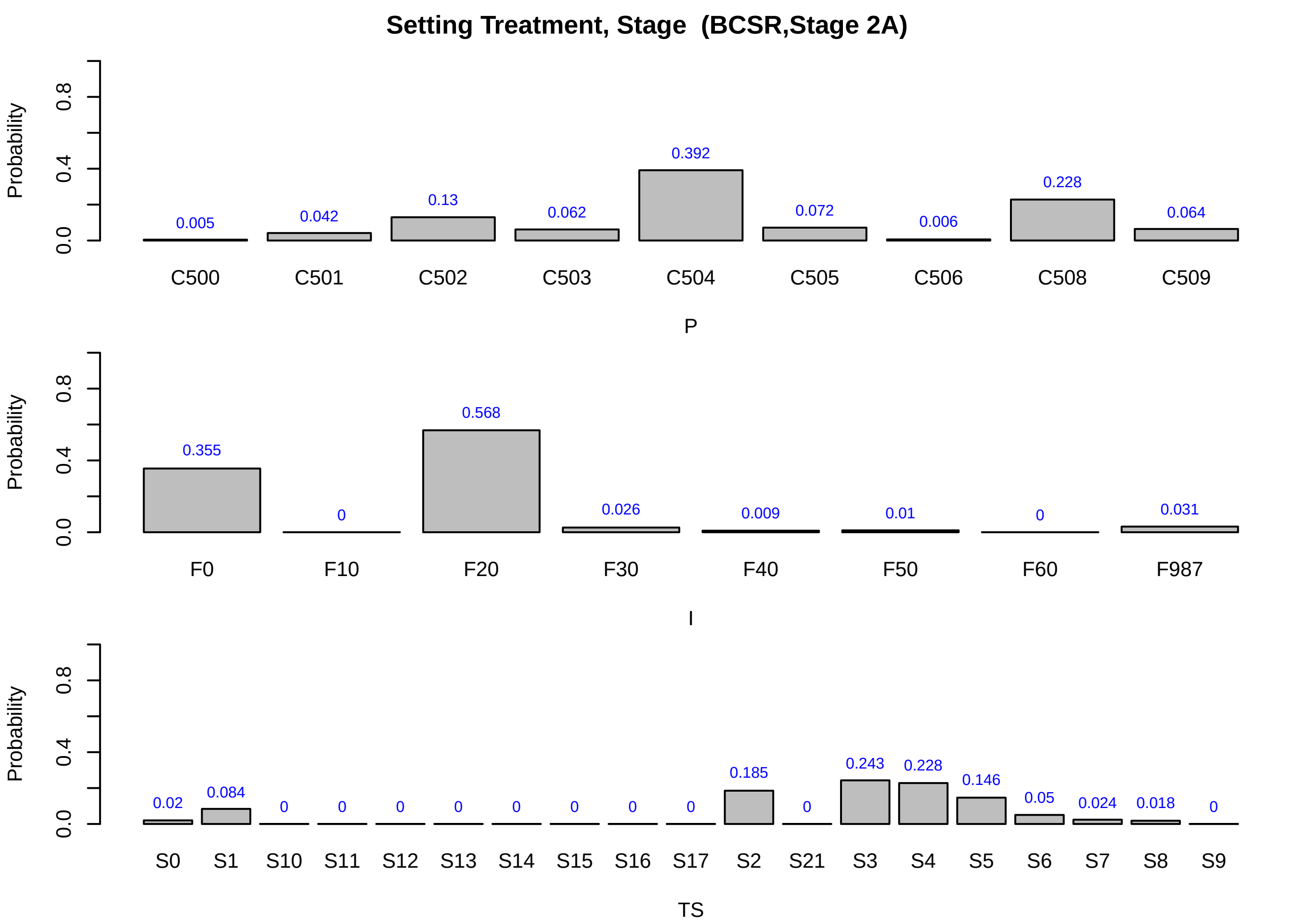

Setting Treatment, Stage (BCSR,Stage 2A)
Probability
0.0
0.4
0.8
0.005
0.042
0.13
0.062
0.392
0.072
0.006
0.228
0.064
C500
C501
C502
C503
C504
C505
C506
C508
C509
P
Probability
0.355
0
0.568
0.026
0.009
0.01
0
0.031
F0
F10
F20
F30
F40
F50
F60
F987
I
Probability
0.02
0.084
0
0
0
0
0
0
0
0
0.185
0
0.243
0.228
0.146
0.05
0.024
0.018
0
S0
S1
S10
S11
S12
S13
S14
S15
S16
S17
S2
S21
S3
S4
S5
S6
S7
S8
S9
TS

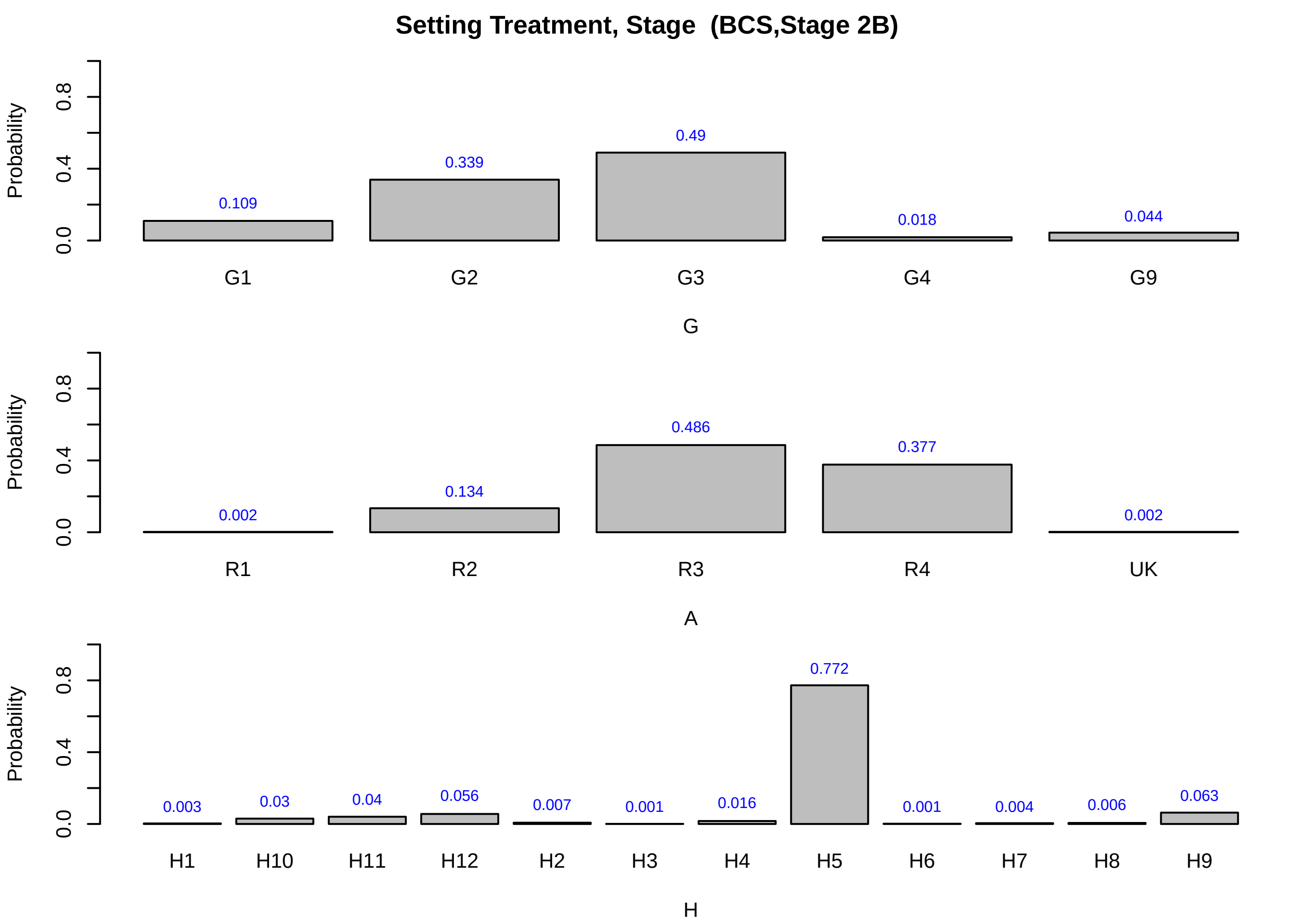
Setting Treatment, Stage (BCS,Stage 2B)
Probability
0.0
0.4
0.8
0.109
0.339
0.49
0.018
0.044
G1
G2
G3
G4
G9
G
Probability
0.0
0.4
0.8
0.002
0.134
0.486
0.377
0.002
R1
R2
R3
R4
UK
A
Probability
0.0
0.4
0.8
0.003
0.03
0.04
0.056
0.007
0.001
0.016
0.772
0.001
0.004
0.006
0.063
H1
H10
H11
H12
H2
H3
H4
H5
H6
H7
H8
H9
H

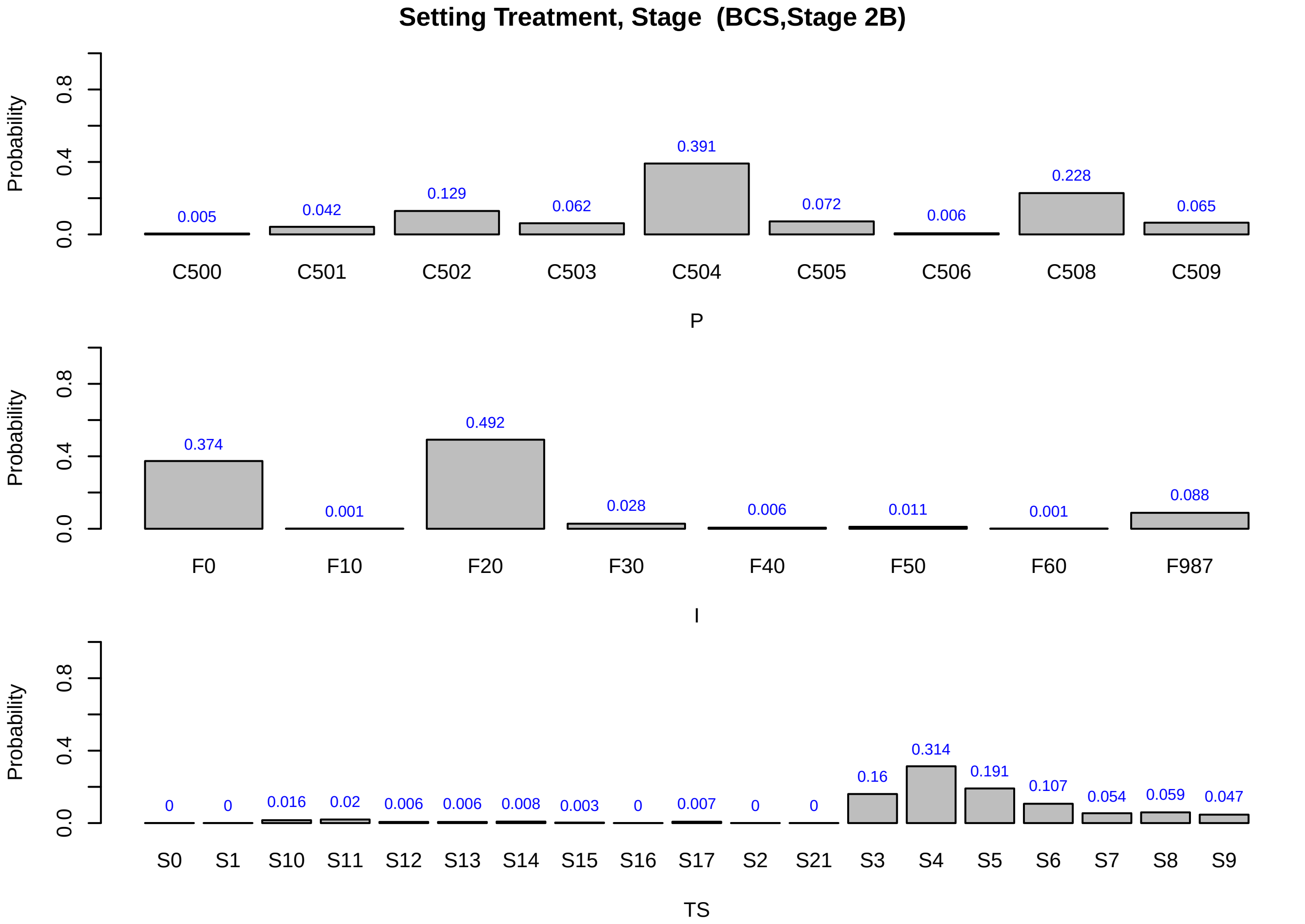
Setting Treatment, Stage (BCS,Stage 2B)
Probability
0.0
0.4
0.8
0.005
0.042
0.129
0.062
0.391
0.072
0.006
0.228
0.065
C500
C501
C502
C503
C504
C505
C506
C508
C509
P
Probability
0.0
0.4
0.8
0.374
0.001
0.492
0.028
0.006
0.011
0.001
0.088
F0
F10
F20
F30
F40
F50
F60
F987
I
Probability
0.0
0.4
0.8
0
0
0.016
0.02
0.006
0.006
0.008
0.003
0
0.007
0
0
0.16
0.314
0.191
0.107
0.054
0.059
0.047
S0
S1
S10
S11
S12
S13
S14
S15
S16
S17
S2
S21
S3
S4
S5
S6
S7
S8
S9
TS

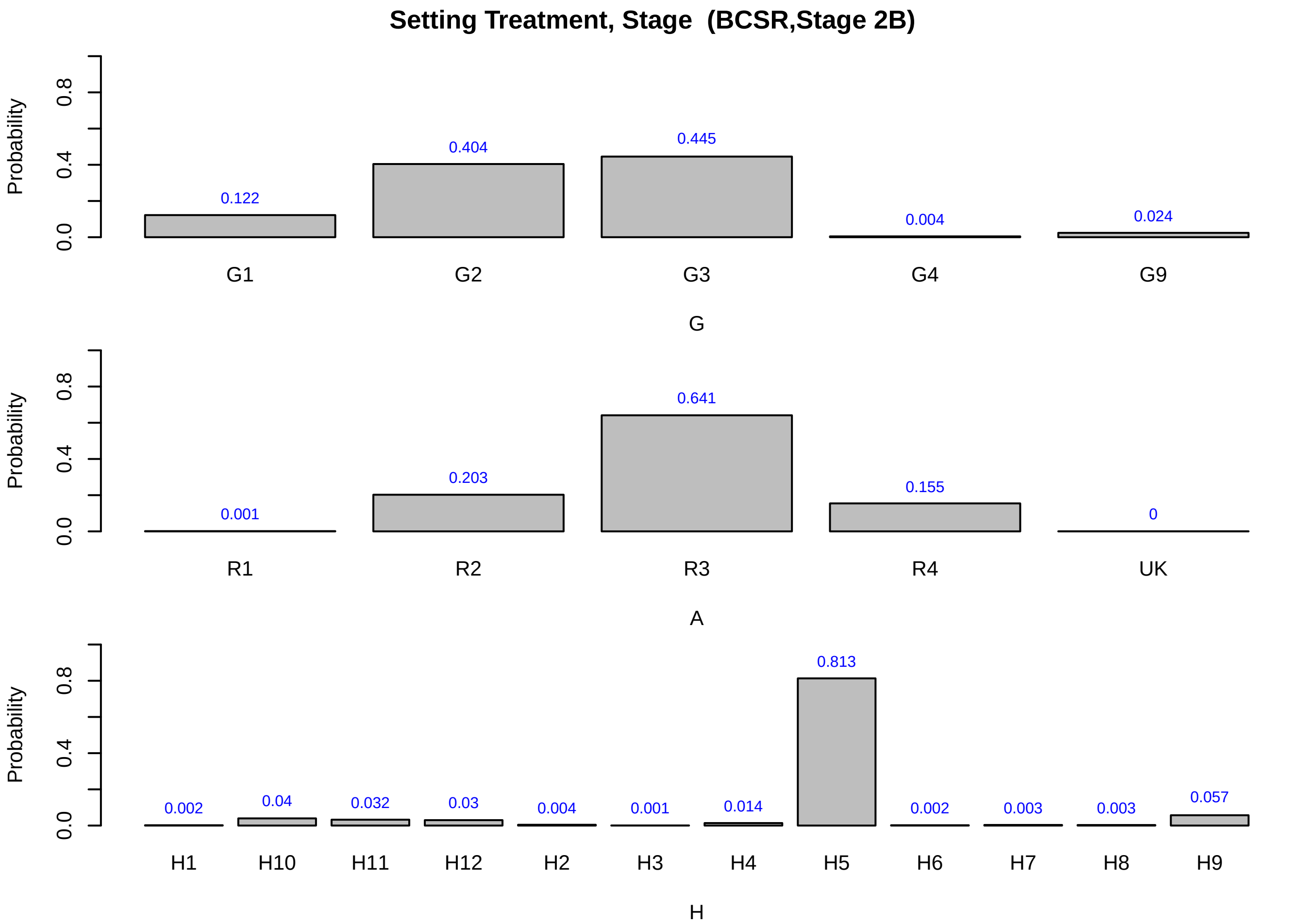
Setting Treatment, Stage (BCSR,Stage 2B)
Probability
0.0
0.4
0.8
0.122
0.404
0.445
0.004
0.024
G1
G2
G3
G4
G9
G
Probability
0.0
0.4
0.8
0.001
0.203
0.641
0.155
0
R1
R2
R3
R4
UK
A
Probability
0.0
0.4
0.8
0.002
0.04
0.032
0.03
0.004
0.001
0.014
0.813
0.002
0.003
0.003
0.057
H1
H10
H11
H12
H2
H3
H4
H5
H6
H7
H8
H9
H

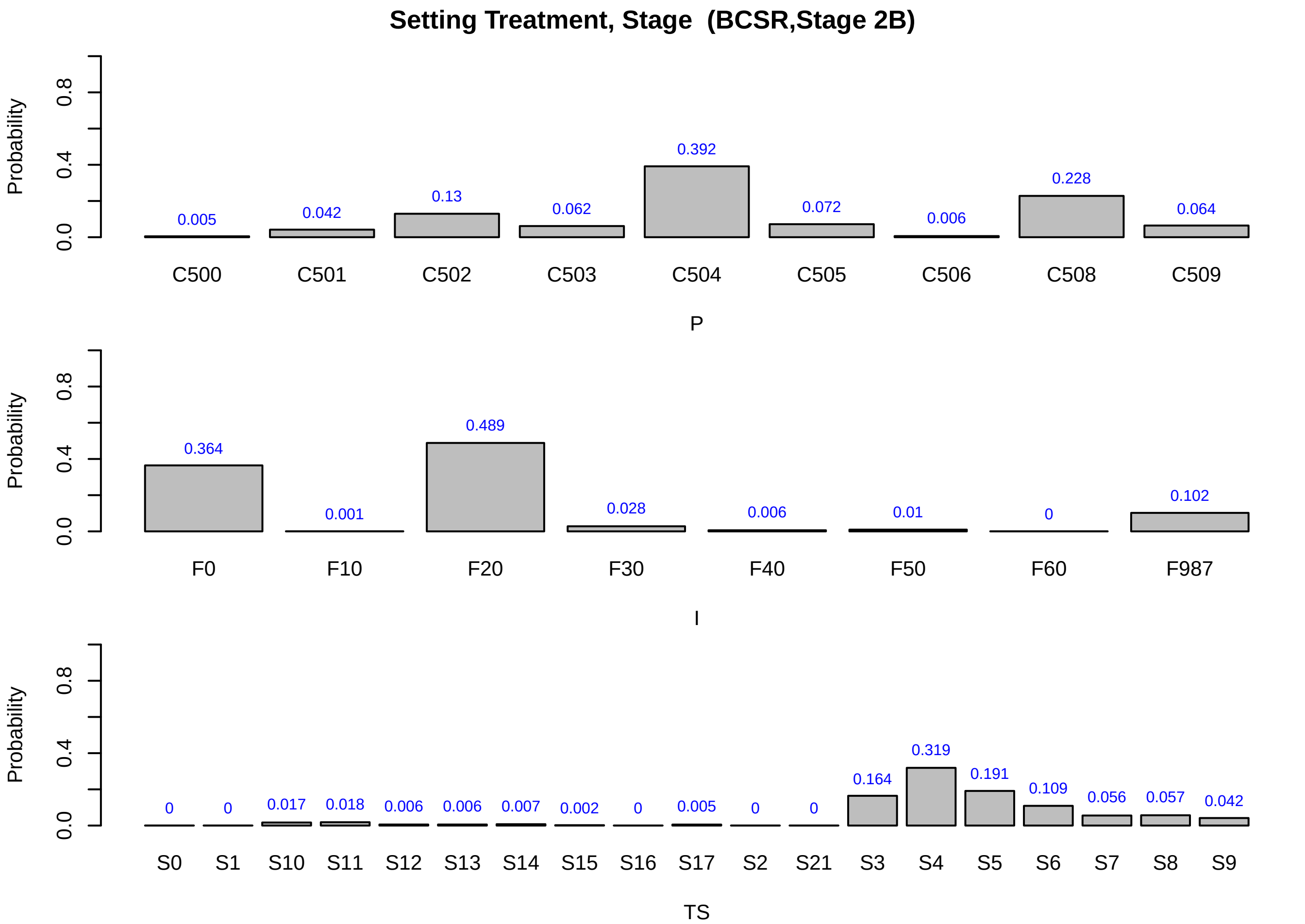
Setting Treatment, Stage (BCSR,Stage 2B)
Probability
0.0
0.4
0.8
0.005
0.042
0.13
0.062
0.392
0.072
0.006
0.228
0.064
C500
C501
C502
C503
C504
C505
C506
C508
C509
P
Probability
0.0
0.4
0.8
0.364
0.001
0.489
0.028
0.006
0.01
0
0.102
F0
F10
F20
F30
F40
F50
F60
F987
I
Probability
0.0
0.4
0.8
0
0
0.017
0.018
0.006
0.006
0.007
0.002
0
0.005
0
0
0.164
0.319
0.191
0.109
0.056
0.057
0.042
S0
S1
S10
S11
S12
S13
S14
S15
S16
S17
S2
S21
S3
S4
S5
S6
S7
S8
S9
TS

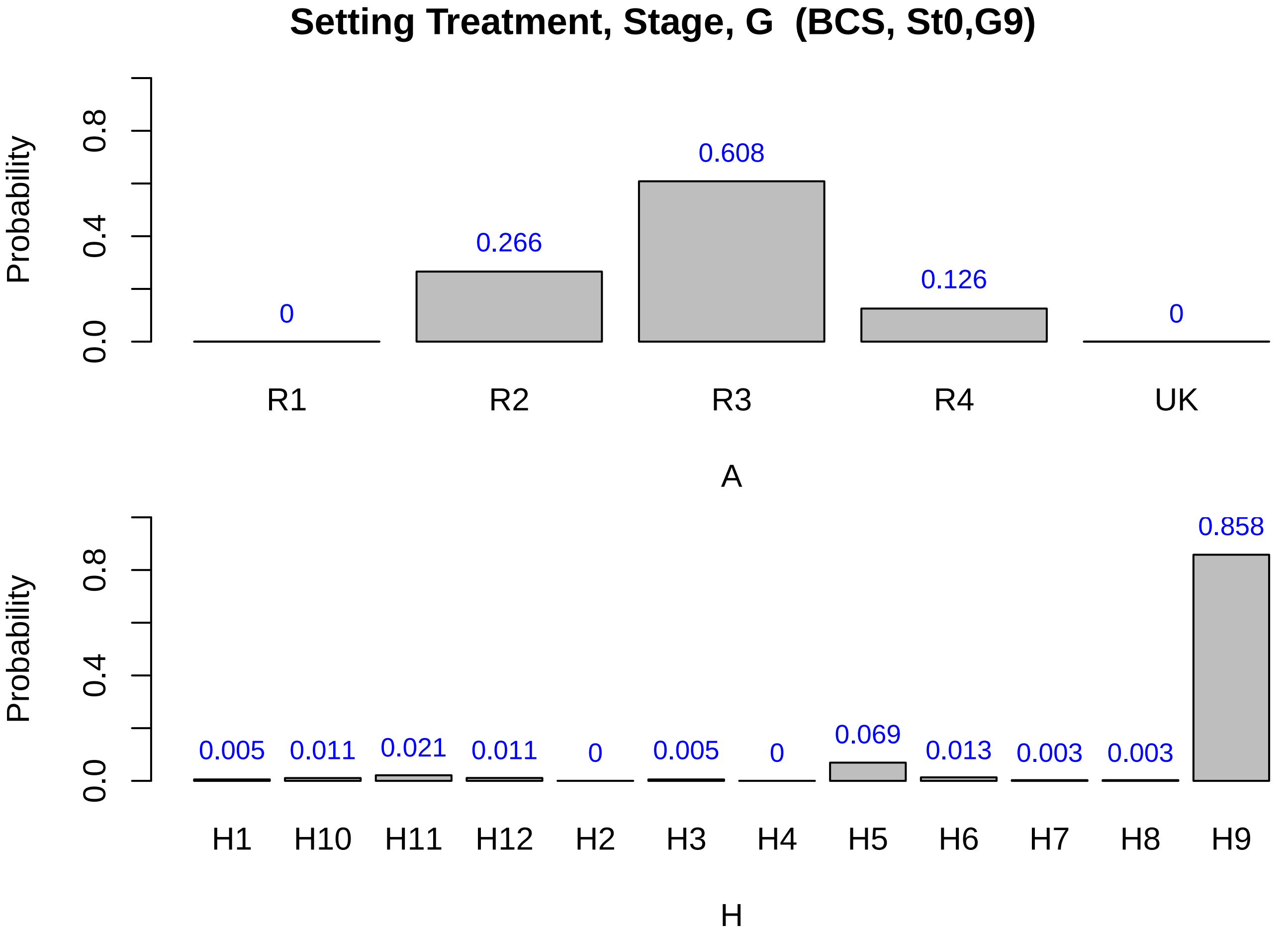

Setting Treatment, Stage, G (BCS, St0,G9)
Probability
0.0
0.4
0.8
0
0.266
0.608
0.126
0
R1
R2
R3
R4
UK
A
Probability
0.0
0.4
0.8
0.005
0.011
0.021
0.011
0
0.005
0
0.069
0.013
0.003
0.003
0.858
H1
H10
H11
H12
H2
H3
H4
H5
H6
H7
H8
H9
H

## Setting Treatment, Stage, G (BCS, St0,G9)

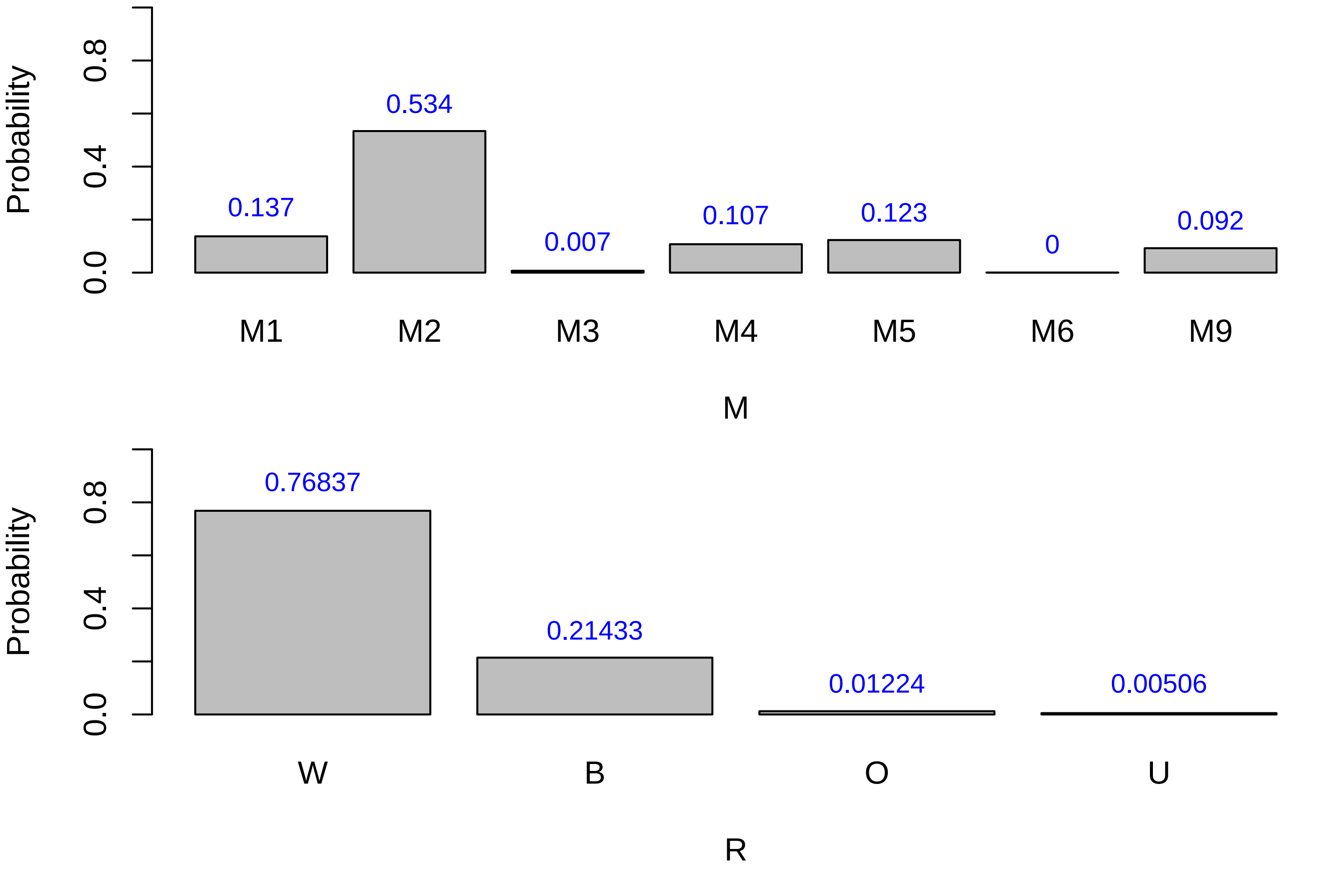

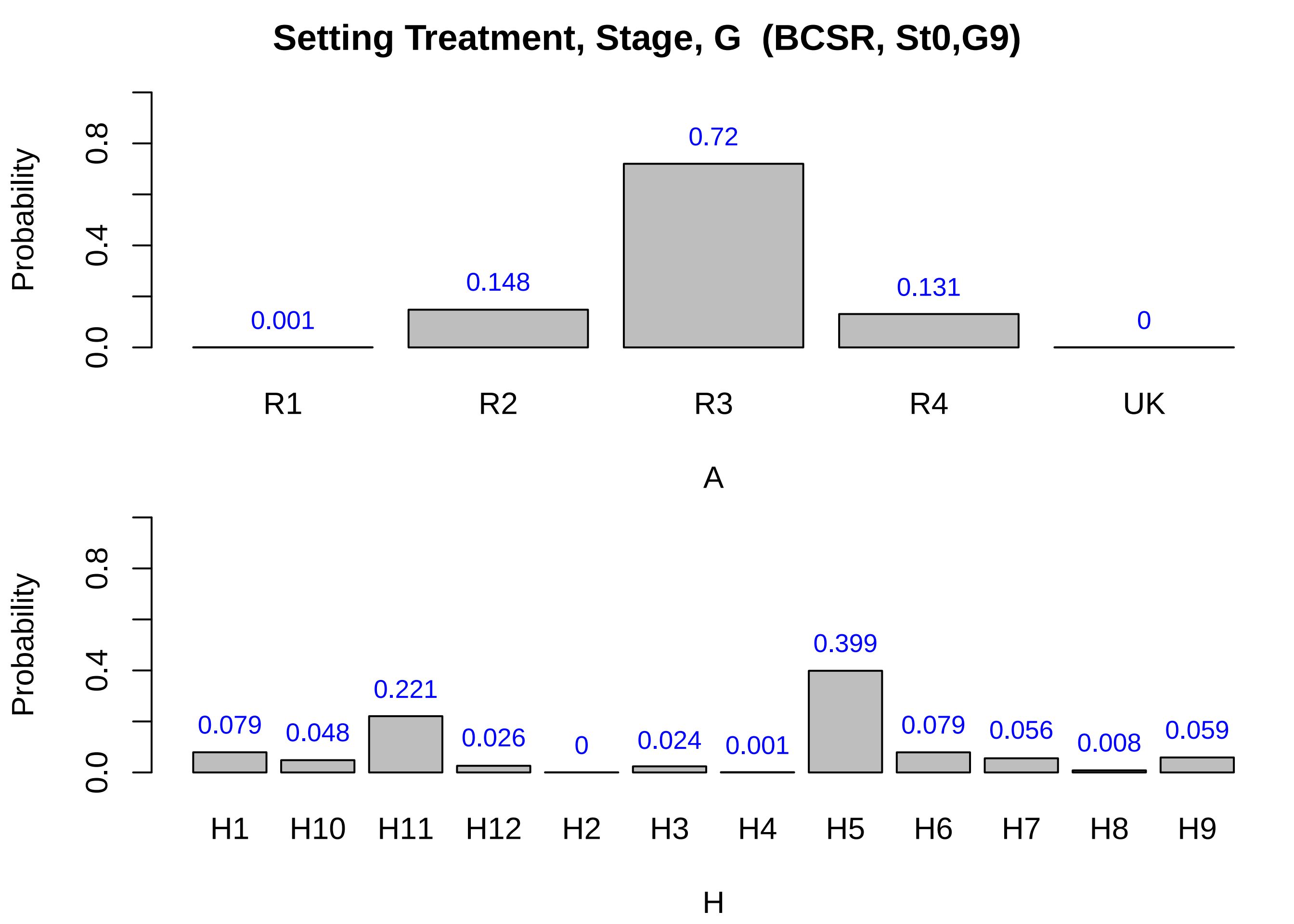

Setting Treatment, Stage, G (BCSR, St0,G9)
Probability
0.0
0.4
0.8
0.001
0.148
0.72
0.131
0
R1
R2
R3
R4
UK
A
Probability
0.0
0.4
0.8
0.079
0.048
0.221
0.026
0
0.024
0.001
0.399
0.079
0.056
0.008
0.059
H1
H10
H11
H12
H2
H3
H4
H5
H6
H7
H8
H9
H

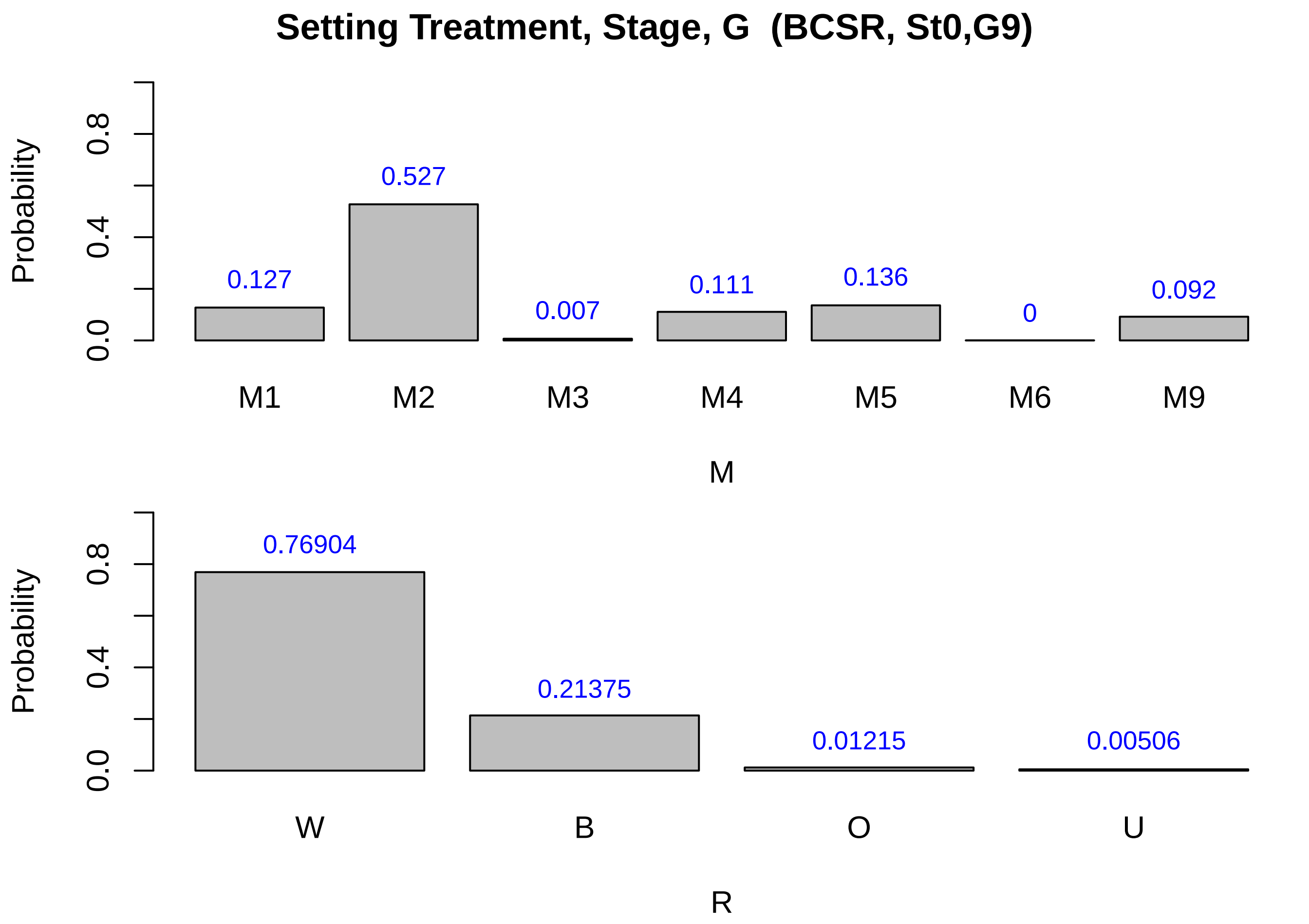
Setting Treatment, Stage, G (BCSR, St0,G9)
Probability
0.0
0.4
0.8
0.127
0.527
0.007
0.111
0.136
0
0.092
M1
M2
M3
M4
M5
M6
M9
M
Probability
0.0
0.4
0.8
0.76904
0.21375
0.01215
0.00506
W
B
O
U
R

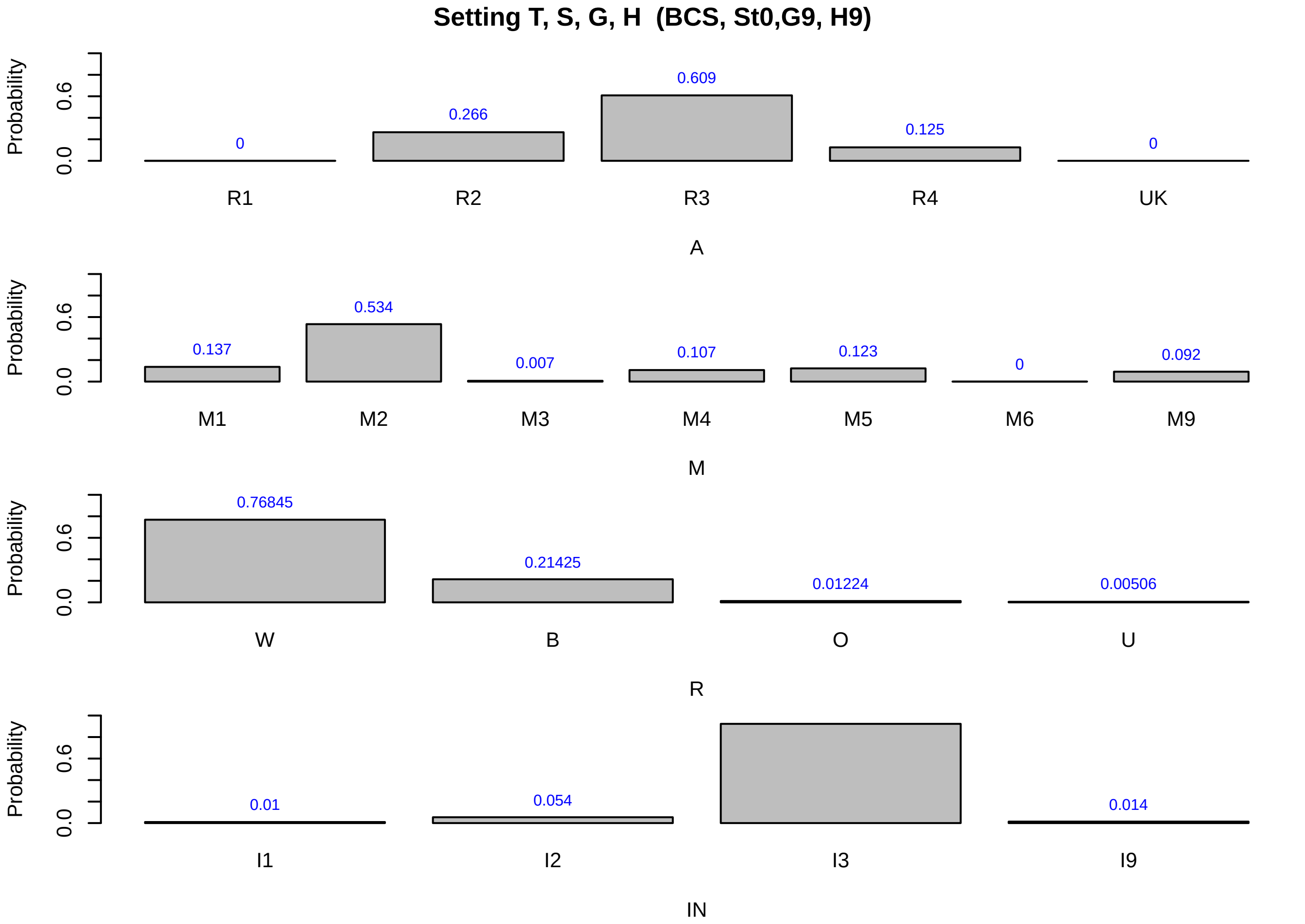

Setting T, S, G, H (BCS, St0,G9, H9)
Probability
0.0
0.6
0
0.266
0.609
0.125
0
R1
R2
R3
R4
UK
A
0.137
0.534
0.007
0.107
0.123
0
0.092
M1
M2
M3
M4
M5
M6
M9
M
0.76845
0.21425
0.01224
0.00506
W
B
O
U
R
0.01
0.054
0.014
I1
I2
I3
I9
IN

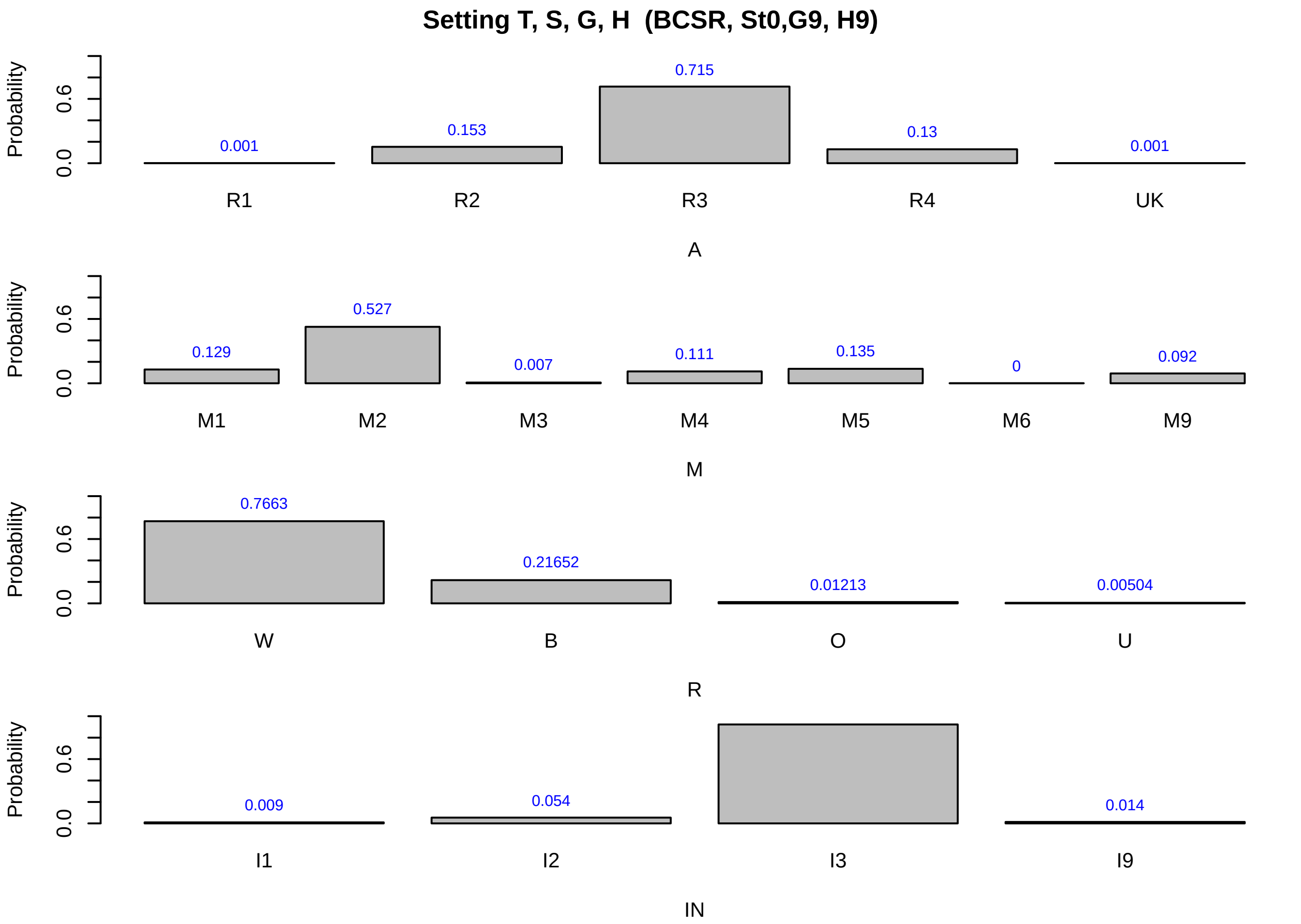
Setting T, S, G, H (BCSR, St0,G9, H9)
Probability
0.0
0.6
0.001
0.153
0.715
0.13
0.001
R1
R2
R3
R4
UK
A
0.129
0.527
0.007
0.111
0.135
0
0.092
M1
M2
M3
M4
M5
M6
M9
M
0.7663
0.21652
0.01213
0.00504
W
B
O
U
R
0.009
0.054
0.014
I1
I2
I3
I9
IN

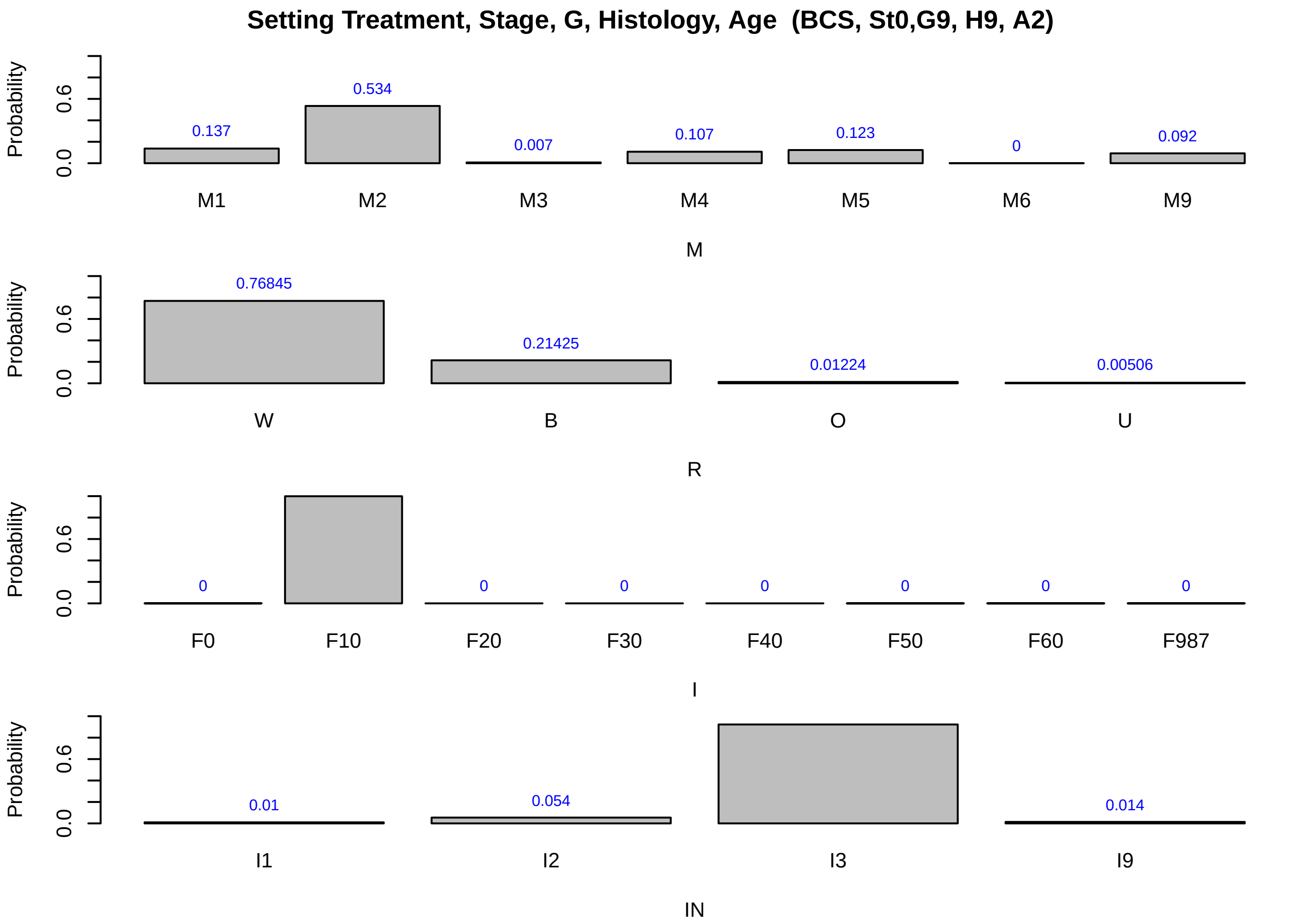

Setting Treatment, Stage, G, Histology, Age (BCS, St0,G9, H9, A2)
Probability
0.0
0.6
0.137
0.534
0.007
0.107
0.123
0
0.092
M1
M2
M3
M4
M5
M6
M9
M
0.76845
0.21425
0.01224
0.00506
W
B
O
U
R
0
0
0
0
0
0
0
F0
F10
F20
F30
F40
F50
F60
F987
I
0.01
0.054
0.014
I1
I2
I3
I9
IN

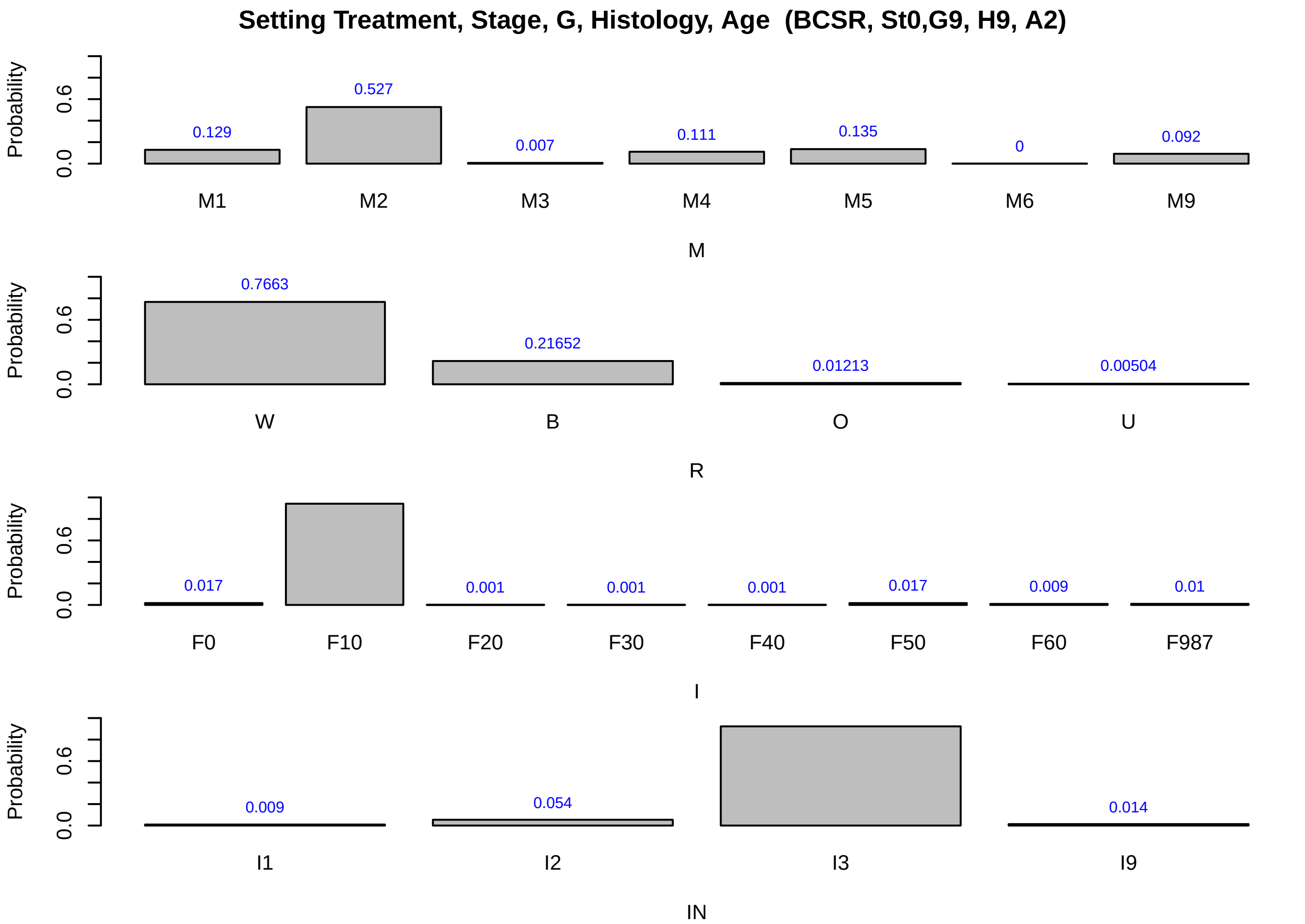

Setting Treatment, Stage, G, Histology, Age (BCSR, St0,G9, H9, A2)
Probability
0.0
0.6
0.129
0.527
0.007
0.111
0.135
0
0.092
M1
M2
M3
M4
M5
M6
M9
M
0.7663
0.21652
0.01213
0.00504
W
B
O
U
R
0.017
0.001
0.001
0.001
0.017
0.009
0.01
F0
F10
F20
F30
F40
F50
F60
F987
I
0.009
0.054
0.014
I1
I2
I3
I9
IN

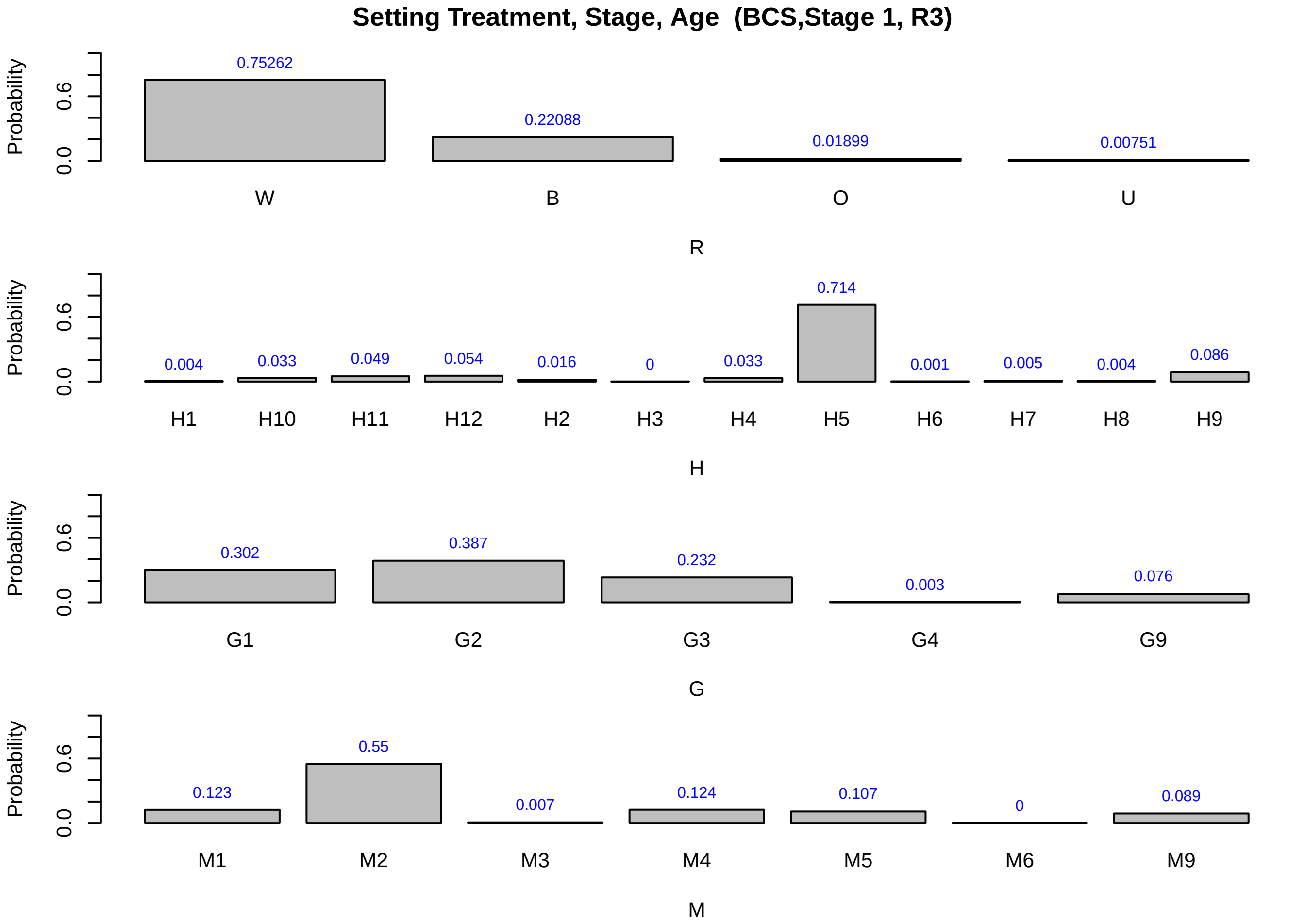
Setting Treatment, Stage, Age (BCS,Stage 1, R3)
Probability
0.0
0.6
0.75262
0.22088
0.01899
0.00751
W
B
O
U
R
0.004
0.033
0.049
0.054
0.016
0
0.033
0.714
0.001
0.005
0.004
0.086
H1
H10
H11
H12
H2
H3
H4
H5
H6
H7
H8
H9
H
0.302
0.387
0.232
0.003
0.076
G1
G2
G3
G4
G9
G
0.123
0.55
0.007
0.124
0.107
0
0.089
M1
M2
M3
M4
M5
M6
M9
M

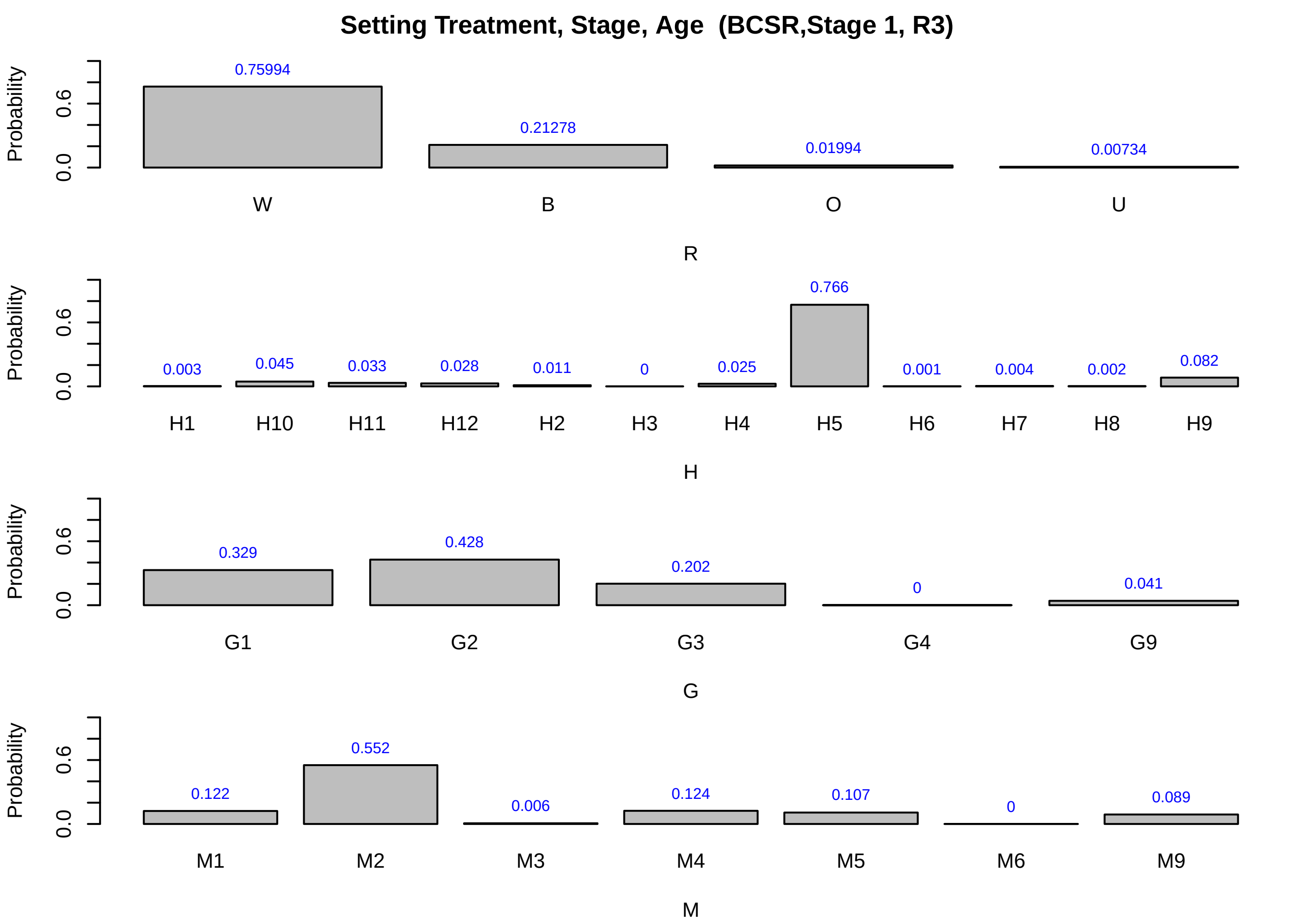

Setting Treatment, Stage, Age (BCSR,Stage 1, R3)
Probability
0.0
0.6
0.75994
0.21278
0.01994
0.00734
W
B
O
U
R
0.003
0.045
0.033
0.028
0.011
0
0.025
0.766
0.001
0.004
0.002
0.082
H1
H10
H11
H12
H2
H3
H4
H5
H6
H7
H8
H9
H
0.329
0.428
0.202
0
0.041
G1
G2
G3
G4
G9
G
0.122
0.552
0.006
0.124
0.107
0
0.089
M1
M2
M3
M4
M5
M6
M9
M

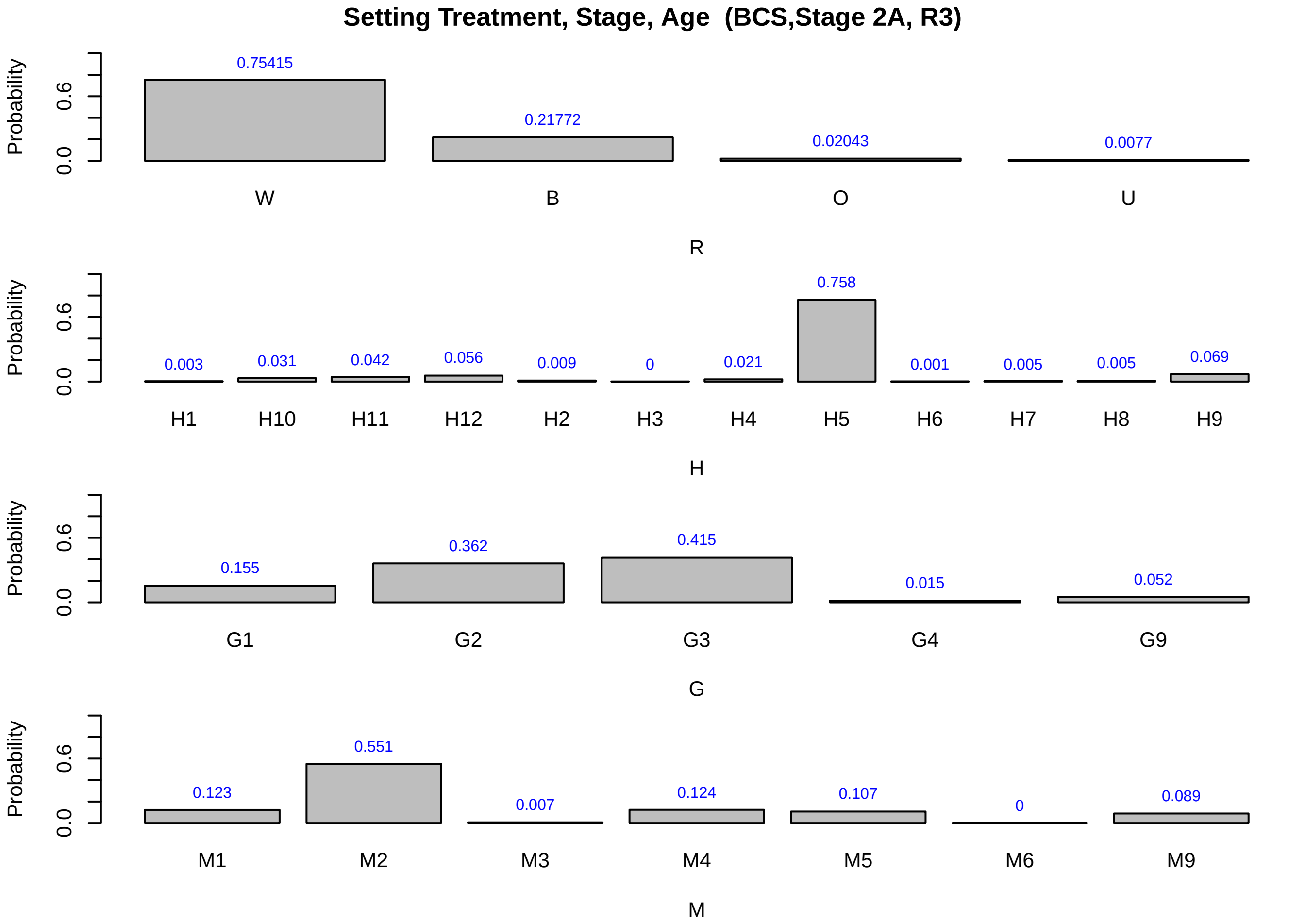
Setting Treatment, Stage, Age (BCS,Stage 2A, R3)
Probability
0.0
0.6
0.75415
0.21772
0.02043
0.0077
W
B
O
U
R
Probability
0.0
0.6
0.003
0.031
0.042
0.056
0.009
0
0.021
0.758
0.001
0.005
0.005
0.069
H1
H10
H11
H12
H2
H3
H4
H5
H6
H7
H8
H9
H
Probability
0.0
0.6
0.155
0.362
0.415
0.015
0.052
G1
G2
G3
G4
G9
G
Probability
0.0
0.6
0.123
0.551
0.007
0.124
0.107
0
0.089
M1
M2
M3
M4
M5
M6
M9
M

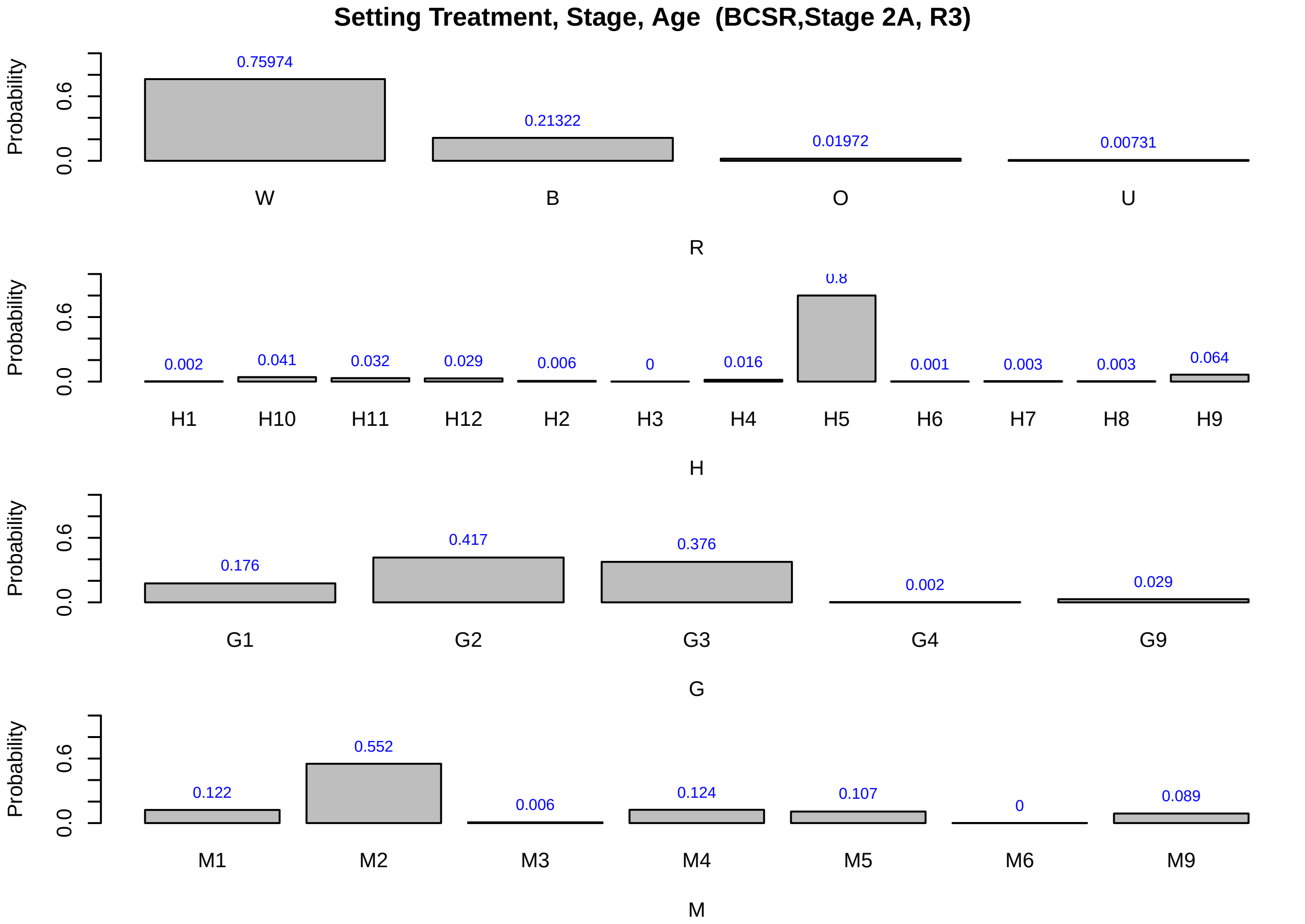

Setting Treatment, Stage, Age (BCSR,Stage 2A, R3)
Probability
0.0
0.6
0.75974
0.21322
0.01972
0.00731
W
B
O
U
R
0.002
0.041
0.032
0.029
0.006
0
0.016
0.8
0.001
0.003
0.003
0.064
H1
H10
H11
H12
H2
H3
H4
H5
H6
H7
H8
H9
H
0.176
0.417
0.376
0.002
0.029
G1
G2
G3
G4
G9
G
0.122
0.552
0.006
0.124
0.107
0
0.089
M1
M2
M3
M4
M5
M6
M9
M

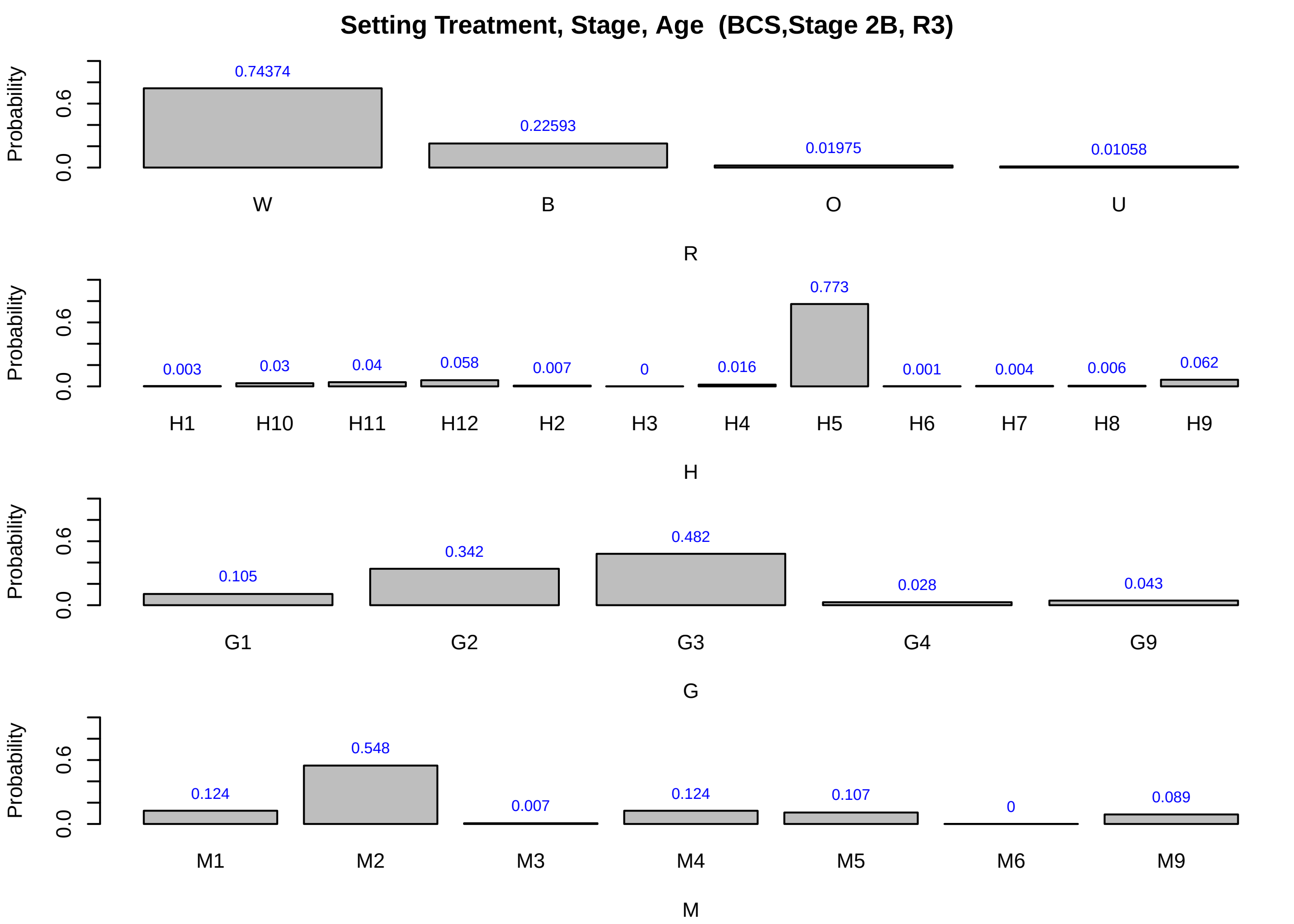
Setting Treatment, Stage, Age (BCS,Stage 2B, R3)
Probability
0.0
0.6
0.74374
0.22593
0.01975
0.01058
W
B
O
U
R
0.003
0.03
0.04
0.058
0.007
0
0.016
0.773
0.001
0.004
0.006
0.062
H1
H10
H11
H12
H2
H3
H4
H5
H6
H7
H8
H9
H
0.105
0.342
0.482
0.028
0.043
G1
G2
G3
G4
G9
G
0.124
0.548
0.007
0.124
0.107
0
0.089
M1
M2
M3
M4
M5
M6
M9
M

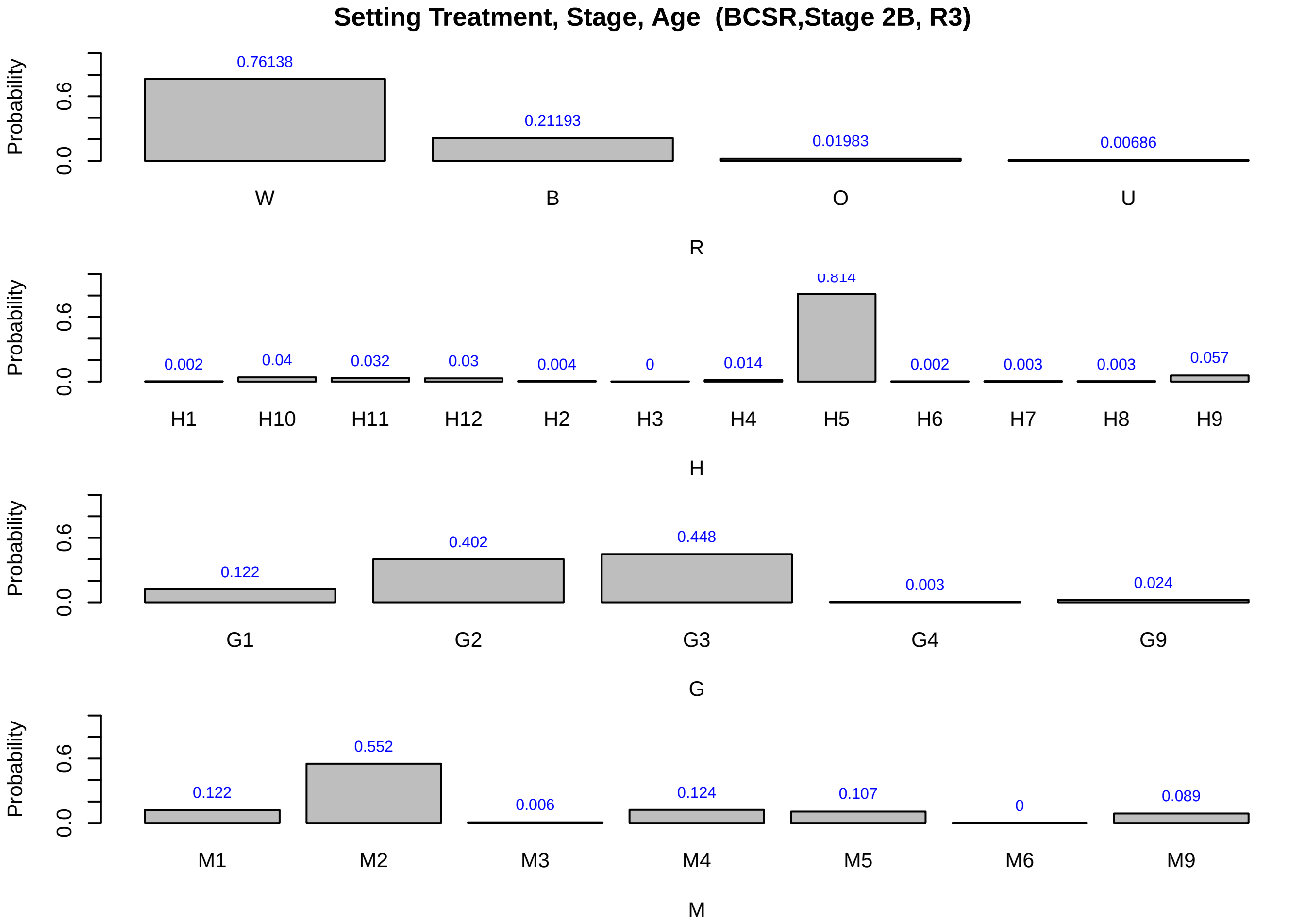
Setting Treatment, Stage, Age (BCSR,Stage 2B, R3)
Probability
0.0
0.6
0.76138
0.21193
0.01983
0.00686
W
B
O
U
R
0.002
0.04
0.032
0.03
0.004
0
0.014
0.814
0.002
0.003
0.003
0.057
H1
H10
H11
H12
H2
H3
H4
H5
H6
H7
H8
H9
H
0.122
0.402
0.448
0.003
0.024
G1
G2
G3
G4
G9
G
0.122
0.552
0.006
0.124
0.107
0
0.089
M1
M2
M3
M4
M5
M6
M9
M

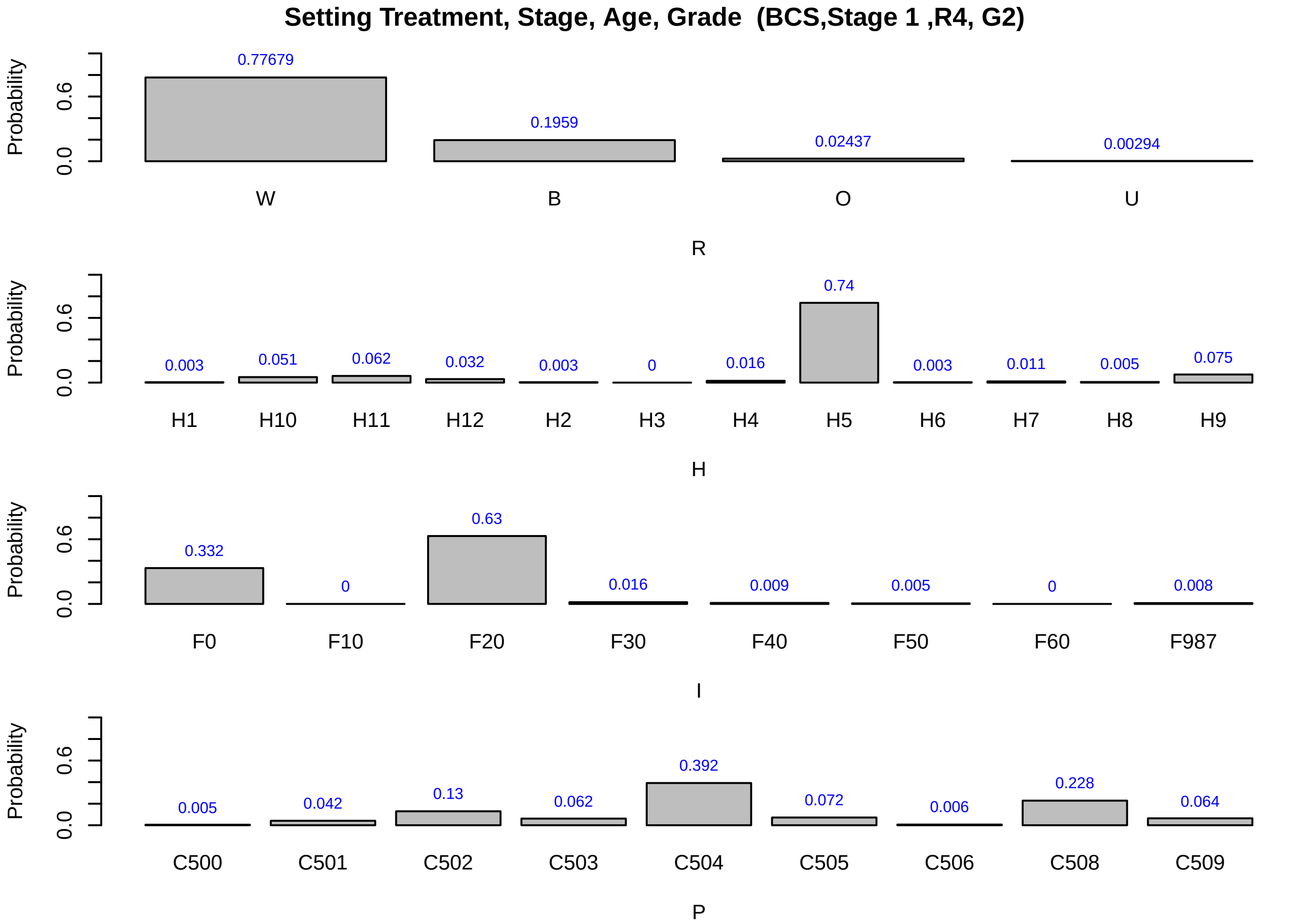
Setting Treatment, Stage, Age, Grade (BCS,Stage 1 ,R4, G2)
Probability
0.0
0.6
0.77679
0.1959
0.02437
0.00294
W
B
O
U
R
0.003
0.051
0.062
0.032
0.003
0
0.016
0.74
0.003
0.011
0.005
0.075
H1
H10
H11
H12
H2
H3
H4
H5
H6
H7
H8
H9
H
0.332
0
0.63
0.016
0.009
0.005
0
0.008
F0
F10
F20
F30
F40
F50
F60
F987
I
0.005
0.042
0.13
0.062
0.392
0.072
0.006
0.228
0.064
C500
C501
C502
C503
C504
C505
C506
C508
C509
P

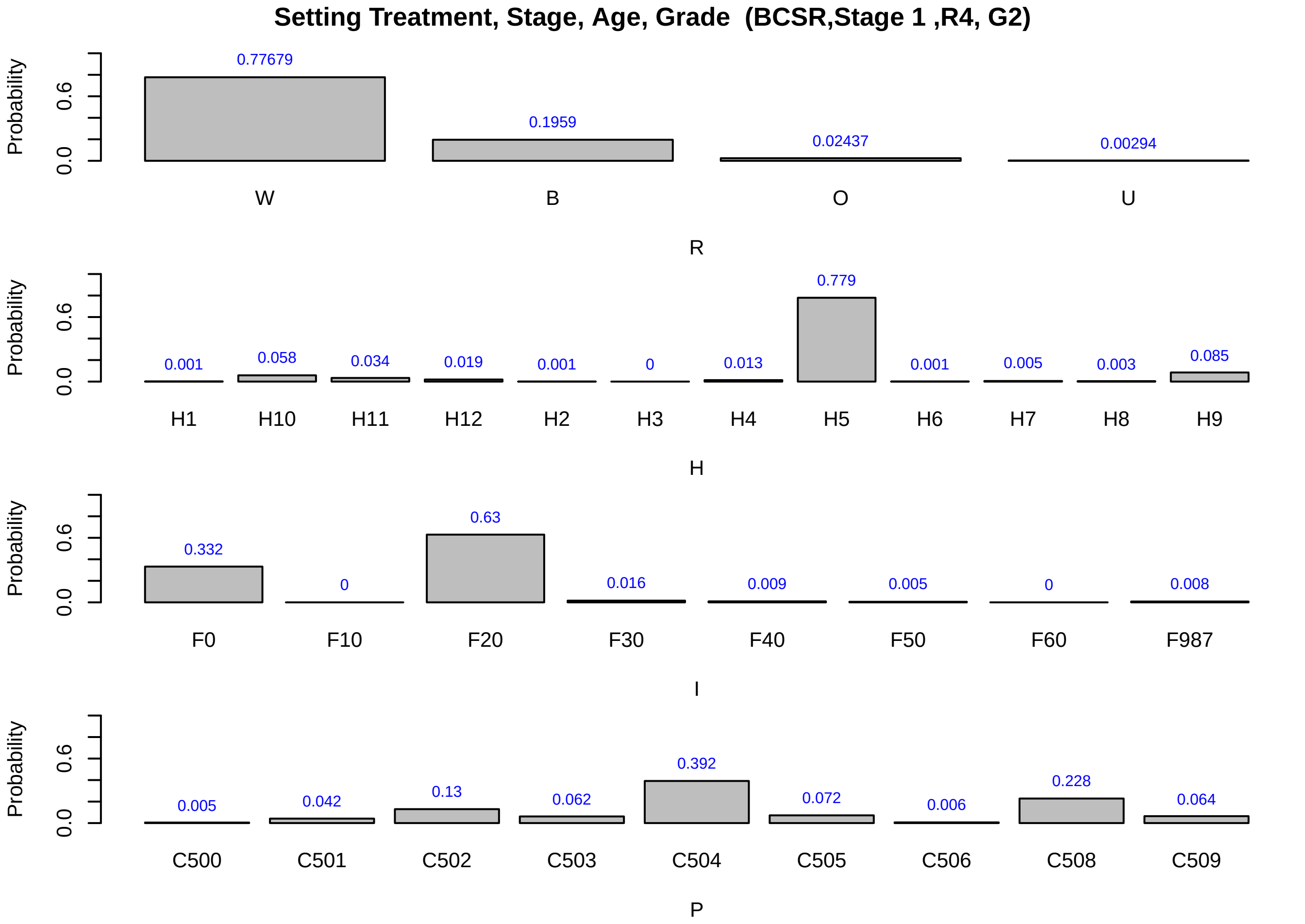

Setting Treatment, Stage, Age, Grade (BCSR,Stage 1 ,R4, G2)
Probability
0.0
0.6
0.77679
0.1959
0.02437
0.00294
W
B
O
U
R
0.001
0.058
0.034
0.019
0.001
0
0.013
0.779
0.001
0.005
0.003
0.085
H1
H10
H11
H12
H2
H3
H4
H5
H6
H7
H8
H9
H
0.332
0
0.63
0.016
0.009
0.005
0
0.008
F0
F10
F20
F30
F40
F50
F60
F987
I
0.005
0.042
0.13
0.062
0.392
0.072
0.006
0.228
0.064
C500
C501
C502
C503
C504
C505
C506
C508
C509
P

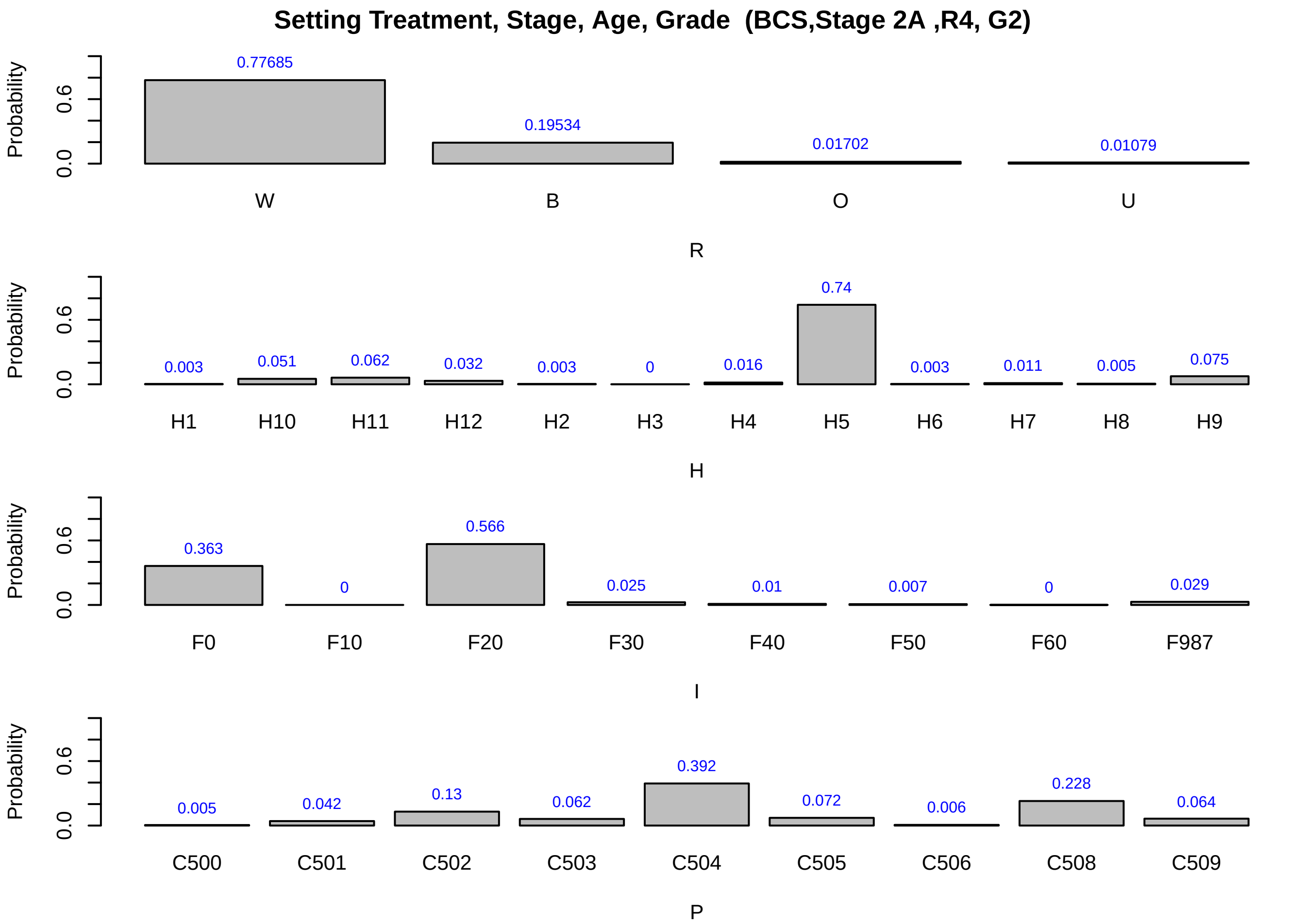
Setting Treatment, Stage, Age, Grade (BCS,Stage 2A ,R4, G2)
Probability
0.0
0.6
0.77685
0.19534
0.01702
0.01079
W
B
O
U
R
0.003
0.051
0.062
0.032
0.003
0
0.016
0.74
0.003
0.011
0.005
0.075
H1
H10
H11
H12
H2
H3
H4
H5
H6
H7
H8
H9
H
0.363
0
0.566
0.025
0.01
0.007
0
0.029
F0
F10
F20
F30
F40
F50
F60
F987
I
0.005
0.042
0.13
0.062
0.392
0.072
0.006
0.228
0.064
C500
C501
C502
C503
C504
C505
C506
C508
C509
P

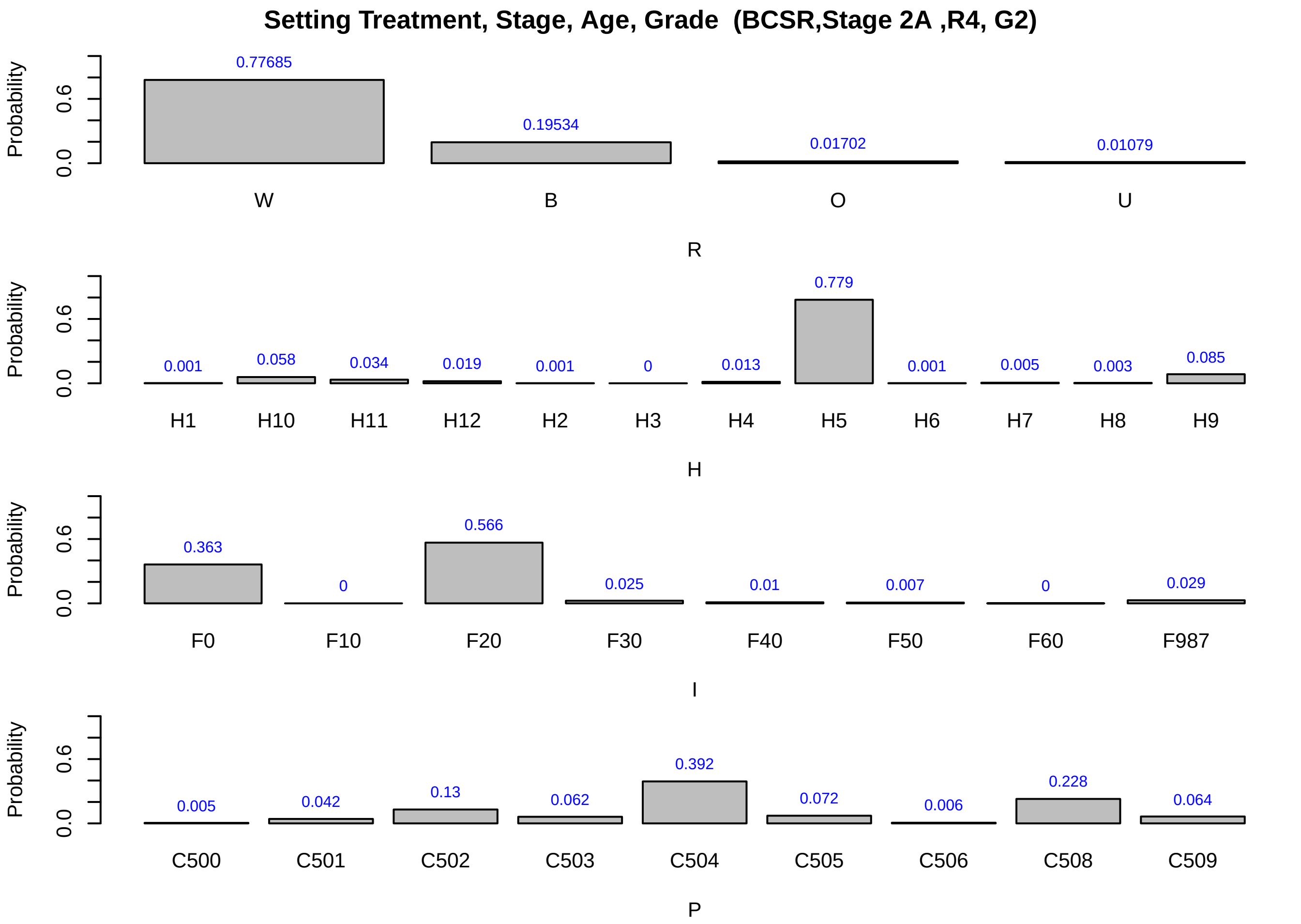

Setting Treatment, Stage, Age, Grade (BCSR,Stage 2A ,R4, G2)
Probability
0.0
0.6
0.77685
0.19534
0.01702
0.01079
W
B
O
U
R
Probability
0.0
0.6
0.001
0.058
0.034
0.019
0.001
0
0.013
0.779
0.001
0.005
0.003
0.085
H1
H10
H11
H12
H2
H3
H4
H5
H6
H7
H8
H9
H
Probability
0.0
0.6
0.363
0
0.566
0.025
0.01
0.007
0
0.029
F0
F10
F20
F30
F40
F50
F60
F987
I
Probability
0.0
0.6
0.005
0.042
0.13
0.062
0.392
0.072
0.006
0.228
0.064
C500
C501
C502
C503
C504
C505
C506
C508
C509
P

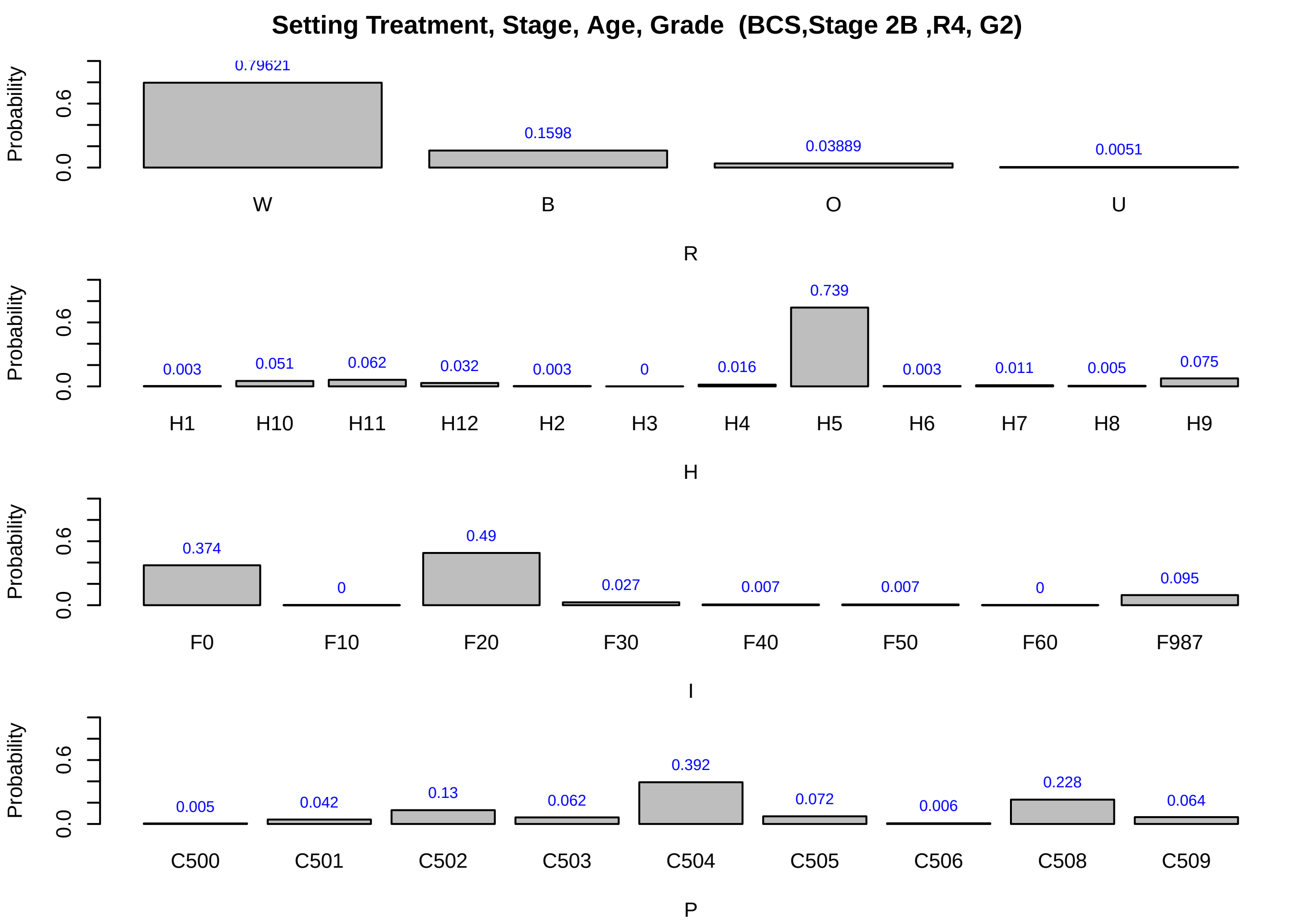

Setting Treatment, Stage, Age, Grade (BCS,Stage 2B ,R4, G2)
Probability
0.0
0.6
0.79621
0.1598
0.03889
0.0051
W
B
O
U
R
0.003
0.051
0.062
0.032
0.003
0
0.016
0.739
0.003
0.011
0.005
0.075
H1
H10
H11
H12
H2
H3
H4
H5
H6
H7
H8
H9
H
0.374
0
0.49
0.027
0.007
0.007
0
0.095
F0
F10
F20
F30
F40
F50
F60
F987
I
0.005
0.042
0.13
0.062
0.392
0.072
0.006
0.228
0.064
C500
C501
C502
C503
C504
C505
C506
C508
C509
P

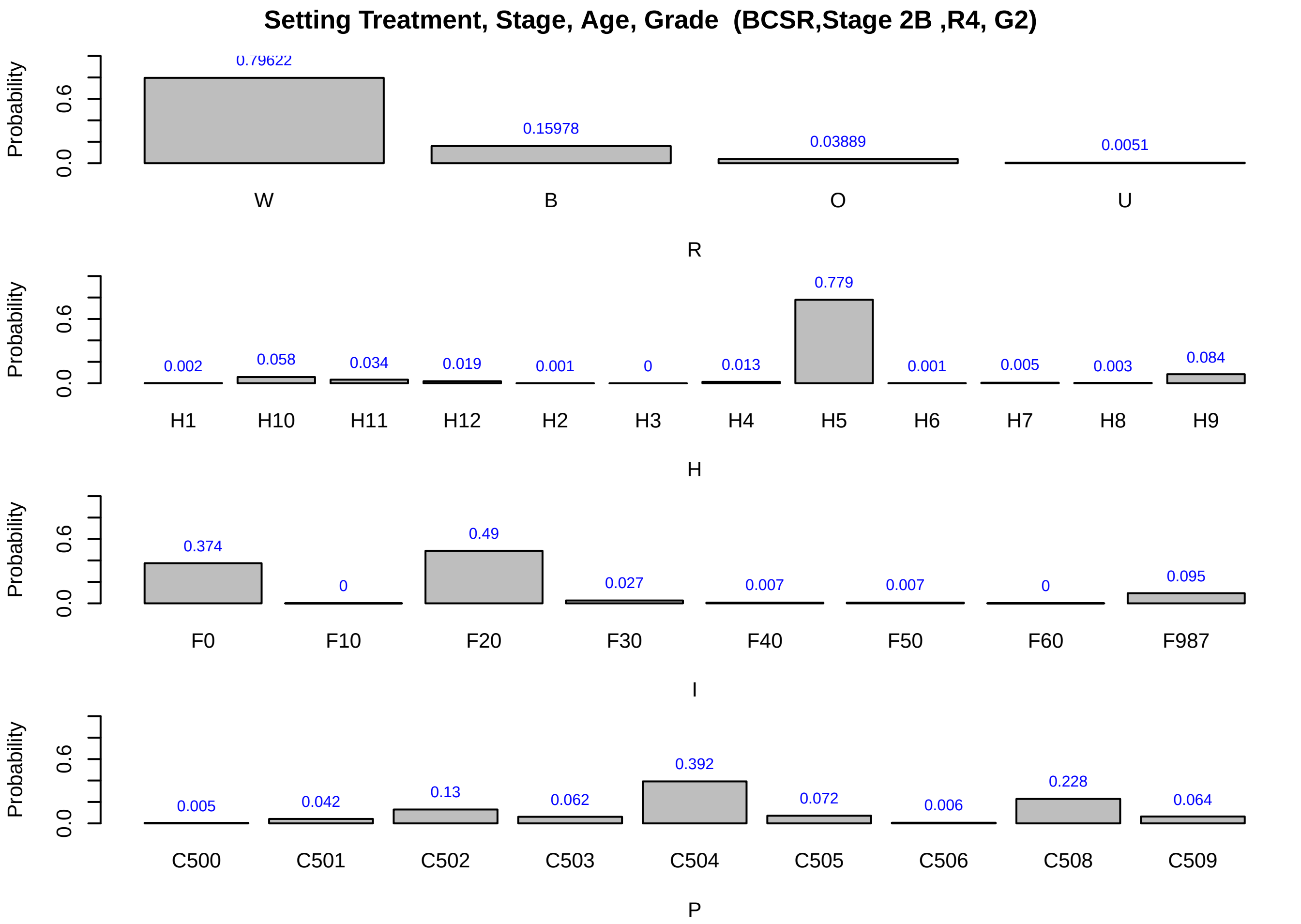
Setting Treatment, Stage, Age, Grade (BCSR,Stage 2B ,R4, G2)
Probability
0.0
0.6
0.79622
0.15978
0.03889
0.0051
W
B
O
U
R
0.002
0.058
0.034
0.019
0.001
0
0.013
0.779
0.001
0.005
0.003
0.084
H1
H10
H11
H12
H2
H3
H4
H5
H6
H7
H8
H9
H
0.374
0
0.49
0.027
0.007
0.007
0
0.095
F0
F10
F20
F30
F40
F50
F60
F987
I
0.005
0.042
0.13
0.062
0.392
0.072
0.006
0.228
0.064
C500
C501
C502
C503
C504
C505
C506
C508
C509
P

## Section 2

### Logistic Regression

```
Call:
NULL

Deviance Residuals:
    Min       1Q   Median       3Q      Max
-1.4046   0.1584   0.2097   0.2999   1.3922

Coefficients: (2 not defined because of singularities)

              Estimate Std. Error z value Pr(>|z|)
(Intercept)   2.767556   2.609521   1.061   0.2889
MM2           0.069165   0.095495   0.724   0.4689
MM3           0.033588   0.064824   0.518   0.6044
MM4           0.022406   0.077323   0.290   0.7720
MM5          -0.050714   0.079458  -0.638   0.5233
MM6           0.148644  17.358899   0.009   0.9932
MM9          -0.024786   0.072639  -0.341   0.7329
Yy2008        0.049027   0.072171   0.679   0.4969
Yy2009        0.134075   0.074285   1.805   0.0711 .
Yy2010        0.130519   0.075475   1.729   0.0838 .
Yy2011        0.112847   0.075715   1.490   0.1361
Yy2012        0.144490   0.078155   1.849   0.0645 .
PC501         0.068045   0.153239   0.444   0.6570
PC502         0.074363   0.218694   0.340   0.7338
PC503         0.114347   0.167341   0.683   0.4944
PC504         0.235622   0.322957   0.730   0.4656
PC505         0.142801   0.180380   0.792   0.4286
PC506        -0.001869   0.072339  -0.026   0.9794
PC508         0.160588   0.278224   0.577   0.5638
PC509         0.020948   0.187138   0.112   0.9109
GG2           0.071793   0.079256   0.906   0.3650
GG3           0.056298   0.083707   0.673   0.5012
GG4           0.019441   0.057203   0.340   0.7340
GG9          -0.334443   0.059836  -5.589 2.28e-08 ***
IF10         -6.783328 303.457327  -0.022   0.9822
IF20          0.036738   0.078946   0.465   0.6417
IF30          0.020570   0.067220   0.306   0.7596
IF40          0.003882   0.065634   0.059   0.9528
IF50         -0.026574   0.055901  -0.475   0.6345
IF60         -0.331083  30.509129  -0.011   0.9913
IF987         0.071001   0.073618   0.964   0.3348
SSt1         -7.001996 336.707539  -0.021   0.9834
SSt2A        -5.623832 263.658326  -0.021   0.9830
SSt2B        -3.597776 167.573090  -0.021   0.9829
WNR2         -1.622300  28.160115  -0.058   0.9541
BB3                 NA         NA      NA       NA
RB           -0.039997   0.060442  -0.662   0.5081
RO           -0.009470   0.065555  -0.144   0.8851
RU            0.032826   0.061658   0.532   0.5945
INI2         -0.078053   0.157657  -0.495   0.6205
INI3          0.014292   0.176068   0.081   0.9353
INI9         -0.035418   0.090939  -0.389   0.6969
```

```
AR2           0.301660   0.914998   0.330   0.7416
AR3           0.365690   1.115141   0.328   0.7430
AR4          -0.168549   0.883965  -0.191   0.8488
AUK                 NA         NA      NA       NA
HH10         -0.009579   0.086964  -0.110   0.9123
HH11          0.058009   0.101371   0.572   0.5672
HH12         -0.135148   0.072142  -1.873   0.0610 .
HH2          -0.098420   0.058485  -1.683   0.0924 .
HH3           0.073713   0.079001   0.933   0.3508
HH4          -0.056806   0.069848  -0.813   0.4161
HH5          -0.180672   0.154690  -1.168   0.2428
HH6           0.090802   0.080501   1.128   0.2593
HH7           0.005665   0.060765   0.093   0.9257
HH8          -0.018538   0.056336  -0.329   0.7421
HH9          -0.456152   0.101515  -4.493 7.01e-06 ***
TSS1          0.095230   0.087257   1.091   0.2751
TSS10        -0.017294   0.052464  -0.330   0.7417
TSS11        -0.015296   0.047928  -0.319   0.7496
TSS12        -0.025793   0.042182  -0.611   0.5409
TSS13        -0.015164   0.043501  -0.349   0.7274
TSS14        -0.071321   0.040083  -1.779   0.0752 .
TSS15         0.206526  17.318608   0.012   0.9905
TSS16        -0.038489   0.042228  -0.911   0.3621
TSS17         0.009088   0.055857   0.163   0.8708
TSS2          0.052911   0.087882   0.602   0.5471
TSS21        -0.146772   0.060878  -2.411   0.0159 *
TSS3          0.048246   0.081895   0.589   0.5558
TSS4         -0.026054   0.078237  -0.333   0.7391
TSS5          0.003632   0.070257   0.052   0.9588
TSS6         -0.036249   0.062165  -0.583   0.5598
TSS7         -0.006259   0.063786  -0.098   0.9218
TSS8         -0.056153   0.052160  -1.077   0.2817
TSS9         -0.059533   0.048116  -1.237   0.2160
---
Signif. codes:  0 ‘***’ 0.001 ‘**’ 0.01 ‘*’ 0.05 ‘.’ 0.1 ‘ ’ 1

(Dispersion parameter for binomial family taken to be 1)

    Null deviance: 2707.3  on 8216  degrees of freedom
Residual deviance: 2224.2  on 8144  degrees of freedom
AIC: 926.76

Number of Fisher Scoring iterations: 14

---------------------------------------------------------------------------
```

## glm variable importance

```
  only 20 most important variables shown (out of 72)

       Overall
GG9     100.00
HH9      80.36
TSS21    43.05
HH12     33.41
Yy2012   32.97
Yy2009   32.19
TSS14    31.73
Yy2010   30.83
HH2      30.00
Yy2011   26.55
TSS9     22.02
HH5      20.77
HH6      20.06
TSS1     19.40
TSS8     19.14
IF987    17.13
HH3      16.57
TSS16    16.18
GG2      16.08
HH4      14.42
```

## Confidence Intervals 95%

| CI Levels | OR | Lower Interval | Upper Interval |
|---|---|---|---|
| (Intercept) | 15.92 | 15.719 | 16.103 |
| MM2 | 1.072 | 0.885 | 1.287 |
| MM3 | 1.034 | 0.928 | 1.211 |
| MM4 | 1.023 | 0.88 | 1.192 |
| MM5 | 0.951 | 0.812 | 1.11 |
| MM6 | 1.16 | 0.026 | NA |
| MM9 | 0.976 | 0.846 | 1.126 |
| Yy2008 | 1.05 | 0.912 | 1.211 |
| Yy2009 | 1.143 | 0.989 | 1.324 |
| Yy2010 | 1.139 | 0.983 | 1.323 |
| Yy2011 | 1.119 | 0.965 | 1.3 |
| Yy2012 | 1.155 | 0.992 | 1.348 |
| PC501 | 1.07 | 0.766 | 1.414 |
| PC502 | 1.077 | 0.665 | 1.595 |
| PC503 | 1.121 | 0.778 | 1.519 |
| PC504 | 1.266 | 0.617 | 2.25 |
| PC505 | 1.154 | 0.777 | 1.6 |
| PC506 | 0.998 | 0.869 | 1.172 |
| PC508 | 1.174 | 0.633 | 1.929 |

| | | | |
|---|---|---|---|
| PC509 | 1.021 | 0.675 | 1.429 |
| GG2 | 1.074 | 0.918 | 1.254 |
| GG3 | 1.058 | 0.898 | 1.247 |
| GG4 | 1.02 | 0.929 | 1.184 |
| GG9 | 0.716 | 0.637 | 0.805 |
| IF10 | 0.001 | NA | 8.13E+11 |
| IF20 | 1.037 | 0.887 | 1.21 |
| IF30 | 1.021 | 0.908 | 1.191 |
| IF40 | 1.004 | 0.902 | 1.187 |
| IF50 | 0.974 | 0.885 | 1.112 |
| IF60 | 0.718 | 0 | 0 |
| IF987 | 1.074 | 0.944 | 1.268 |
| SSt1 | 0.001 | NA | 2.76E+13 |
| SSt2A | 0.004 | NA | 2.89E+10 |
| SSt2B | 0.027 | NA | 4268919 |
| WNR2 | 0.197 | NA | 1.294 |
| BB3 | NA | NA | NA |
| RB | 0.961 | 0.855 | 1.084 |
| RO | 0.991 | 0.883 | 1.146 |
| RU | 1.033 | 0.93 | 1.193 |
| INI2 | 0.925 | 0.644 | 1.219 |
| INI3 | 1.014 | 0.672 | 1.369 |
| INI9 | 0.965 | 0.792 | 1.143 |
| AR2 | 1.352 | 0.025 | 4.841 |
| AR3 | 1.442 | 0.011 | 6.808 |
| AR4 | 0.845 | 0.018 | 2.897 |
| AUK | NA | NA | NA |
| HH10 | 0.99 | 0.837 | 1.18 |
| HH11 | 1.06 | 0.862 | 1.286 |
| HH12 | 0.874 | 0.759 | 1.008 |
| HH2 | 0.906 | 0.815 | 1.032 |
| HH3 | 1.076 | 0.929 | 1.275 |
| HH4 | 0.945 | 0.828 | 1.093 |
| HH5 | 0.835 | 0.607 | 1.116 |
| HH6 | 1.095 | 0.942 | 1.299 |
| HH7 | 1.006 | 0.901 | 1.152 |
| HH8 | 0.982 | 0.886 | 1.111 |
| HH9 | 0.634 | 0.515 | 0.768 |
| TSS1 | 1.1 | 0.926 | 1.304 |
| TSS10 | 0.983 | 0.902 | 1.128 |
| TSS11 | 0.985 | 0.909 | 1.12 |

| | | | |
|---|---|---|---|
| TSS12 | 0.975 | 0.902 | 1.081 |
| TSS13 | 0.985 | 0.917 | 1.132 |
| TSS14 | 0.931 | 0.859 | 1.021 |
| TSS15 | 1.229 | 0.059 | NA |
| TSS16 | 0.962 | 0.896 | 1.082 |
| TSS17 | 1.009 | 0.936 | 1.292 |
| TSS2 | 1.054 | 0.887 | 1.252 |
| TSS21 | 0.863 | 0.766 | 0.973 |
| TSS3 | 1.049 | 0.895 | 1.235 |
| TSS4 | 0.974 | 0.837 | 1.139 |
| TSS5 | 1.004 | 0.877 | 1.156 |
| TSS6 | 0.964 | 0.859 | 1.099 |
| TSS7 | 0.994 | 0.888 | 1.149 |
| TSS8 | 0.945 | 0.859 | 1.058 |
| TSS9 | 0.942 | 0.864 | 1.05 |

# Section 3

## Validation Results

**Where: T1 = BCS; T2 = BCSR**

**Bayesian Network**

```
Confusion Matrix and Statistics

          Reference
Prediction   T1   T2
        T1  253  539
        T2   33 2694

               Accuracy : 0.8375
                 95% CI : (0.8248, 0.8495)
    No Information Rate : 0.9187
    P-Value [Acc > NIR] : 1

                  Kappa : 0.3974
 Mcnemar's Test P-Value : <2e-16

            Sensitivity : 0.88462
            Specificity : 0.83328
         Pos Pred Value : 0.31944
         Neg Pred Value : 0.98790
             Prevalence : 0.08127
         Detection Rate : 0.07190
   Detection Prevalence : 0.22506
      Balanced Accuracy : 0.85895

       'Positive' Class : T1
```

**Logistic Regression**

```
Confusion Matrix and Statistics

          Reference
Prediction   T1   T2
        T1  165  627
        T2   10 2717

               Accuracy : 0.819
                 95% CI : (0.8059, 0.8316)
    No Information Rate : 0.9503
    P-Value [Acc > NIR] : 1

                  Kappa : 0.2828
 Mcnemar's Test P-Value : <2e-16

            Sensitivity : 0.94286
            Specificity : 0.81250
         Pos Pred Value : 0.20833
         Neg Pred Value : 0.99633
             Prevalence : 0.04973
         Detection Rate : 0.04689
   Detection Prevalence : 0.22506
      Balanced Accuracy : 0.87768

       'Positive' Class : T1
```

## Section 4

**Logistic Regression**
**San Francisco-Oakland**

```
Call:
NULL

Deviance Residuals:
    Min       1Q   Median       3Q      Max
-1.4515  -0.4910   0.3129   0.4135   1.6417

Coefficients:
              Estimate Std. Error z value Pr(>|z|)
(Intercept)  1.598e+00  5.927e+00   0.270 0.787434
MM2          5.162e-02  5.920e-02   0.872 0.383230
MM3          9.245e-03  4.389e-02   0.211 0.833160
MM4          8.759e-02  5.314e-02   1.649 0.099243 .
MM5         -3.595e-02  5.302e-02  -0.678 0.497713
MM6          4.249e-02  7.029e-02   0.604 0.545520
MM9          2.637e-02  4.627e-02   0.570 0.568752
Yy2008      -1.742e-02  5.425e-02  -0.321 0.748160
Yy2009      -3.040e-02  5.439e-02  -0.559 0.576146
Yy2010       5.261e-03  5.572e-02   0.094 0.924771
Yy2011      -2.028e-02  5.653e-02  -0.359 0.719773
Yy2012      -7.622e-02  5.449e-02  -1.399 0.161863
PC501       -1.487e-02  1.218e-01  -0.122 0.902838
PC502        3.688e-02  2.069e-01   0.178 0.858524
PC503        2.698e-02  1.484e-01   0.182 0.855728
PC504        4.254e-02  2.889e-01   0.147 0.882931
PC505       -2.196e-02  1.648e-01  -0.133 0.893979
PC506        3.823e-02  6.309e-02   0.606 0.544572
PC508        2.555e-02  2.487e-01   0.103 0.918179
PC509       -7.498e-02  1.477e-01  -0.508 0.611769
GG2         -1.420e-02  5.527e-02  -0.257 0.797182
GG3          5.756e-02  5.709e-02   1.008 0.313349
GG4          5.759e-02  4.509e-02   1.277 0.201517
GG9         -1.953e-01  4.189e-02  -4.662 3.13e-06 ***
IF10        -7.522e+00  1.186e+03  -0.006 0.994940
IF20         1.144e-01  5.736e-02   1.994 0.046184 *
IF30        -4.565e-03  4.836e-02  -0.094 0.924789
IF40        -4.370e-02  4.050e-02  -1.079 0.280524
IF50        -2.716e-02  4.529e-02  -0.600 0.548764
IF60         7.924e-03  5.551e+01   0.000 0.999886
IF987       -4.226e-02  3.693e-02  -1.145 0.252414
SSt1         9.431e+00  1.936e+03   0.005 0.996112
SSt2A        7.164e+00  1.496e+03   0.005 0.996179
SSt2B        4.437e+00  9.307e+02   0.005 0.996196
WNR2        -2.779e+00  3.745e+01  -0.074 0.940848
BB3         -1.535e+01  2.083e+03  -0.007 0.994119
RB          -7.412e-02  4.167e-02  -1.779 0.075270 .
RO           3.470e-02  4.444e-02   0.781 0.434908
RU          -4.580e-02  3.719e-02  -1.232 0.218063
INI2         3.431e-01  2.048e-01   1.675 0.093885 .
```

```
INI3         3.184e-01  2.055e-01   1.549 0.121330
INI9         2.334e-02  4.831e-02   0.483 0.629016
AR2         -4.557e-02  6.061e-01  -0.075 0.940067
AR3          3.577e-02  7.089e-01   0.050 0.959756
AR4         -3.606e-01  5.296e-01  -0.681 0.495899
AUK         -2.621e-01  3.922e+01  -0.007 0.994669
HH10        -1.029e-01  7.275e-02  -1.415 0.157169
HH11        -5.170e-02  9.630e-02  -0.537 0.591322
HH12        -1.165e-01  5.829e-02  -1.998 0.045689 *
HH2         -1.930e-02  5.538e-02  -0.349 0.727435
HH3          1.737e-02  5.095e-02   0.341 0.733196
HH4         -3.494e-02  5.653e-02  -0.618 0.536480
HH5         -2.202e-01  1.378e-01  -1.598 0.110150
HH6          2.287e-02  5.294e-02   0.432 0.665808
HH7         -3.270e-02  4.146e-02  -0.789 0.430388
HH8         -3.768e-02  4.089e-02  -0.922 0.356779
HH9         -3.100e-01  8.325e-02  -3.723 0.000197 ***
TSS1         1.225e-01  5.940e-02   2.063 0.039106 *
TSS10        5.094e-02  4.361e-02   1.168 0.242739
TSS11        2.489e-02  4.012e-02   0.620 0.534994
TSS12       -1.590e-02  3.243e-02  -0.490 0.623884
TSS13       -3.004e-03  3.538e-02  -0.085 0.932336
TSS14        9.833e-03  3.185e-02   0.309 0.757535
TSS15        4.420e-02  5.553e-02   0.796 0.426002
TSS16       -2.396e-01  3.842e+01  -0.006 0.995023
TSS17        7.836e-03  3.702e-02   0.212 0.832379
TSS2         1.503e-01  6.042e-02   2.488 0.012841 *
TSS21       -2.499e-02  4.235e-02  -0.590 0.555141
TSS3         1.385e-01  5.794e-02   2.390 0.016840 *
TSS4         1.708e-01  5.838e-02   2.926 0.003433 **
TSS5         1.286e-01  5.252e-02   2.449 0.014331 *
TSS6         5.049e-02  4.518e-02   1.118 0.263766
TSS7         1.098e-01  5.092e-02   2.156 0.031051 *
TSS8         3.478e-02  4.152e-02   0.838 0.402174
TSS9         9.697e-02  5.539e-02   1.751 0.079994 .
---
Signif. codes:  0 ‘***’ 0.001 ‘**’ 0.01 ‘*’ 0.05 ‘.’ 0.1 ‘ ’ 1

(Dispersion parameter for binomial family taken to be 1)

    Null deviance: 4604.2  on 9729  degrees of freedom
Residual deviance: 3914.5  on 9655  degrees of freedom
AIC: 1914.2

Number of Fisher Scoring iterations: 15
```

**glm variable importance**

| | Overall |
|---|---|
| GG9 | 100.00 |
| HH9 | 79.87 |
| TSS4 | 62.76 |
| TSS2 | 53.37 |
| TSS5 | 52.53 |
| TSS3 | 51.27 |
| TSS7 | 46.26 |
| TSS1 | 44.25 |
| HH12 | 42.86 |
| IF20 | 42.77 |
| RB | 38.16 |
| TSS9 | 37.55 |
| INI2 | 35.93 |
| MM4 | 35.36 |
| HH5 | 34.27 |
| INI3 | 33.23 |
| HH10 | 30.34 |
| Yy2012 | 30.00 |
| GG4 | 27.40 |
| RU | 26.42 |

## Section 5

### San Francisco-Oakland Validation Results

**T1 = BCS; T2 =BCSR**

**Bayesian Network**

```
Confusion Matrix and Statistics

          Reference
Prediction   T1   T2
        T1  332  911
        T2   87 2836

               Accuracy : 0.7604
                 95% CI : (0.7472, 0.7733)
    No Information Rate : 0.8994
    P-Value [Acc > NIR] : 1

                  Kappa : 0.2932
 Mcnemar's Test P-Value : <2e-16

            Sensitivity : 0.79236
            Specificity : 0.75687
         Pos Pred Value : 0.26710
         Neg Pred Value : 0.97024
             Prevalence : 0.10058
         Detection Rate : 0.07969
   Detection Prevalence : 0.29837
      Balanced Accuracy : 0.77462

       'Positive' Class : T1
```

**Logistic Regression**

```
Confusion Matrix and Statistics

          Reference
Prediction   T1   T2
        T1  244  999
        T2   16 2907

               Accuracy : 0.7564
                 95% CI : (0.743, 0.7693)
    No Information Rate : 0.9376
    P-Value [Acc > NIR] : 1

                  Kappa : 0.2469
 Mcnemar's Test P-Value : <2e-16

            Sensitivity : 0.93846
            Specificity : 0.74424
         Pos Pred Value : 0.19630
         Neg Pred Value : 0.99453
             Prevalence : 0.06241
         Detection Rate : 0.05857
   Detection Prevalence : 0.29837
      Balanced Accuracy : 0.84135

       'Positive' Class : T1
```

## Section 6

**Logistic Regression**
**Atlanta**

```
Call:
NULL

Deviance Residuals:
    Min       1Q   Median       3Q      Max
-1.4647   0.0000   0.2654   0.3592   1.3985

Coefficients:
                Estimate   Std. Error z value   Pr(>|z|)
(Intercept)    2.0189544   14.3513952   0.141    0.88812
MM2            0.1078399    0.0917869   1.175    0.24004
MM3           -0.0430897    0.0548820  -0.785    0.43238
MM4            0.0239916    0.0780620   0.307    0.75858
MM5           -0.0489998    0.0789751  -0.620    0.53496
MM6           -0.0238757    0.0645190  -0.370    0.71134
MM9           -0.0537853    0.0594797  -0.904    0.36586
Yy2008        -0.0007928    0.0869514  -0.009    0.99273
Yy2009        -0.0021342    0.0883807  -0.024    0.98073
Yy2010        -0.2511589    0.0803288  -3.127    0.00177 **
Yy2011        -0.3611193    0.0806398  -4.478 0.00000753 ***
Yy2012        -0.0865604    0.0876093  -0.988    0.32314
PC501          0.1105761    0.1758027   0.629    0.52936
PC502          0.1798431    0.2786421   0.645    0.51865
PC503          0.1282636    0.2015242   0.636    0.52447
PC504          0.1832537    0.3830486   0.478    0.63236
PC505          0.0889591    0.2133936   0.417    0.67677
PC506         -0.0217680    0.0800558  -0.272    0.78569
PC508          0.1487771    0.3379894   0.440    0.65980
PC509         -0.0597168    0.2069333  -0.289    0.77290
GG2            0.0398265    0.0852811   0.467    0.64050
GG3           -0.0084892    0.0874128  -0.097    0.92263
GG4            0.0493362    0.0882207   0.559    0.57600
GG9           -0.2611667    0.0635589  -4.109 0.00003973 ***
IF10           0.6849793    0.5963874   1.149    0.25074
IF20          -0.0445568    0.0847281  -0.526    0.59897
IF30           0.0552940    0.0886141   0.624    0.53264
IF40           0.0361523    0.1095888   0.330    0.74148
IF50          -0.0059177    0.0660479  -0.090    0.92861
IF60          -0.5609199   71.8032012  -0.008    0.99377
IF987          0.1001467    0.0780486   1.283    0.19945
SSt1          -5.4057441  270.8565768  -0.020    0.98408
SSt2A         -4.2963259  206.6594999  -0.021    0.98341
SSt2B         -2.5562514  122.2573229  -0.021    0.98332
WNR2          -3.1839043  101.9066306  -0.031    0.97508
WUnk          -0.4492853   83.8623004  -0.005    0.99573
BB3            6.0413700  246.2134765   0.025    0.98042
RB            -0.0366593    0.0669146  -0.548    0.58379
RO            -0.0223963    0.0630841  -0.355    0.72257
RU            -0.0528441    0.0465408  -1.135    0.25619
```

```
INI2          0.0072477    0.1136937   0.064    0.94917
INI3          0.1179756    0.1204280   0.980    0.32727
INI9         -0.0539609    0.0619484  -0.871    0.38372
AR2           8.2850005 2988.9834706   0.003    0.99779
AR3           9.1175594 3282.5208311   0.003    0.99778
AR4           5.5661009 2098.5099795   0.003    0.99788
HH10         -0.0049155    0.0893406  -0.055    0.95612
HH11          0.0407288    0.1078896   0.378    0.70580
HH12         -0.0736401    0.0824961  -0.893    0.37205
HH2          -0.0545573    0.0659381  -0.827    0.40801
HH3           0.0386547    0.0784191   0.493    0.62207
HH4          -0.0597897    0.0745831  -0.802    0.42275
HH5          -0.0982818    0.1657945  -0.593    0.55332
HH6           0.1878216    0.0945893   1.986    0.04707 *
HH7          -0.0000835    0.0653396  -0.001    0.99898
HH8          -0.0254737    0.0575806  -0.442    0.65820
HH9          -0.2880435    0.1046327  -2.753    0.00591 **
TSS1          0.1168892    0.0852531   1.371    0.17035
TSS10         0.0180732    0.0572603   0.316    0.75228
TSS11        -0.0283440    0.0510323  -0.555    0.57861
TSS12        -0.0247849    0.0449620  -0.551    0.58147
TSS13         0.0107678    0.0599137   0.180    0.85737
TSS14         0.3006751   58.0360155   0.005    0.99587
TSS15         0.3746772   57.0357329   0.007    0.99476
TSS16         0.2000648   58.4147456   0.003    0.99727
TSS17         0.3516671   58.2473379   0.006    0.99518
TSS2          0.1346616    0.0881495   1.528    0.12660
TSS21        -0.0953492    0.0640693  -1.488    0.13669
TSS3          0.0152543    0.0810591   0.188    0.85073
TSS4          0.0409127    0.0795903   0.514    0.60722
TSS5         -0.0112807    0.0721977  -0.156    0.87584
TSS6         -0.0129015    0.0608755  -0.212    0.83216
TSS7         -0.0107321    0.0562429  -0.191    0.84867
TSS8         -0.0216080    0.0543248  -0.398    0.69081
TSS9         -0.0450524    0.0463310  -0.972    0.33085
---
Signif. codes:  0 ‘***’ 0.001 ‘**’ 0.01 ‘*’ 0.05 ‘.’ 0.1 ‘ ’ 1

(Dispersion parameter for binomial family taken to be 1)
```

**glm variable importance**

| | Overall |
|---|---|
| Yy2011 | 100.00 |
| GG9 | 91.75 |
| Yy2010 | 69.81 |
| HH9 | 61.46 |
| HH6 | 44.32 |
| TSS2 | 34.09 |
| TSS21 | 33.21 |
| TSS1 | 30.60 |
| IF987 | 28.63 |
| MM2 | 26.21 |
| IF10 | 25.63 |
| RU | 25.33 |
| Yy2012 | 22.04 |
| INI3 | 21.85 |
| TSS9 | 21.69 |
| MM9 | 20.17 |
| HH12 | 19.91 |
| INI9 | 19.43 |
| HH2 | 18.45 |
| HH4 | 17.88 |

**Section 7**

**Atlanta Validation Results**

**T1 = BCS; T2 =BCSR**

**Bayesian Network**

```
Confusion Matrix and Statistics

          Reference
Prediction   T1   T2
        T1  107  473
        T2   63 1722

               Accuracy : 0.7734
                 95% CI : (0.7559, 0.7901)
    No Information Rate : 0.9281
    P-Value [Acc > NIR] : 1

                  Kappa : 0.1959
 Mcnemar's Test P-Value : <2e-16

            Sensitivity : 0.62941
            Specificity : 0.78451
         Pos Pred Value : 0.18448
         Neg Pred Value : 0.96471
             Prevalence : 0.07188
         Detection Rate : 0.04524
   Detection Prevalence : 0.24524
      Balanced Accuracy : 0.70696

       'Positive' Class : T1
```

**Logistic Regression**

```
Confusion Matrix and Statistics

          Reference
Prediction   T1   T2
        T1   94  486
        T2    9 1776

               Accuracy : 0.7907
                 95% CI : (0.7737, 0.8069)
    No Information Rate : 0.9564
    P-Value [Acc > NIR] : 1

                  Kappa : 0.2174
 Mcnemar's Test P-Value : <0.0000000000000002

            Sensitivity : 0.91262
            Specificity : 0.78515
         Pos Pred Value : 0.16207
         Neg Pred Value : 0.99496
             Prevalence : 0.04355
         Detection Rate : 0.03975
   Detection Prevalence : 0.24524
      Balanced Accuracy : 0.84888

       'Positive' Class : T1
```
